# The Proteins of Severe Acute Respiratory Syndrome Coronavirus-2 (SARS CoV-2 or n-COV19), the Cause of COVID-19

**DOI:** 10.1007/s10930-020-09901-4

**Published:** 2020-05-23

**Authors:** Francis K. Yoshimoto

**Affiliations:** Department of Chemistry, The University of Texas at San Antonio (UTSA), San Antonio, TX 78249-0698 USA

**Keywords:** Proteins, Virus, SARS CoV-2

## Abstract

The devastating effects of the recent global pandemic (termed COVID-19 for “coronavirus disease 2019”) caused by the severe acute respiratory syndrome coronavirus-2 (SARS CoV-2) are paramount with new cases and deaths growing at an exponential rate. In order to provide a better understanding of SARS CoV-2, this article will review the proteins found in the SARS CoV-2 that caused this global pandemic.

**Electronic supplementary material:**

The online version of this article (10.1007/s10930-020-09901-4) contains supplementary material, which is available to authorized users.

## Introduction

Severe acute respiratory syndrome coronavirus-2 (SARS CoV-2) is the virus that caused the global pandemic that was first reported [1] on December 31, 2019 [2]. Taxonomically, SARS CoV-2 belongs to the realm *Riboviria*, order *Nidovirales*, suborder *Cornidovirineae*, family *Coronaviridae*, subfamily *Orthocoronavirinae*, genus *Betacoronavirus* (lineage B), [3] subgenus *Sarbecovirus*, and the species *Severe acute respiratory syndrome-related coronavirus*.

The genome of SARS CoV-2 (NCBI Reference Sequence: NC_045512.2) [4] is similar to the genome of the coronavirus that caused the SARS epidemic in 2003 (SARS CoV, NCBI Reference sequence: NC_004718.3) [5, 6]. Much of the understanding of the proteins found in SARS CoV-2 are based on the numerous research studies reported on SARS CoV and other related viruses (e.g. MERS CoV) [7, 8]. However, among the recent coronavirus outbreaks in the new millennium (SARS CoV: 2002–2003, MERS CoV: 2012, SARS CoV-2: 2020), SARS CoV-2 mysteriously had the most devastating global impact. Understanding the proteins present in these viruses enable a more rational approach to designing more effective antiviral drugs [9, 10]. The majority of proteins of SARS CoV have been characterized in detail. The proteins of SARS CoV consist of two large polyproteins: ORF1a and ORF1ab (that proteolytically cleave to form 16 nonstructural proteins), four structural proteins: spike (S), envelope (E), membrane (M), and nucleocapsid (N), and eight accessory proteins: ORF3a, ORF3b (NP_828853.1, not present in SARS CoV-2), ORF6, ORF7a, ORF7b, ORF8a, ORF8b, and ORF9b (NP_828859.1, not present in SARS CoV-2). Although accessory proteins have been viewed as dispensable for viral replication in vitro, some have been shown to play an important role in virus-host interactions in vivo [11]. Similar to SARS CoV, SARS CoV-2 lacks the hemagglutinin esterase gene, which is found in human coronavirus (hCoV) HKU1, a lineage A betacoronavirus [3]. The spike protein, envelope protein, membrane protein, nucleocapsid protein, 3CL protease, papain like protease, RNA polymerase, [10] and helicase protein have been suggested to be viable antiviral drug targets [12]. SARS CoV-2 is an RNA virus and its RNA genome is 30 kb in length. SARS CoV-2 is thought to have originated from its closest relative, BatCov RaTG13 (GenBank: MN996532), [13] which was isolated from horseshoe bats [14].

## Discussion: Proteins of SARS CoV-2

SARS CoV-2 (NC_045512.2) has a total of 11 genes with 11 open reading frames (ORFs) (Table 1): ORF1ab, ORF2 (Spike protein), ORF3a, ORF4 (Envelope protein), ORF5 (Membrane protein), ORF6, ORF7a, ORF7b, ORF8, ORF9 (Nucleocapsid protein), and ORF10.

**Table 1:** The genes expressed by SARS CoV-2 (NC_045512.2)

Number(#)	Gene	GeneID	Location	Protein	[LOCUS]
1(7,096)	ORF1ab	43,740,578	266–21,555	ORF1ab polyprotein	[BCB15089.1/BCB97900.1]
1(4,405)	ORF1a	43,740,578	266–13,483	ORF1a polyprotein	[YP_009725295.1]
2(1,273)	ORF2 (S)	43,740,568	21,563–25,384	Spike protein (S protein)	[BCA87361.1]
3(275)	ORF3a	43,740,569	25,393–26,220	ORF3a protein	[BCA87362.1]
4(75)	ORF4 (E)	43,740,570	26,245–26,472	Envelope protein (E protein)	[BCA87363.1]
5(222)	ORF5 (M)	43,740,571	26,523–27,191	Membrane protein (M protein)	[BCA87364.1]
6(61)	ORF6	43,740,572	27,202–27,387	ORF6 protein	[BCA87365.1]
7(121)	ORF7a	43,740,573	27,394–27,759	ORF7a protein	[BCA87366.1]
8(43)	ORF7b	43,740,574	27,756–27,887	ORF7b protein	[BCB15096.1]
9(121)	ORF8	43,740,577	27,894–28,259	ORF8 protein	[BCA87367.1]
10(419)	ORF9 (N)	43,740,575	28,274–29,533	Nucleocapsid phosphoprotein (N protein)	[BCA87368.1]
11(38)	ORF10	43,740,576	29,558–29,674	ORF10 protein	[BCA87369.1]

#Represents the number of amino acids in each gene

### Polyprotein Expressed by ORF1ab

The first gene (ORF1ab) expresses a polyprotein. The ORF1ab polyprotein is comprised of 16 nonstructural proteins (NSPs) (Table 2).

**Table 2:** The nonstructural proteins (NSPs) found in the polyprotein of SARS CoV-2

#	Name	Accession	Amino acids	Proposed function
(i)	NSP1	YP_009725297.1	180 amino acids	Induce host mRNA (leader protein) cleavage
(ii)	NSP2	YP_009725298.1	638 amino acids	Binds to PHBs 1, 2
(iii)	NSP3^a^	YP_009725299.1	1945 amino acids	Release NSPs 1, 2, 3 (Papain like proteinase)
(iv)	NSP4	YP_009725300.1	500 amino acids	Membrane rearrangement
(v)	NSP5^a^	YP_009725301.1	306 amino acids	Cleaves at 11 sites of (3C-like proteinase) NSP polyprotein
(vi)	NSP6	YP_009725302.1	290 amino acids	Generates autophagosomes
(vii)	NSP7	YP_009725303.1	83 amino acids	Dimerizes with NSP8
(viii)	NSP8	YP_009725304.1	198 amino acids	Stimulates NSP12
(ix)	NSP9	YP_009725305.1	113 amino acids	Binds to helicase(?)
(x)	NSP10	YP_009725306.1	139 amino acids	Stimulates NSP16(?)
(xi)	NSP11	YP_009725312.1	13 amino acids	Unknown
(xii)	NSP12^a^	YP_009725307.1	932 amino acids	Copies viral RNA (RNA polymerase) methylation (guanine)
(xiii)	NSP13	YP_009725308.1	601 amino acids	Unwinds duplex RNA (Helicase)
(xiv)	NSP14	YP_009725309.1	527 amino acids	5′-cap RNA (3′ to 5′ exonuclease, guanine N7-methyltransferase)
(xv)	NSP15^a^	YP_009725310.1	346 amino acids	Degrade RNA to (endoRNAse/endoribonuclease) evade host defense
(xvi)	NSP16	YP_009725311.1	298 amino acids	5′-cap RNA (2′-O-ribose-methyltransferase—potential antiviral drug target) methylation (adenine)

^a^Indicates possible targets of antiviral compounds

#### NSP1 (Leader Protein)

**Fig. 1:**
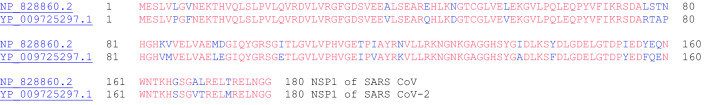
Alignment of the primary amino acid sequence of NSP1 of SARS CoV (top, NP_828860.2) and SARS CoV-2 (YP_009725297.1). Sequence identity: 84.4%. Sequence similarity: 93.9%—determined using LALIGN software (and for subsequent alignments, Figs. 2, 3, 4, 5, 6, 7, 8, 9, 10, 11, 12, 13, 14, 15, 16, 17, 18, 19, 20, 21, 22, 23, 24, 25, and 26, see Supporting Information for output data) [16].

Nonstructural protein 1 (NSP1) is the first protein of the polyprotein of SARS CoV-2 (Fig. 1—sequence alignment of NSP1 for SARS CoV with SARS CoV-2). This protein is also known as the leader protein. This protein is also found in SARS coronavirus and is known to be a potent inhibitor of host gene expression. NSP1 binds to the 40S ribosome of the host cell to inactivate translation and promotes host mRNA degradation selectively, while the viral SARS CoV mRNA remain intact [15]. Figure 1 shows the amino acid sequence alignment for the NSP1 proteins of SARS CoV (from genome: NCBI Reference Sequence: NC_004718.3) and SARS CoV-2.

**Fig. 2:**
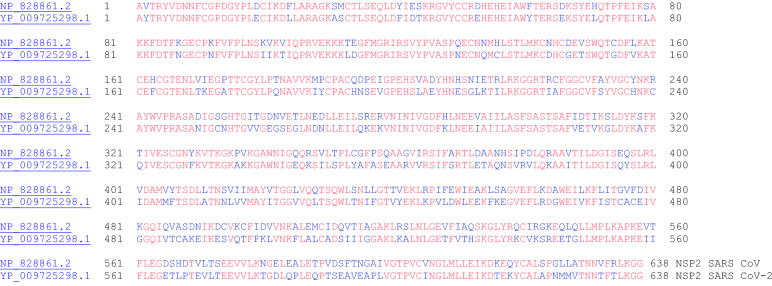
The primary amino acid sequence alignment of NSP2 for SARS CoV (NP_828861.2) and SARS CoV-2 (YP_009725298.1). These proteins have 68.3% sequence identity (90.0% similar)

#### NSP2

Nonstructural protein 2 (NSP2) is the second protein of the polyprotein of SARS CoV-2 (Fig. 2). This protein is conserved in SARS CoV, the related beta coronavirus to SARS CoV-2. In SARS CoV, NSP2 was found to bind to two host proteins: prohibitin 1 and prohibitin 2 (PHB1 and PHB2) [17]. PHB1 and PHB2 proteins are known to play roles in cell cycle progression, cell migration, cellular differentiation, apoptosis, and mitochondrial biogenesis. The binding of NSP2 to PHB1 and PHB2 proteins suggest that NSP2 plays a role in disrupting the host cell environment.

#### NSP3 (Papain like Proteinase)

**Fig. 3:**
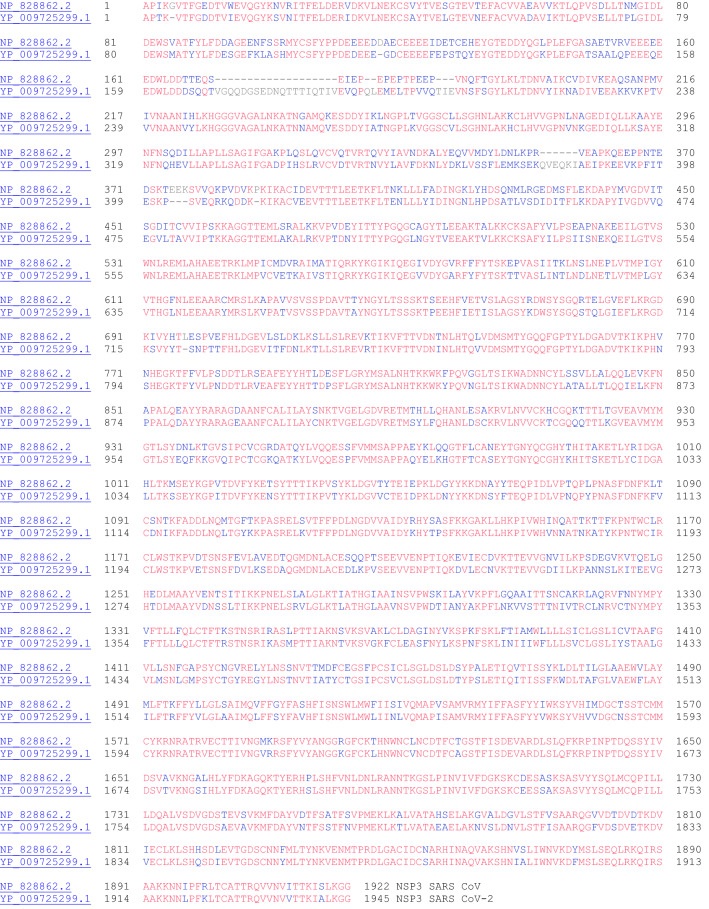
The primary amino acid sequence alignment of NSP3 for SARS CoV (NP_828862.2) and SARS CoV-2 (YP_009725299.1). Sequence identity: 76.0%, sequence similarity: 91.8%

NSP3 is the papain-like proteinase protein (Fig. 3). This protein is nearly 200 kDa in size and is the largest protein (not including the polyproteins ORF1a and ORF1ab) encoded by the coronaviruses. With such a long sequence, it possesses several conserved domains: ssRNA binding, ADPr binding, G-quadruplex binding, ssRNA binding, protease (papain-like protease), and NSP4 binding), and transmembrane domain. Among the 16 nonstructural proteins, NSP3, NSP4, and NSP6 have transmembrane domains [18]. The papain like protease 1 (PL1 protease) of alpha coronavirus (alpha CoV) Transmissible Gastroenteritis Virus (TGEV), which is part of NSP3, was shown to cleave the site between NSP2 and NSP3. Furthermore, this papain like protease domain is responsible for the release of NSP1, NSP2, and NSP3 from the N-terminal region of polyproteins 1a and 1ab from coronaviruses [19]. Considering this important protease activity to release essential proteins for viral activity, the inhibition of NSP3 protease activity is an important target for antiviral activity [20]. Tanshinones, a class of natural products have been found to inhibit NSP3 protease activity.

#### NSP4 (Contains Transmembrane Domain 2)

**Fig. 4:**
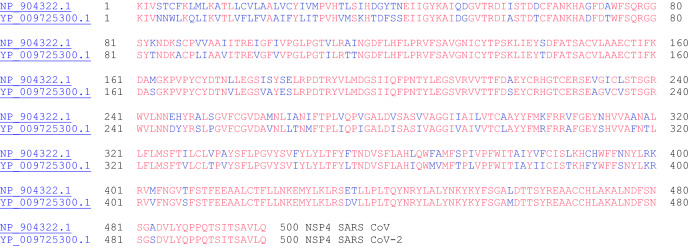
The primary amino acid sequence alignment of NSP4 for SARS CoV (NP_904322.1) and SARS CoV-2 (YP_009725300.1). Sequence identity: 80.0%, sequence similarity: 95.0%

NSP4 interacts with NSP3 and possibly host proteins to confer a role related to membrane rearrangement in SARS CoV. Moreover, the interaction between NSP4 and NSP3 is essential for viral replication [18]. The sequence alignment for NSP4 proteins for SARS CoV and SARS CoV-2 is shown in Fig. 4.

#### NSP5 (3C-like proteinase)

**Fig. 5:**
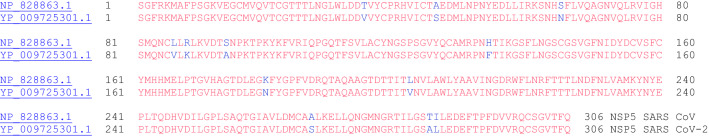
The primary amino acid sequence of NSP5 for SARS CoV (NP_828863.1) and SARS CoV-2 (YP_009725301.1). Sequence identity: 96.1%, sequence similarity: 99.7%

The NSP5 protein based on the Middle East Respiratory Syndrome (MERS) coronavirus has been characterized. NSP5 cleaves at 11 distinct sites to yield mature and intermediate nonstructural proteins (NSPs) [21]. The amino acid sequence alignment for NSP5 of SARS CoV and SARS CoV-2 is shown in Fig. 5.

#### NSP6 (Putative Transmembrane Domain)

**Fig. 6:**
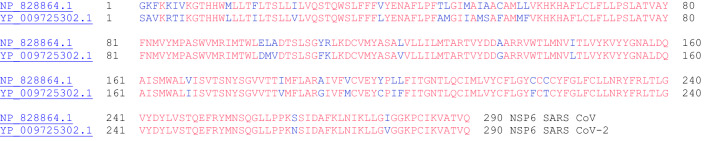
Amino acid sequence alignment between the NSP6 proteins of SARS CoV (top: NP_828864.1) and SARS-CoV-2 (bottom: YP_009725302.1). Sequence identity: 88.2%, sequence similarity: 98.3%

The NSP6 protein of the avian coronavirus (infectious bronchitis virus, IBV) was shown to generate autophagosomes from the endoplasmic reticulum (ER) (Fig. 6b shows sequence alignment with SARS CoV-2 NSP6). Autophagosomes facilitate assembly of replicase proteins. Furthermore, NSP6 limited autophagosome/lysosome expansion, which in turn prevents autophagosomes from delivering viral components for degradation in lysosomes [22]. With SARS CoV, NSP6 was shown to induce membrane vesicles [23]. The amino acid sequence alignment for NSP6 of SARS CoV and SARS CoV-2 is shown in Fig. 6.

#### NSP7

**Fig. 7:**
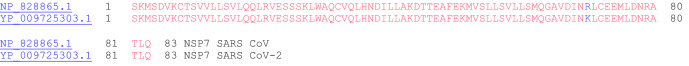
The primary amino acid sequence alignment of NSP7 SARS CoV (NP_828865.1) and SARS CoV-2 (YP_009725303.1). Sequence identity: 98.8%, sequence similarity: 100%

NSP7 is required to form a complex with NSP8 (next section) and NSP12 to yield the RNA polymerase activity of NSP8 [24]. The primary amino acid sequence alignment for the NSP8 proteins for SARS CoV and SARS CoV-2 is shown in Fig. 7. Only one amino acid residue is different (arginine vs. lysine) but the charge is conserved at this location.

#### NSP8

**Fig. 8:**
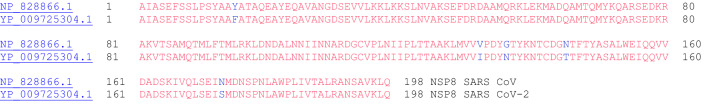
The primary amino acid sequence alignment of NSP8 for SARS CoV (NP_828866.1) and SARS CoV-2 (YP_009725304.1). Sequence identity: 97.5%, sequence similarity: 100.0%

NSP8 is a peptide cofactor that makes a heterodimer with NSP7 (the other peptide cofactor), and this NSP7-NSP8 heterodimer complexes with NSP12. In addition to the NSP7-NSP8 heterodimer, an NSP8 monomer unit also complexes with NSP12, which ultimately forms the RNA polymerase complex. The cryo-EM structure of this complex has been solved [25]. The amino acid sequence alignment for NSP8 of SARS CoV and SARS CoV-2 is shown in Fig. 8.

#### NSP9

**Fig. 9:**
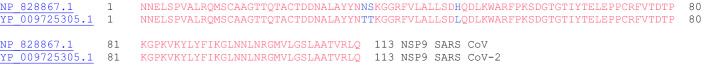
The primary amino acid sequence alignment of NSP9 for SARS CoV (NP_828868.1) and SARS CoV-2 (YP_009725305.1). Sequence identity: 97.3%, sequence similarity: 99.1%

NSP9 from the porcine reproductive and respiratory syndrome virus (PRRSV) has been found to interact with the DEAD-box RNA helicase 5 (DDX5) cellular protein [26]. This interaction between NSP9 and DDX5 has been shown to be important for viral replication—when the DDX5 gene was silenced in MARC-145 cells, the virus titers were lower by tenfold. Figure 9 shows the amino acid sequence alignment between the two NSP9 proteins from SARS CoV and SARS CoV-2.

#### NSP10

**Fig. 10:**
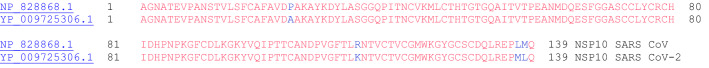
The primary amino acid sequence alignment of NSP10 for SARS CoV (NP_828868.1) and SARS CoV-2 (YP_009725306.1). Sequence identity: 97.1%, sequence similarity: 99.3%

NSP10 has been shown to interact with NSP14 in SARS coronavirus, and this interaction stimulates activity of NSP14. NSP 14 is known to function as an S-adenosylmethionine (SAM)-dependent (guanine-N7) methyl transferase (N7-MTase) [27]. Furthermore, NSP10 has also been shown to stimulate the activity of NSP16, which is a 2′-O-methyltransferase [28]. Figure 10 shows the amino acid sequence alignment between the two NSP10 proteins from SARS CoV and SARS CoV-2.

#### NSP11

**Fig. 11:**
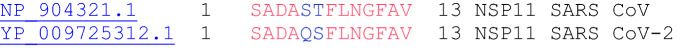
The primary amino acid sequence alignment of NSP11 for SARS CoV (NP_904321.1) and SARS CoV-2 (YP_009725312.1). Sequence identity: 84.6%, sequence similarity: 100.0%

The function of NSP11 seems to be unknown. NSP11 is made of thirteen amino acids and the first nine amino acids (sadaqsfln) are identical to the first nine in NSP12. Figure 11 shows the amino acid sequence alignment between the two NSP12 proteins from SARS CoV and SARS CoV-2.

#### NSP12 (RNA Dependent RNA Polymerase)

**Fig. 12:**
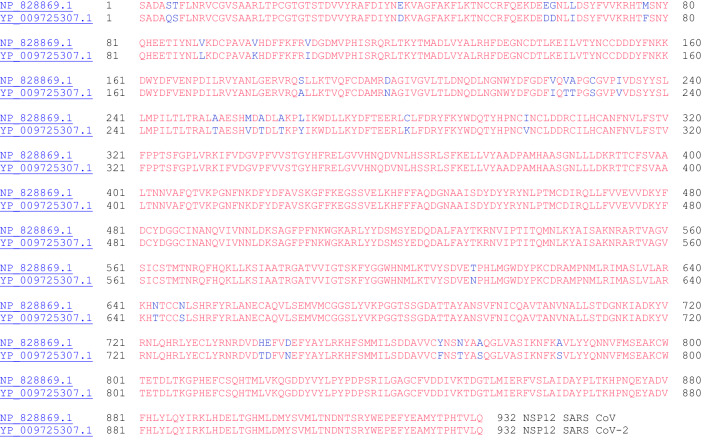
The primary amino acid sequence alignment of NSP12 for SARS CoV (NP_828869.1) and SARS CoV-2 (YP_009725307.1). Sequence identity: 96.4%, sequence similarity: 99.4%

NSP12 is the RNA-dependent RNA polymerase that copies viral RNA. As mentioned, NSP12 makes a complex with an NSP7-NSP8 heterodimer and an NSP8 monomer to confer processivity of NSP12. NSP12 exhibits poor processivity in RNA synthesis—that is the presence of NSP7 and NSP8 lowers the dissociation rate of NSP12 from RNA [29]. The amino acid sequence alignment between the two NSP12 proteins from SARS CoV and SARS CoV-2 is shown in Fig. 12.

#### NSP13 (Helicase)

**Fig. 13:**
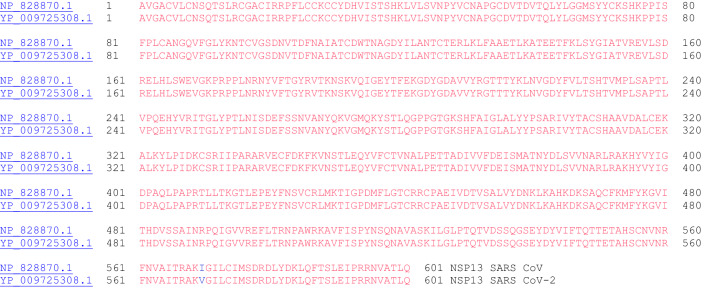
The primary amino acid sequence of NSP13 SARS CoV (NP_828870.1) and SARS CoV-2 (YP_009725308.1). Sequence identity: 99.8%, sequence similarity: 100.0%

SARS CoV was used to characterize the helicase enzyme, NSP13, which unwinds duplex RNA [30]. The crystal structure of NSP13 of SARS CoV has been reported [31]. Furthermore, it has been shown that binding of NSP12 with NSP13 can enhance the helicase activity of NSP13. In addition to its helicase activity, NSP13 of SARS CoV is also known to possess 5′-triphosphatase activity, which is responsible for introducing the 5′-terminal cap of the viral mRNA [32]. Both eukaryotic and most viral mRNA have a 5′-terminal cap structure: m7G(5)ppp(5)N-. This 5′-terminal cap is the site of recognition for translation and plays a role in splicing, nuclear export, translation, and stability of mRNA. This process of incorporating the 5′-terminal cap will be discussed in the next section: (xiv) NSP14. The sequence alignment for NSP13 of SARS CoV and SARS CoV-2 is shown in Fig. 13. Interestingly, only one amino acid residue is different out of the 601 amino acids in these two proteins (isoleucine vs. valine).

#### NSP14 (3′ to 5′ Endonuclease, N7-Methyltransferase)

**Fig. 14:**
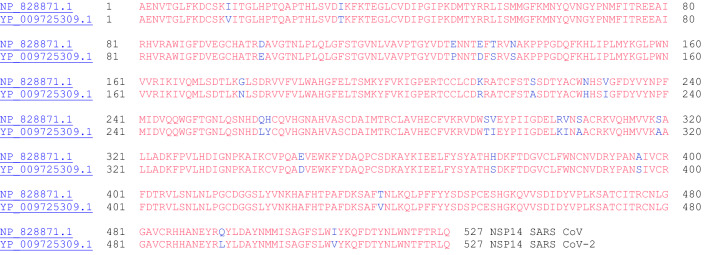
The primary amino acid sequence alignment of NSP14 of SARS CoV (NP_828871.1) and SARS CoV-2(YP_009725309.1). Sequence identity: 95.1%, sequence similarity: 99.1%

NSP14 from coronavirus is known to have 3′-5′ exoribonuclease activity and N7-methyltransferase activity [33]. The guanine-N7-methyltransferase activity is part of the process for introducing the 5′-cap of the virus, which involves multiple steps: [1] the gamma-phosphate of the 5′end of nascent mRNA is removed by the RNA triphosphatase (NSP13), [32], [2] a GMP moiety derived from a covalent enzyme-GMP intermediate is transferred to the resulting mRNA with a diphosphate end, [3] the GpppA cap is methylated with S-adenosyl-methionine, which is catalyzed by the guanine-N7-methyltransferase (NSP14) to yield the cap-0 structure, [34] and [4] 2′-O-methylation by NSP16 of adenine gives the cap-1 structure [35]. It is currently unknown which enzyme incorporates the GMP group involved in the second step, and it is possible that the virus uses the host guanylyltransferase enzyme [36]. Figure 14 shows the amino acid sequence alignment between the NSP14 proteins of SARS CoV and SARS CoV-2.

#### NSP15 (endoRNAse)

**Fig. 15:**
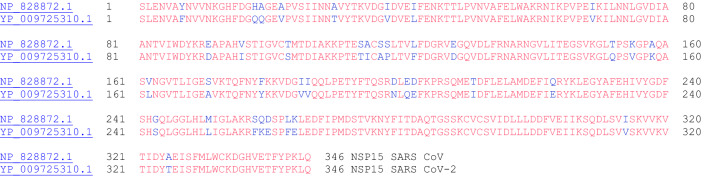
The primary amino acid sequence alignment of NSP15 of SARS CoV (NP_828872.1) and SARS CoV-2 (YP_009725310.1). Sequence identity: 88.7%, sequence similarity: 97.7%

NSP15 of SARS coronavirus has been biochemically characterized as an endoribonuclease that cleaves RNA at uridylates at the 3′-position to form a 2′-3′ cyclic phosphodiester product [37]. The NSP15 protein specifically targets and degrades the viral polyuridine sequences to prevent the host immune sensing system from detecting the virus [38]. The crystal structure of NSP15 has been reported for SARS CoV [39] and SARS CoV-2 [40]. NSP15 uses manganese as a cofactor to promote endoribonuclease activity [41]. It has been suggested that NSP15 degrades viral dsRNA to prevent host recognition [42]. The amino acid sequence alignment of NSP15 from SARS CoV and SARS CoV-2 is shown in Fig. 15.

#### NSP16 (2′-O-Ribose-Methyltransferase)

**Fig. 16:**
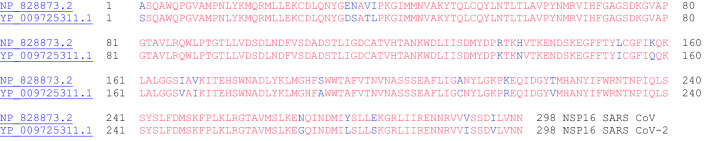
The primary amino acid sequence alignment of NSP16 of SARS CoV (NP_828873.2) and SARS CoV-2 (YP_009725311.1). Sequence identity: 93.3%, sequence similarity: 99.0%

NSP16 for coronavirus has been biochemically [43] (feline coronavirus, FCoV) and structurally [44] (complex of NSP10-NSP16 for SARS CoV) characterized. The viral RNA has a 5′-cap, which protects it from mRNA degradation by 5′-exoribonucleases, promotes mRNA translation, and prevents the viral RNA from being recognized by innate immunity mechanisms [44]. The RNA cap is an N7-methylated guanine nucleotide connected through a 5′-5′ triphosphate bridge to the first transcribed nucleotide (adenine). NSP16 methylates the 2′-hydroxy group of adenine using S-adenosylmethionine as the methyl source. Figure 16 shows the amino acid sequence alignment between the two NSP16 proteins from SARS CoV and SARS CoV-2.

### Spike Protein (Surface Glycoprotein)

**Fig. 17:**
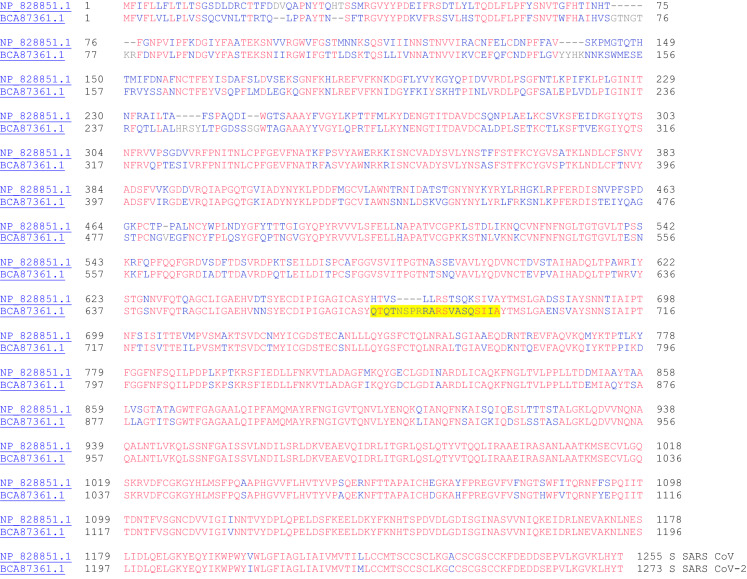
The primary amino acid sequence alignment of the spike proteins from SARS CoV (NP_828851.1) and SARS CoV-2 (BCA87361.1). Sequence identity: 76.0%, sequence similarity: 91.5%

The spike protein (Fig. 17—sequence alignment between SARS CoV and SARS CoV-2) is a glycoprotein, which mediates attachment of the virus to the host cell. The structure of the spike (S) protein has been determined. This protein recognizes the human angiotensin-converting enzyme 2 (ACE2) protein on the host cell surface [45–47]. SARS CoV spike mouse polyclonal antibodies potently inhibited SARS CoV-2 spike protein mediated entry into cells [47]. Interestingly, a furin cleavage site (highlighted in Fig. 17: QTQTNSPRRARSVASQSIIA) was located in the S protein of SARS CoV-2, which was lacking in the S protein of SARS CoV. This difference in site could possibly explain the difference in pathogenicity of these two viruses [47].

### ORF3a Protein

**Fig. 18:**
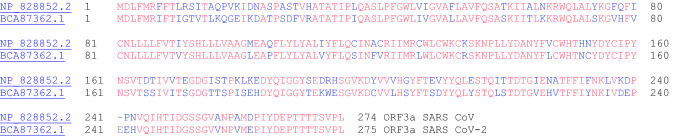
The primary amino acid sequence alignment of the ORF3a proteins from SARS CoV (NP_828852.2) and SARS CoV-2 (BCA87362.1). Sequence identity: 72.4%, sequence similarity: 90.2%

The ORF3a protein from SARS CoV is an ion channel protein related to NLRP3 inflammasome activation. ORF3a interacts with TRAF3, which in turn activates ASC ubiquitination, and as a result, leads to activation of caspase 1 and IL-1β maturation [48]. The amino acid sequence alignment between the two ORF3a proteins from SARS CoV and SARS CoV-2 is shown in Fig. 18.

### Envelope Protein

**Fig. 19:**
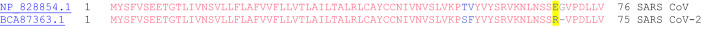
The primary amino acid sequence of the E proteins (ORF4) from SARS CoV (NP_828854.1) and SARS CoV-2 (BCA87363.1). Sequence identity: 94.7%, sequence similarity: 97.4%

The envelope protein is a small integral membrane protein in coronaviruses, which can oligomerize and create an ion channel [49]. The four structural proteins of coronaviruses are: S protein, M protein, E protein, and N protein [50]. The E protein has been shown to play multiple roles in the viral replication cycle: [1] viral assembly, [51] [2] virion release, [52] and [3] viral pathogenesis [53]. Interestingly, in the sequence alignment of the E proteins from SARS CoV and SARS CoV-2 (Fig. 19), there is a glutamate residue (E69) with a negative charge in SARS CoV that corresponds to a positively charged arginine in SARS CoV-2 (R69).

### Membrane Protein

**Fig. 20:**
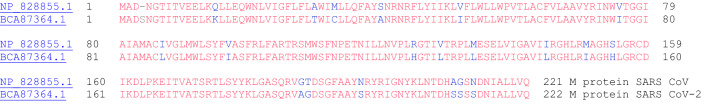
The primary amino acid sequence of the M proteins (ORF5) from SARS CoV (NP_828855.1) and SARS CoV-2 (BCA87364.1). Sequence identity: 90.5%, sequence similarity: 98.2%

The SARS coronavirus membrane (M) protein is an integral membrane protein that plays an important role in viral assembly [54]. In addition, the SARS coronavirus M protein has been shown to induce apoptosis [55]. The M protein interacts with the nucleocapsid (N) protein to encapsidate the RNA genome [56]. Figure 20 shows the amino acid sequence alignment of the two ORF5 proteins from SARS CoV and SARS CoV-2.

### ORF6 Protein

**Fig. 21:**
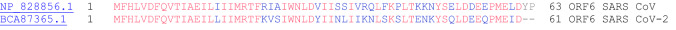
The primary amino acid sequence alignment of the ORF6 proteins from SARS CoV (NP_828856.1) and SARS CoV-2 (BCA87365.1). Sequence identity: 68.9%, sequence similarity: 93.4%

The ORF6 protein from SARS coronavirus is an accessory protein that plays an important role in viral pathogenesis [57, 58]. Using a yeast two-hybrid system, ORF6 was shown to interact with NSP8, the nonstructural protein related to promoting RNA polymerase activity [57]. Figure 21 shows the amino acid sequence alignment of the two ORF6 proteins from SARS CoV and SARS CoV-2.

### ORF7a Protein

**Fig. 22:**
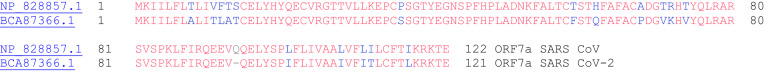
The primary amino acid sequence of the ORF7a protein from SARS CoV (NP_828857.1) and SARS CoV-2 (BCA87366.1). Sequence identity: 85.2%, sequence similarity: 95.9%

ORF7a from SARS coronavirus is an accessory protein that is a type I transmembrane protein and its crystal structure has been determined [59]. Figure 22 shows the amino acid sequence alignment between the two ORF7a proteins of SARS CoV and SARS CoV-2.

### ORF7b Protein

**Fig. 23:**
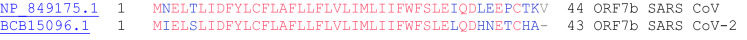
The primary amino acid sequence of the ORF7b proteins from SARS CoV (NP_849175.1) and SARS CoV-2 (BCB15096.1). Sequence identity: 85.4%, sequence similarity: 97.2%

The ORF7b accessory protein from SARS coronavirus is localized in the Golgi compartment [60]. Figure 23 shows the sequence alignment between the two ORF7b proteins of SARS CoV and SARS CoV-2.

### ORF8 Protein

**Fig. 24:**
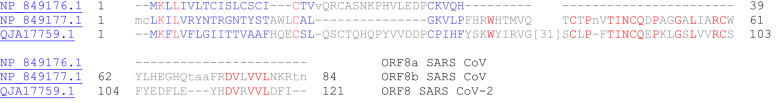
Sequence alignment of ORF8a (NP_849176.1) and ORF8b (NP_849177.1) proteins from SARS CoV (top and middle) with the ORF8 protein (QJA17759.1) from SARS CoV-2 (bottom). Sequence identity and sequence similarity between ORF8a (SARS CoV) and ORF8 (SARS CoV-2): 31.7% and 70.7% in 41 amino acid overlap. Sequence identity and sequence similarity between ORF8b (SARS CoV) and ORF8 (SARS CoV-2): 40.5% and 66.7% in 42 amino acid overlap

SARS CoV-2 has a single ORF8 protein while SARS CoV has two ORF8 proteins: ORF8a and ORF8b [61]. In SARS CoV, the ORF8b protein binds to the IRF association domain (IAD) region of interferon regulatory factor 3 (IRF3), which in turn inactivates interferon signaling [62]. Interestingly, L84S and S62L missense mutations have been reported in various SARS CoV-2 sequences [5]. Figure 24 shows the alignment between the ORF8 protein of SARS CoV-2 with the ORF8a and ORF8b proteins of SARS CoV.

### Nucleocapsid Protein

**Fig. 25:**
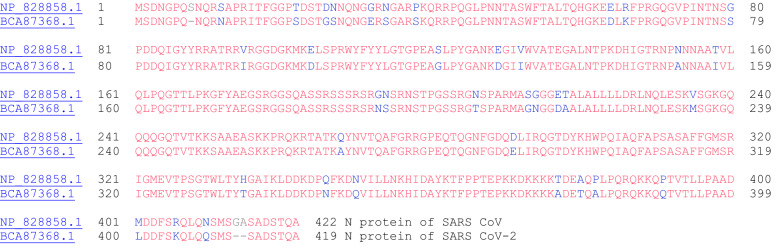
The primary amino acid sequence of the N protein from SARS CoV (ORF9a, NP_828858.1) and SARS CoV-2 (ORF9, BCA87368.1). Sequence identity: 90.5%, sequence similarity: 97.2%

The nucleocapsid (N) protein of coronaviruses is a structural protein that binds directly to viral RNA and providing stability [63]. Furthermore, the N protein of SARS CoV-2 (Fig. 24) has been found to antagonize antiviral RNAi [64]. In another study, the nucleocapsid protein of SARS CoV was found to inhibit the activity of cyclin-cyclin-dependent kinase (cyclin-CDK) complex. Inactivation of the cyclin-CDK complex results in hypophosphorylation of the retinoblastoma protein and in turn inhibits S phase (genome replication) progression in the cell cycle [65]. Figure 25 shows the amino acid sequence alignment between the two N proteins of SARS CoV and SARS CoV-2.

### ORF10 Protein

**Fig. 26:**
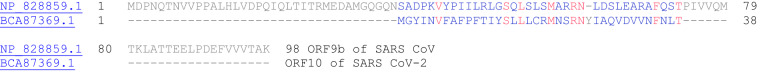
The primary amino acid sequence alignment of the ORF9b protein from SARS CoV and the ORF10 protein from SARS CoV-2 (Accession number: BCA87369.1). Sequence identity: 28.6%, sequence similarity: 52.4%

ORF10 protein from SARS CoV-2 is comprised of 38-amino acids and its function is unknown. Interestingly, SARS CoV possesses an ORF9b protein (NP_828859.1), which is not present in SARS CoV-2. Figure 26 shows the sequence alignment between ORF10 of SARS CoV-2 with ORF9b of SARS CoV. SARS CoV-2 does not have an ORF10 protein. A summary of the sequence identities and similarities of the discussed proteins from SARS CoV and SARS CoV-2 is shown in Table 4.

**Table 4:** Sequence identity and similarities between SARS CoV-2 proteins and SARS CoV proteins determined through LALIGN (17) (see Supporting Information)

Entry	Protein	Amino acid overlap	Sequence identity	Sequence similarity
1	NSP1	180	84.4%	93.4%
2	NSP2	638	68.3%	90.0%
3	NSP3	1,952	76.0%	91.8%
4	NSP4	500	80.0%	95.0%
5	NSP5	306	96.1%	99.7%
6	NSP6	287	88.2%	98.3%
7	NSP7	83	98.8%	100.0%
8	NSP8	198	97.5%	100.0%
9	NSP9	113	97.3%	99.1%
10	NSP10	139	97.1%	99.3%
11	NSP11	13	84.6%	100.0%
12	NSP12	932	96.4%	99.4%
13	NSP13	601	99.8%	100.0%
14	NSP14	527	95.1%	99.1%
15	NSP15	346	88.7%	97.7%
16	NSP16	298	93.3%	99.0%
17	S protein	1,277	76.0%	91.5%
18	ORF3a	1,381	72.4%	90.2%
19	E Protein	76	94.7%	97.4%
20	M Protein	222	90.5%	98.2%
21	ORF6	61	68.9%	93.4%
22	ORF7a	122	85.2%	95.9%
23	ORF7b	41	85.4%	92.7%
24a	(ORF8 vs 8a)^a^	41	31.7%	70.7%
24b	(ORF8 vs 8b)^a^	42	40.5%	66.7%
25	N Protein	422	90.5%	97.2%
26	(ORF10 vs 9b)^a^	21	28.6%	52.4%

^a^(SARS CoV-2 protein vs SARS CoV protein). Other reports have also reported amino acid sequence identities using different algorithms (3,67)

## Overlapping Genes: ORF9b and Two Proteins with Variation Among SARS CoV-2 Sequences: ORF3b and ORF9c

Overlapping genes in coronavirus have been previously observed [67]. For example, in SARS CoV, the start and end positions in the nucleotide sequence of the N-protein are 28,120 and 29,388 respectively while the ORF9b gene of SARS CoV starts and ends at positions: 28,130 and 28,426 (within the gene sequence of the N-protein) [68]. Similarly, there is a putative ORF9b protein in SARS CoV-2 located within the gene encoding the N-protein, which does not yet have an accession number [4].

In the gene alignment of 2,784 SARS CoV-2 sequences, two variations were recognized in the SARS CoV-2 genome [66]. It was recognized that a premature stop codon at position 14 of ORF3b in SARS CoV-2 in 17.6% of isolates (position E14). Furthermore, there were two mutations that gave rise to premature stop codons in ORF9c (at position Q41 in 0.7% of sequences and at position Q44 in 1.4% of the sequences). The observations of these stop codons suggested that these genes for ORF3b and ORF9c may not be bonafide gene sequences in SARS CoV-2. With the putative SARS CoV-2 ORF3b protein, only 12 out of 57 overlapping amino acid residues were identical (21% sequence identity) to the ORF3b protein of SARS CoV [3]. In the above sections, ORF3b and ORF9c for SARS CoV-2 were not included in the above analysis. Another protein lacking an accession number is ORF14 [69].

## Nontranslated (or Untranslated) Regions of SARS CoV-2 Genome

Considering the locations of each gene presented in Table 1, there are regions of the genome that are not translated into proteins, which is related to the non-canonical translational strategy employed by this virus [70]. The nucleotide sequences between the genes are the intergenic regions [71]. For instance, there is a conserved transctiption regulatory sequence (TRS) – a conserved hexanucleotide sequence: (5′-ACGAAC-3′) [71] that could be found in between some of the open reading frames (Table 5, Entries 2, 3, 4, 5, 7, and 9). This particular sequence has previously been identified as the leader-body fusion sites [71]. Furthermore, this sequence is a conserved motif that can be found in subgroup 2b, 2c, and 2d viruses [72]. Another transcriptional regulatory sequence was CUAAAC (e.g. Table 5, Entry 1) [73, 74].

**Table 5:** Nontranslated RNA sequence of SARS CoV-2 (NCBI Reference Sequence: NC_045512.2)

Entry	Location (position)	Sequence
1	Beginning-ORF1ab (1–265)	1 auuaaagguu uauaccuucc cagguaacaa accaaccaac uuucgaucuc uuguagaucu
		61 guucucuaaa cgaacuuuaa aaucugugug gcugucacuc ggcugcaugc uuagugcacu
		121 cacgcaguau aauuaauaac uaauuacugu cguugacagg acacgaguaa cucgucuauc
		181 uucugcaggc ugcuuacggu uucguccgug uugcagccga ucaucagcac aucuagguuu
		241 cguccgggug ugaccgaaag guaag
2	ORF1ab-ORF2 (21,556–21,562)	1 acgaaca
3	ORF2-ORF3a (25,385–25,392)	1 acgaacuu
4	ORF3a-ORF4 (26,221–26,244)	1 gcacaagcug augaguacga acu
5	ORF4-ORF5 (26,473–26,522)	1 acgaacuaaa uauuauauua guuuuucugu uuggaacuuu aauuuuagcc
6	ORF5-ORF6 (27,192–27,201)	1 gugacaacag
7	ORF6-ORF7a (27,388–27,393)	1 acgaac
8	ORF7b-ORF8 (27,888–27,893)	1 acgaac
9	ORF8-ORF9 (28,260–28,273)	1 acgaacaaac uaaa
10	ORF9-ORF10 (29,534–29,557)	1 acucaugcag accacacaag gcag
11	ORF10-end (29,675–29,903)	1 caaucuuuaa ucagugugua acauuaggga ggacuugaaa gagccaccac auuuucaccg
		61 aggccacgcg gaguacgauc gaguguacag ugaacaaugc uagggagagc ugccuauaug
		121 gaagagcccu aauguguaaa auuaauuuua guagugcuau ccccauguga uuuuaauagc
		181 uucuuaggag aaugacaaaa aaaaaaaaaa aaaaaaaaaa aaaaaaaaa

## Exploration of Treatment Options for COVID-19

An intense effort has been put forth to discover potential treatment options for COVID-19, the disease caused by SARS CoV-2 [75–77]. For instance, the FDA approved drug, ivermectin, is known to inhibit nuclear transport, and has been shown to inhibit the replication of SARS CoV-2 [78]. Other drugs have been repurposed and tested against COVID-19 [79, 80]. Remdesivir is a potential antiviral drug originally developed to treat ebola [81] and has been used to treat COVID-19 [82] by inhibiting viral RNA polymerase activity. Hydroxychloroquine [83] and chloroquine [84] have been used to potentially treat COVID-19. However, the use of these drugs has been known to result in cardiotoxicity [85, 86]. In fact, in a recent observational study, it was determined that hydroxychloroquine administration was not associated with a greatly lowered risk of death from COVID-19 [87].

A recent study identified 332 human proteins that interact with SARS CoV-2 proteins [66]. In this report, the predicted SARS CoV-2 proteins (NSPs 1–16 and ORFs) were expressed with 2xstreptavidin affinity tags. These tagged SARS CoV-2 proteins were expressed in human embryonic kidney (HEK)293T/17 cells and isolated the viral protein-(human protein) interactions using affinity purification-mass spectrometry. A total 332 protein–protein interactions (PPIs between SARS CoV-2 proteins and human proteins) were identified. Of these PPIs, 66 of them are targetable by compounds. Table 6 shows a set of compounds that target the identified PPIs based on chemoinformatics (entries 1–28) or expertise knowledge (entries 29–44). From the subset of potential antiviral compounds that were tested, two classes of compounds were found to be effective against viral pathogenesis: [1] protein translation inhibitors (i.e. zotatifin, ternatin-4, and PS3061), and [2] Sigma1 and Sigma2 receptor ligands (i.e. approved drugs: clemastine, cloperastine, and progesterone and PB28, which was ~ 20 times more potent than hydroxychloroquine with an IC_90_ of 280 nM in the viral titer assay is undergoing pre-clinical trials for anti-cancer [88] activity).

**Table 6:** Drugs that potentially target (modulate) proteins that interact with SARS CoV-2 proteins as described in reference [66]

Entry	Viral Protein-(Human Gene)	Compound Name(s)
1	E protein-(BRD2/4)	JQ1,^a^ RVX-208^b^
2	N protein-(CSNK2A2)	Silmitasertib (cancer),^c^ TMCB^a^
3	NSP5-(HDAC2)	Apicidin,^a^ Valproic acid (CNS disease, cancer)^c^
4	NSP6-(ATP6AP1)	Bafilomycin A1^a^
5	NSP6-(SIGMAR1)	E-52862,^b^ PD-144418,^a^ RS-PPCC,^a^ PB28,^a^
		Haloperidol (CNS disease)^c^
6	NSP6-(SLC6A15)	Loratadine (antihistamine)^3^
7	ORF9C-(TMEM97)	PB28,^a^ haloperidol (CNS disease)^c^
8	M protein-(ATP6V1A)	Bafilomycin A1^a^
9	NSP7-(COMT)	Entacapone (Parkinson’s disease)^c^
10	NSP7-(PTGES2)	Indomethacin (inflammation/pain)^c^
11	NSP7-(NDUFs)	Metformin (diabetes)^c^
12	ORF9C-(NDUFs)	Metformin^c^
13	NSP12-(RIPK1)	Ponatinib (cancer)^c^
14	NSP13-(PRKACA)	H-89^a^
15	NSP14-(IMPDH2)	Merimepodib^b^
16	NSP14-(GLA)	Migalastat (Fabry disease)^c^
17	NSP14-(IMPDH2)	Mycophenolic acid (organ rejection),^3^ ribavirin (virus)^c^
18	ORF8-(DNMT1)	Azacitidine^c^
19	ORF8-(LOX)	CCT 365623^a^
20	ORF9b-(MARK2/3)	Midostaurin,^3^ Ruxolitinib^c^
21	ORF9b-(DCTPP1)	ZINC1775962367,^a^ ZINC4326719,^a^ ZINC4511851^a^
22	ORF9b/NSP13-(MARK3/TBK1)	ZINC95559591^a^
23	ORF9C-(F2RL1)	AC-55541,^a^ AZ8838^a^
24	ORF9C-(ABCC1)	Daunorubicin^c^
25	ORF9C-(F2RL1)	GB110^a^
26	ORF9C-(ABCC1)	S-Verapamil (hypertension)^c^
27	ORF9C-(F2RL1)	AZ3451^a^
28	M-Protein-(SLC1A3)	UCPH-101^a^
29	E protein-(BRD2/4)	ABBV-744,^b^ dBET6,^a^ MZ1,^a^ CPI-0610^b^
30	N protein-(LARP1)	Sapanisertib,^b^ Rapamycin (organ rejection)^c^
31	NSP2-(FKBP15)	Rapamycin^c^
32	ORF8-(FKBP7/10)	Rapamycin^c^
33	NSP2-(EIF4E2/H)	Zotatifin^b^
34	ORF10-(VCP)	CB5083^b^
35	NSP6-(SIGMAR1)	Chloroquine (malaria)^c^
36	NSP9-NEK9	Dabrafenaib (cancer)^c^
37	NSP13-CEP250	WDB002^b^
38	NSP14-IMPDH2	Sanglifehrin A^a^
39	ORF8-(FKBP7)	FK-506 (organ rejection)^c^
40	ORF8-(FKBP10)	FK-506^c^
41	ORF10-(CUL2)	Pevonedistat^b^
42	ORF10-(VCP)	DBeQ, ML240^a^
43	ORF8-(PLOD1/2)	Minoxidil (hair loss)^c^
44	NSP4/9/ORF6-(NUPs RAE1)	Selinexor (cancer)^c^

Entries 1–28 were determined from chemoinformatics. Entries 29–44 were determined from specialist knowledge

^a^Pre-clinical

^b^Clinical trial

^c^FDA-approved drug. In parentheses after the drug is what the FDA-approved drug is used to treat in the clinic

Moreover, in another collaborative study, a library of 12,000 FDA-approved or clinical-stage drugs were tested against SARS CoV-2 infection in Vero-E6 (African green monkey kidney) cells [89]. Some effective compounds identified in the screen were: PIKfyve kinase inhibitor Apilimod, cysteine protease inhibitors (MDL-28170, Z LVG CHN2, VBY-825, and ONO 5334), and MLN-3897 (a CCR1 antagonist).

Traditional Chinese Medicine (TCM) has also been employed in China to treat COVID-19 [90]. However, due to potential toxic components present in TCM remedies, [91, 92] the use of this strategy should be handled with caution [93]. Ironically, it has been suggested that TCM could have potentially been the cause of COVID-19 [94].

In addition to small molecules, vaccines are also currently being developed against SARS CoV-2, [95] and convalescent plasma transfusions have been used to treat COVID-19 [96]. Nevertheless, more research is needed to develop effective treatments against SARS CoV-2 especially in the context of future outbreaks [97, 98].

## Conclusion

Although there is some variation in sequence in the proteins, many of the proteins found in SARS CoV-2 (NC_045512.2) are also found in SARS CoV (AY515512.1 or NC_004718.3) with 77.1% of the protein sequences shared in their proteomes [99]. Thus, previous research on related coronavirus proteins enable a better understanding of how we can approach to understand the current coronavirus (SARS CoV-2) that caused the current global pandemic (COVID-19). The general structures of most of the proteins from SARS CoV-2 can be visualized from homology models [100]. Advances in the knowledge of the structures and functions of the proteins in SARS CoV-2 will enable researchers to design better antiviral drugs that target this virus.

## Electronic supplementary material

Below is the link to the electronic supplementary material.

Supplementary file1 (PDF 329 kb)
