# Supplementary material for: The Proteins of Severe Acute Respiratory Syndrome Coronavirus-2 (SARS CoV-2 or n-COV19), the Cause of COVID-19
Source: Protein J. 2020 May 23;39(3):198–216. doi: 10.1007/s10930-020-09901-4 (PMC7245191; doi:10.1007/s10930-020-09901-4)
Supplement: Supplementary file 1 — Supplementary file1 (PDF 329 kb) [file 10930_2020_9901_MOESM1_ESM.pdf]

The Proteins of Severe Acute Respiratory Syndrome Coronavirus-2 (SARS CoV-2 or n-COV19), the Cause of COVID-19

Supporting Information

Francis K. Yoshimoto*

(ORC ID: 0000-0002-2308-2999)

Department of Chemistry, The University of Texas at San Antonio (UTSA), San Antonio, TX 78249-0698

francis.yoshimoto@utsa.edu

The Protein Journal, 2020, XXXX, XXXXX

Sequence alignment results presented in Table 4.
(also for ORF3b and ORF14 sequences)

Readseq version 2.1.30 (12-May-2010) Readseq version 2.1.30 (12-May-2010)

lalign output for NSP1 SARS COV vs. NSP1 SARS COV2

[EMBnet-Server] Date: Fri May 8 9:18:07 2020

```
# bin/lalign36 -E 10.0 -f -12 -g -2 21613.1.seq 21613.2.seq -J -K 3
LALIGN finds non-overlapping local alignments
 version 36.3.5e Nov, 2012(preload8)
Please cite:
 X. Huang and W. Miller (1991) Adv. Appl. Math. 12:373-381

Query: 21613.1.seq
  1>>>NSP1 SARS CoV 180 bp - 180 aa
Library: 21613.2.seq
      180 residues in     1 sequences

Statistics: (shuffled [500]) MLE statistics: Lambda= 0.1775;  K=0.04078
 statistics sampled from 1 (1) to 500 sequences
Threshold: E() < 10 score: 28
Algorithm: Smith-Waterman (SSE2, Michael Farrar 2006) (7.2 Nov 2010)
Parameters: BL50 matrix (15:-5), open/ext: -12/-2
 Scan time:  0.010

>>NSP1 SARS CoV-2 180 bp                                  (180 aa)
 Waterman-Eggert score: 1023;  266.6 bits; E(1) <  1.8e-76
84.4% identity (93.9% similar) in 180 aa overlap (1-180:1-180)

               10        20        30        40        50        60
NSP1   MESLVLGVNEKTHVQLSLPVLQVRDVLVRGFGDSVEEALSEAREHLKNGTCGLVELEKGV
       ::::: : :::::::::::::::::::::::::::::.:::::.:::.:::::::.::::
NSP1   MESLVPGFNEKTHVQLSLPVLQVRDVLVRGFGDSVEEVLSEARQHLKDGTCGLVEVEKGV
               10        20        30        40        50        60

               70        80        90       100       110       120
NSP1   LPQLEQPYVFIKRSDALSTNHGHKVVELVAEMDGIQYGRSGITLGVLVPHVGETPIAYRN
       :::::::::::::::: .. ::: .::::::.::::::::: ::::::::::: :.:::.
NSP1   LPQLEQPYVFIKRSDARTAPHGHVMVELVAELEGIQYGRSGETLGVLVPHVGEIPVAYRK
               70        80        90       100       110       120

              130       140       150       160       170       180
NSP1   VLLRKNGNKGAGGHSYGIDLKSYDLGDELGTDPIEDYEQNWNTKHGSGALRELTRELNGG
       ::::::::::::::::: ::::.:::::::::: ::...::::::.::. ::: ::::::
NSP1   VLLRKNGNKGAGGHSYGADLKSFDLGDELGTDPYEDFQENWNTKHSSGVTRELMRELNGG
              130       140       150       160       170       180

>--
 Waterman-Eggert score: 42;  15.4 bits; E(1) <  0.53
25.7% identity (55.4% similar) in 74 aa overlap (63-134:15-83)

             70        80        90       100       110        120
NSP1   QLEQPYVFIKRSDALSTNHGHKVVELVAEMDGIQYGRSGITLGVLVPHVGETP-IAYRNV
       ::  : . ..  :.:  . : .: :...:    :. ..: : :..  . :  : .    :
NSP1   QLSLPVLQVR--DVLVRGFGDSVEEVLSEAR--QHLKDG-TCGLVEVEKGVLPQLEQPYV
```

```
           20          30        40           50        60

             130
NSP1   LLRKNGNKGAG-GH
       .....  . :  ::
NSP1   FIKRSDARTAPHGH
      70        80

>--
 Waterman-Eggert score: 33;  13.1 bits; E(1) <  0.98
38.9% identity (77.8% similar) in 18 aa overlap (78-94:162-179)

        80         90
NSP1   STNHGHKVV-ELVAEMDG
       .:.:.  :. ::. :..:
NSP1   NTKHSSGVTRELMRELNG
             170



180 residues in 1 query   sequences
180 residues in 1 library sequences
 Scomplib [36.3.5e Nov, 2012(preload8)]
 start: Fri May  8 09:18:07 2020 done: Fri May  8 09:18:07 2020
 Total Scan time:  0.010 Total Display time:  0.000

Function used was LALIGN [36.3.5e Nov, 2012(preload8)]
```

LALIGN

Home | Contact
Readseq version 2.1.30 (12-May-2010) Readseq version 2.1.30 (12-May-2010)

lalign output for NSP2 SARS COV vs. NSP2 SARS COV2

[EMBnet-Server] Date: Fri May 8 8:16:55 2020

```
# bin/lalign36 -E 10.0 -f -12 -g -2 17255.1.seq 17255.2.seq -J -K 3
LALIGN finds non-overlapping local alignments
 version 36.3.5e Nov, 2012(preload8)
Please cite:
 X. Huang and W. Miller (1991) Adv. Appl. Math. 12:373-381

Query: 17255.1.seq
  1>>>NSP2 SARS CoV 638 bp - 638 aa
Library: 17255.2.seq
      638 residues in     1 sequences

Statistics: (shuffled [500]) MLE statistics: Lambda= 0.1778;  K=0.02289
 statistics sampled from 1 (1) to 500 sequences
Threshold: E() < 10 score: 38
Algorithm: Smith-Waterman (SSE2, Michael Farrar 2006) (7.2 Nov 2010)
Parameters: BL50 matrix (15:-5), open/ext: -12/-2
 Scan time:  0.080

>>NSP2 SARS CoV-2 638 bp                                 (638 aa)
 Waterman-Eggert score: 3094;  798.9 bits; E(1) <  0
68.3% identity (90.0% similar) in 638 aa overlap (1-638:1-638)

               10        20        30        40        50        60
NSP2   AVTRYVDNNFCGPDGYPLDCIKDFLARAGKSMCTLSEQLDYIESKRGVYCCRDHEHEIAW
       : ::::::::::::::::.::::.::::::. ::::::::.:..::::::::.:::::::
NSP2   AYTRYVDNNFCGPDGYPLECIKDLLARAGKASCTLSEQLDFIDTKRGVYCCREHEHEIAW
               10        20        30        40        50        60

               70        80        90       100       110       120
NSP2   FTERSDKSYEHQTPFEIKSAKKFDTFKGECPKFVFPLNSKVKVIQPRVEKKKTEGFMGRI
       .::::.:::: ::::::: :::::::.::::.::::::: .:.::::::::: .::::::
NSP2   YTERSEKSYELQTPFEIKLAKKFDTFNGECPNFVFPLNSIIKTIQPRVEKKKLDGFMGRI
               70        80        90       100       110       120

              130       140       150       160       170       180
NSP2   RSVYPVASPQECNNMHLSTLMKCNHCDEVSWQTCDFLKATCEHCGTENLVIEGPTTCGYL
       :::::::::.:::.: :::::::.:: :.:::: ::.::::: ::::::. :: ::::::
NSP2   RSVYPVASPNECNQMCLSTLMKCDHCGETSWQTGDFVKATCEFCGTENLTKEGATTCGYL
              130       140       150       160       170       180

              190       200       210       220       230       240
NSP2   PTNAVVKMPCPACQDPEIGPEHSVADYHNHSNIETRLRKGGRTRCFGGCVFAYVGCYNKR
       : :::::. ::::.. :.::::::.:.:::.:...: :::::::  ::::::.::::.::
```

```
NSP2   PQNAVVKIYCPACHNSEVGPEHSLAEYHNESGLKTILRKGGRTIAFGGCVFSYVGCHNKC
              190       200       210       220       230       240

              250       260       270       280       290       300
NSP2   AYWVPRASADIGSGHTGITGDNVETLNEDLLEILSRERVNINIVGDFHLNEEVAIILASF
       :::::::::.:: .:::..:.. : ::..:::::..:.:::::::::.::::.:::::::
NSP2   AYWVPRASANIGCNHTGVVGEGSEGLNDNLLEILQKEKVNINIVGDFKLNEEIAIILASF
              250       260       270       280       290       300

              310       320       330       340       350       360
NSP2   SASTSAFIDTIKSLDYKSFKTIVESCGNYKVTKGKPVKGAWNIGQQRSVLTPLCGFPSQA
       :::::::..:.:.::::.:: :::::::.::::::  :::::::.:.:.:.:: .: :.:
NSP2   SASTSAFVETVKGLDYKAFKQIVESCGNFKVTKGKAKKGAWNIGEQKSILSPLYAFASEA
              310       320       330       340       350       360

              370       380       390       400       410       420
NSP2   AGVIRSIFARTLDAANHSIPDLQRAAVTILDGISEQSLRLVDAMVYTSDLLTNSVIIMAY
       : :.::::.:::..:..:.  ::.::.:::::::. ::::.:::..:::: ::....:::
NSP2   ARVVRSIFSRTLETAQNSVRVLQKAAITILDGISQYSLRLIDAMMFTSDLATNNLVVMAY
              370       380       390       400       410       420

              430       440       450       460       470       480
NSP2   VTGGLVQQTSQWLSNLLGTTVEKLRPIFEWIEAKLSAGVEFLKDAWEILKFLITGVFDIV
       .:::.:: :::::.:..::. :::.:...:.: :.. :::::.:.:::.::. : . .::
NSP2   ITGGVVQLTSQWLTNIFGTVYEKLKPVLDWLEEKFKEGVEFLRDGWEIVKFISTCACEIV
              430       440       450       460       470       480

              490       500       510       520       530       540
NSP2   KGQIQVASDNIKDCVKCFIDVVNKALEMCIDQVTIAGAKLRSLNLGEVFIAQSKGLYRQC
        ::: . . .::. :. :. .::: : .: :.. :.::::..:::::.:...::::::.:
NSP2   GGQIVTCAKEIKESVQTFFKLVNKFLALCADSIIIGGAKLKALNLGETFVTHSKGLYRKC
              490       500       510       520       530       540

              550       560       570       580       590       600
NSP2   IRGKEQLQLLMPLKAPKEVTFLEGDSHDTVLTSEEVVLKNGELEALETPVDSFTNGAIVG
       ....:.  ::::::::::. ::::..  : . .::::::.:.:. :: :..  ... .::
NSP2   VKSREETGLLMPLKAPKEIIFLEGETLPTEVLTEEVVLKTGDLQPLEQPTSEAVEAPLVG
              550       560       570       580       590       600

              610       620       630
NSP2   TPVCVNGLMLLEIKDKEQYCALSPGLLATNNVFRLKGG
       ::::.:::::::::: :.::::.:.....:::.: ::::
NSP2   TPVCINGLMLLEIKDTEKYCALAPNMMVTNNTFTLKGG
              610       620       630

>--
 Waterman-Eggert score: 59;  20.6 bits; E(1) <  0.23
25.0% identity (44.2% similar) in 52 aa overlap (144-193:130-177)

           150       160       170         180       190
NSP2   NHCDEVSWQTCDFLKATCEHCGTENLVIEG--PTTCGYLPTNAVVKMPCPAC
       :.:.    : :      :.:::  .        .:: .  :. ..:    .:
NSP2   NECN----QMCLSTLMKCDHCGETSWQTGDFVKATCEFCGTENLTKEGATTC
     130           140       150       160       170

>--
 Waterman-Eggert score: 47;  17.5 bits; E(1) <  0.89
```

```
22.2% identity (50.0% similar) in 54 aa overlap (20-73:164-211)

      20        30        40        50        60        70
NSP2   CIKDFLARAGKSMCTLSEQLDYIESKRGVYCCRDHEHEIAWFTERSDKSYEHQT
       :  . :.. : . :    :   ..    .::   :. :..   :.:   :....
NSP2   CGTENLTKEGATTCGYLPQNAVVK----IYCPACHNSEVG--PEHSLAEYHNES
           170       180           190         200       210



638 residues in 1 query   sequences
638 residues in 1 library sequences
 Scomplib [36.3.5e Nov, 2012(preload8)]
 start: Fri May  8 08:16:56 2020 done: Fri May  8 08:16:56 2020
 Total Scan time:  0.080 Total Display time:  0.040

Function used was LALIGN [36.3.5e Nov, 2012(preload8)]
```

SIB Swiss Institute of Bioinformatics | Contact
Back to the Top

lalign output for NSP3 SARS COV vs. NSP3 SARS COV2

[EMBnet-Server] Date: Fri May 8 23:53:10 2020

```
# bin/lalign36 -E 10.0 -f -12 -g -2 5100.1.seq 5100.2.seq -J -K 3
LALIGN finds non-overlapping local alignments
 version 36.3.5e Nov, 2012(preload8)
Please cite:
 X. Huang and W. Miller (1991) Adv. Appl. Math. 12:373-381

Query: 5100.1.seq
  1>>>NSP3 SARS CoV 1922 bp - 1922 aa
Library: 5100.2.seq
     1945 residues in     1 sequences

Statistics: (shuffled [500]) MLE statistics: Lambda= 0.1707;  K=0.01513
 statistics sampled from 1 (1) to 500 sequences
Threshold: E() < 10 score: 51
Algorithm: Smith-Waterman (SSE2, Michael Farrar 2006) (7.2 Nov 2010)
Parameters: BL50 matrix (15:-5), open/ext: -12/-2
 Scan time:  0.400

>>NSP3 SARS CoV-2 1945 bp                           (1945 aa)
 Waterman-Eggert score: 10007;  2470.4 bits; E(1) <  0
76.0% identity (91.8% similar) in 1952 aa overlap (1-1922:1-1945)

               10        20        30        40        50        60
NSP3   APIKGVTFGEDTVWEVQGYKNVRITFELDERVDKVLNEKCSVYTVESGTEVTEFACVVAE
       :: : ::::.::: ::::::.: ::::::::.:::::::::.:::: ::::.:::::::.
NSP3   APTK-VTFGDDTVIEVQGYKSVNITFELDERIDKVLNEKCSAYTVELGTEVNEFACVVAD
                10        20        30        40        50

               70        80        90       100       110       120
NSP3   AVVKTLQPVSDLLTNMGIDLDEWSVATFYLFDDAGEENFSSRMYCSFYPPDEEEEDDAEC
       ::.:::::::.::: .::::::::.::.::::..:: ...:.::::::::::.::. ..:
NSP3   AVIKTLQPVSELLTPLGIDLDEWSMATYYLFDESGEFKLASHMYCSFYPPDEDEEE-GDC
      60        70        80        90       100       110

              130       140       150       160       170
NSP3   EEEEIDETCEHEYGTEDDYQGLPLEFGASAETVRVEEEEEEDWLDDTTEQS---------
       ::::.. . ..:::::::::: ::::::.. ... :::.::::::: ..:.
NSP3   EEEEFEPSTQYEYGTEDDYQGKPLEFGATSAALQPEEEQEEDWLDDDSQQTVGQQDGSED
     120       130       140       150       160       170

                         180          190       200       210
NSP3   ----------EIEP--EPEPTP---EEPVNQFTGYLKLTDNVAIKCVDIVKEAQSANPMV
                 :..:  : : ::      ::.:.::::::::: :: .:::.::....: :
NSP3   NQTTTIQTIVEVQPQLEMELTPVVQTIEVNSFSGYLKLTDNVYIKNADIVEEAKKVKPTV
     180       190       200       210       220       230

        220       230       240       250       260       270
NSP3   IVNAANIHLKHGGGVAGALNKATNGAMQKESDDYIKLNGPLTVGGSCLLSGHNLAKKCLH
       .:::::..::::::::::::::::.::: ::::::  :::: :::::.::::::::.:::
NSP3   VVNAANVYLKHGGGVAGALNKATNNAMQVESDDYIATNGPLKVGGSCVLSGHNLAKHCLH
```

```
      240       250       260       270       280       290

        280       290       300       310       320       330
NSP3   VVGPNLNAGEDIQLLKAAYENFNSQDILLAPLLSAGIFGAKPLQSLQVCVQTVRTQVYIA
       :::::.: ::::::::.::::::....::::::::::::: :..::.:::.::::.::.:
NSP3   VVGPNVNKGEDIQLLKSAYENFNQHEVLLAPLLSAGIFGADPIHSLRVCVDTVRTNVYLA
      300       310       320       330       340       350

        340       350             360       370       380       390
NSP3   VNDKALYEQVVMDYLDNLKPR------VEAPKQEEPPNTEDSKTEEKSVVQKPVDVKPKI
       : :: ::...: ..:.  . .      .: ::.:  :   .::    :: :.  : : ::
NSP3   VFDKNLYDKLVSSFLEMKSEKQVEQKIAEIPKEEVKPFITESKP---SVEQRKQDDK-KI
      360       370       380       390       400          410

              400       410       420       430       440       450
NSP3   KACIDEVTTTLEETKFLTNKLLLFADINGKLYHDSQNMLRGEDMSFLEKDAPYMVGDVIT
       :::..:::::::::::::..:::. ::::.:. :: ...   :..::.:::::.::::.
NSP3   KACVEEVTTTLEETKFLTENLLLYIDINGNLHPDSATLVSDIDITFLKKDAPYIVGDVVQ
          420       430       440       450       460       470

              460       470       480       490       500       510
NSP3   SGDITCVVIPSKKAGGTTEMLSRALKKVPVDEYITTYPGQGCAGYTLEEAKTALKKCKSA
        : .: ::::.::::::::::..::.:::.:.:::::::::  :::.:::::.:::::::
NSP3   EGVLTAVVIPTKKAGGTTEMLAKALRKVPTDNYITTYPGQGLNGYTVEEAKTVLKKCKSA
          480       490       500       510       520       530

              520       530       540       550       560       570
NSP3   FYVLPSEAPNAKEEILGTVSWNLREMLAHAEETRKLMPICMDVRAIMATIQRKYKGIKIQ
       ::.:::   : :.:::::::::::::::::::::::::.:....::..::::::::::::
NSP3   FYILPSIISNEKQEILGTVSWNLREMLAHAEETRKLMPVCVETKAIVSTIQRKYKGIKIQ
          540       550       560       570       580       590

              580       590       600       610       620       630
NSP3   EGIVDYGVRFFFYTSKEPVASIITKLNSLNEPLVTMPIGYVTHGFNLEEAARCMRSLKAP
       ::.::::.::.:::::  :::.:. ::.::: :::::.::::::.::::::: :::::.:
NSP3   EGVVDYGARFYFYTSKTTVASLINTLNDLNETLVTMPLGYVTHGLNLEEAARYMRSLKVP
          600       610       620       630       640       650

              640       650       660       670       680       690
NSP3   AVVSVSSPDAVTTYNGYLTSSSKTSEEHFVETVSLAGSYRDWSYSGQRTELGVEFLKRGD
       :.::::::::::.::::::::::: ::::.::.::::::.::::::: :.::.:::::::
NSP3   ATVSVSSPDAVTAYNGYLTSSSKTPEEHFIETISLAGSYKDWSYSGQSTQLGIEFLKRGD
          660       670       680       690       700       710

              700       710       720       730       740       750
NSP3   KIVYHTLESPVEFHLDGEVLSLDKLKSLLSLREVKTIKVFTTVDNTNLHTQLVDMSMTYG
       : ::.:  .:. :::::::...:.::.:::::::.:::::::::: :::::.::::::::
NSP3   KSVYYT-SNPTTFHLDGEVITFDNLKTLLSLREVRTIKVFTTVDNINLHTQVVDMSMTYG
          720        730       740       750       760       770

              760       770       780       790       800       810
NSP3   QQFGPTYLDGADVTKIKPHVNHEGKTFFVLPSDDTLRSEAFEYYHTLDESFLGRYMSALN
       ::::::::::::::::::: .::::::.:::.::::: :::::::: : :::::::::::
NSP3   QQFGPTYLDGADVTKIKPHNSHEGKTFYVLPNDDTLRVEAFEYYHTTDPSFLGRYMSALN
           780       790       800       810       820       830

              820       830       840       850       860       870
```

```
NSP3   HTKKWKFPQVGGLTSIKWADNNCYLSSVLLALQQLEVKFNAPALQEAYYRARAGDAANFC
       ::::::.:::.::::::::::::::...::.:::.:.::: ::::.::::::::.:::::
NSP3   HTKKWKYPQVNGLTSIKWADNNCYLATALLTLQQIELKFNPPALQDAYYRARAGEAANFC
           840       850       860       870       880       890

              880       890       900       910       920       930
NSP3   ALILAYSNKTVGELGDVRETMTHLLQHANLESAKRVLNVVCKHCGQKTTTLTGVEAVMYM
       :::::: ::::::::::::::..:.:::::.: ::::::::: :::. ::: ::::::::
NSP3   ALILAYCNKTVGELGDVRETMSYLFQHANLDSCKRVLNVVCKTCGQQQTTLKGVEAVMYM
           900       910       920       930       940       950

              940       950       960       970       980       990
NSP3   GTLSYDNLKTGVSIPCVCGRDATQYLVQQESSFVMMSAPPAEYKLQQGTFLCANEYTGNY
       :::::...: ::.:::.::..::.::::::: :::::::::.:.:..::: ::.::::::
NSP3   GTLSYEQFKKGVQIPCTCGKQATKYLVQQESPFVMMSAPPAQYELKHGTFTCASEYTGNY
           960       970       980       990      1000      1010

             1000      1010      1020      1030      1040      1050
NSP3   QCGHYTHITAKETLYRIDGAHLTKMSEYKGPVTDVFYKETSYTTTIKPVSYKLDGVTYTE
       ::::: :::.::::: :::: ::: ::::::.:::::::.:::::::::.::::::. ::
NSP3   QCGHYKHITSKETLYCIDGALLTKSSEYKGPITDVFYKENSYTTTIKPVTYKLDGVVCTE
          1020      1030      1040      1050      1060      1070

             1060      1070      1080      1090      1100      1110
NSP3   IEPKLDGYYKKDNAYYTEQPIDLVPTQPLPNASFDNFKLTCSNTKFADDLNQMTGFTKPA
       :.::::.::::::.:.:::::::::.:: :::::::::..:.: ::::::::.::. :::
NSP3   IDPKLDNYYKKDNSYFTEQPIDLVPNQPYPNASFDNFKFVCDNIKFADDLNQLTGYKKPA
          1080      1090      1100      1110      1120      1130

             1120      1130      1140      1150      1160      1170
NSP3   SRELSVTFFPDLNGDVVAIDYRHYSASFKKGAKLLHKPIVWHINQATTKTTFKPNTWCLR
       ::::.::::::::::::::::.::. ::::::::::::::::.:.::.:.:.::::::.:
NSP3   SRELKVTFFPDLNGDVVAIDYKHYTPSFKKGAKLLHKPIVWHVNNATNKATYKPNTWCIR
          1140      1150      1160      1170      1180      1190

             1180      1190      1200      1210      1220      1230
NSP3   CLWSTKPVDTSNSFEVLAVEDTQGMDNLACESQQPTSEEVVENPTIQKEVIECDVKTTEV
       ::::::::.:::::.::  ::.:::::::::. .:.::::::::::::.:.::.::::::
NSP3   CLWSTKPVETSNSFDVLKSEDAQGMDNLACEDLKPVSEEVVENPTIQKDVLECNVKTTEV
          1200      1210      1220      1230      1240      1250

             1240      1250      1260      1270      1280      1290
NSP3   VGNVILKPSDEGVKVTQELGHEDLMAAYVENTSITIKKPNELSLALGLKTIATHGIAAIN
       ::..::::.....:.:.:.:: :::::::.:.:.::::::::: .:::::.::::.::.:
NSP3   VGDIILKPANNSLKITEEVGHTDLMAAYVDNSSLTIKKPNELSRVLGLKTLATHGLAAVN
          1260      1270      1280      1290      1300      1310

             1300      1310      1320      1330      1340      1350
NSP3   SVPWSKILAYVKPFLGQAAITTSNCAKRLAQRVFNNYMPYVFTLLFQLCTFTKSTNSRIR
       ::::. :  :.::::.... ::.: . :  .:: .::::: ::::.::::::.::::::.
NSP3   SVPWDTIANYAKPFLNKVVSTTTNIVTRCLNRVCTNYMPYFFTLLLQLCTFTRSTNSRIK
          1320      1330      1340      1350      1360      1370

             1360      1370      1380      1390      1400      1410
NSP3   ASLPTTIAKNSVKSVAKLCLDAGINYVKSPKFSKLFTIAMWLLLLSICLGSLICVTAAFG
       ::.:::::::.::::.:.::.:.:..::.:::.::::..: .:.::::.::::::  :::.:
NSP3   ASMPTTIAKNTVKSVGKFCLEASFNYLKSPNFSKLINIIIWFLLLSVCLGSLIYSTAALG
```

```
          1380      1390      1400      1410      1420      1430

             1420      1430      1440      1450      1460      1470
NSP3   VLLSNFGAPSYCNGVRELYLNSSNVTTMDFCEGSFPCSICLSGLDSLDSYPALETIQVTI
       ::.::.: ::::.: :: ::::.:::   .: ::.:::.:::::::::.::.:::::.::
NSP3   VLMSNLGMPSYCTGYREGYLNSTNVTIATYCTGSIPCSVCLSGLDSLDTYPSLETIQITI
          1440      1450      1460      1470      1480      1490

             1480      1490      1500      1510      1520      1530
NSP3   SSYKLDLTILGLAAEWVLAYMLFTKFFYLLGLSAIMQVFFGYFASHFISNSWLMWFIISI
       ::.: ::: .::.::: :::.:::.:::.:::.::::.::.::: ::::::::::.::..
NSP3   SSFKWDLTAFGLVAEWFLAYILFTRFFYVLGLAAIMQLFFSYFAVHFISNSWLMWLIINL
          1500      1510      1520      1530      1540      1550

             1540      1550      1560      1570      1580      1590
NSP3   VQMAPVSAMVRMYIFFASFYYIWKSYVHIMDGCTSSTCMMCYKRNRATRVECTTIVNGMK
       :::::.:::::::::::::::.::::::..:::.::::::::::::::::::::::::..
NSP3   VQMAPISAMVRMYIFFASFYYVWKSYVHVVDGCNSSTCMMCYKRNRATRVECTTIVNGVR
          1560      1570      1580      1590      1600      1610

             1600      1610      1620      1630      1640      1650
NSP3   RSFYVYANGGRGFCKTHNWNCLNCDTFCTGSTFISDEVARDLSLQFKRPINPTDQSSYIV
       ::::::::::.:::: :::::.::::::.:::::::::::::::::::::::::::::::
NSP3   RSFYVYANGGKGFCKLHNWNCVNCDTFCAGSTFISDEVARDLSLQFKRPINPTDQSSYIV
          1620      1630      1640      1650      1660      1670

             1660      1670      1680      1690      1700      1710
NSP3   DSVAVKNGALHLYFDKAGQKTYERHPLSHFVNLDNLRANNTKGSLPINVIVFDGKSKCDE
       :::.::::..::::::::::::::: ::::::::::::::::::::::::::::::::.:
NSP3   DSVTVKNGSIHLYFDKAGQKTYERHSLSHFVNLDNLRANNTKGSLPINVIVFDGKSKCEE
          1680      1690      1700      1710      1720      1730

             1720      1730      1740      1750      1760      1770
NSP3   SASKSASVYYSQLMCQPILLLDQALVSDVGDSTEVSVKMFDAYVDTFSATFSVPMEKLKA
       :..:::::::::::::::::::::::::::::.::.::::::::.:::.::.:::::::.
NSP3   SSAKSASVYYSQLMCQPILLLDQALVSDVGDSAEVAVKMFDAYVNTFSSTFNVPMEKLKT
          1740      1750      1760      1770      1780      1790

             1780      1790      1800      1810      1820      1830
NSP3   LVATAHSELAKGVALDGVLSTFVSAARQGVVDTDVDTKDVIECLKLSHHSDLEVTGDSCN
       :::::..::::.:.::.:::::.:::::: ::.::.::::.:::::::.::.::::::::
NSP3   LVATAEAELAKNVSLDNVLSTFISAARQGFVDSDVETKDVVECLKLSHQSDIEVTGDSCN
          1800      1810      1820      1830      1840      1850

             1840      1850      1860      1870      1880      1890
NSP3   NFMLTYNKVENMTPRDLGACIDCNARHINAQVAKSHNVSLIWNVKDYMSLSEQLRKQIRS
       :.:::::::::::::::::::::.:::::::::::::..:::::::.:::::::::::::
NSP3   NYMLTYNKVENMTPRDLGACIDCSARHINAQVAKSHNIALIWNVKDFMSLSEQLRKQIRS
          1860      1870      1880      1890      1900      1910

             1900      1910      1920
NSP3   AAKKNNIPFRLTCATTRQVVNVITTKISLKGG
       ::::::.::.::::::::::::.::::.::::
NSP3   AAKKNNLPFKLTCATTRQVVNVVTTKIALKGG
          1920      1930      1940

>--
```

```
 Waterman-Eggert score: 90;  28.2 bits; E(1) <  0.012
22.2% identity (45.8% similar) in 216 aa overlap (866-1071:852-1062)

         870       880       890       900       910       920
NSP3   AANFCALILAYSNKTVGELGDVRETMTHLLQHANLESAKRVLNVVCKHCGQKTTTLTGVE
       : : : :  :  .    ::     ..     .:    :     ..  .:.. .  :  :.
NSP3   ADNNCYLATALLTLQQIELKFNPPALQDAYYRARAGEAANFCALILAYCNKTVGELGDVR
             860       870       880       890       900       910

          930        940        950       960       970        980
NSP3   AVM-YM-GTLSYDNLKTGVSIPC-VCGRDATQYLVQQESSFVMMSAPPAEYKLQ-QGTFL
        .: :.    . :. :  ... : .::.. :  :   :. . : .    ..:   :
NSP3   ETMSYLFQHANLDSCKRVLNVVCKTCGQQQTT-LKGVEAVMYMGTLSYEQFKKGVQIPCT
             920       930       940        950       960       970

             990        1000      1010      1020      1030
NSP3   CANEYTGNY--QCGHYTHITAKETLYRIDGAHLTKMSEYKGPVTDVFYKETSYTTTIKPV
       :... :     : . .. ..:  . :..  . .:  ::: :      ::. .   :.
NSP3   CGKQATKYLVQQESPFVMMSAPPAQYELKHGTFTCASEYTGNYQCGHYKHITSKETL---
              980       990      1000      1010      1020

    1040      1050          1060      1070
NSP3   SYKLDGVTYTEIE----PKLDGYYKKDNAYYTEQPI
        : .::.  :.      :  : .:...   : .:.
NSP3   -YCIDGALLTKSSEYKGPITDVFYKENSYTTTIKPV
       1030      1040      1050      1060

>--
 Waterman-Eggert score: 88;  27.7 bits; E(1) <  0.017
23.2% identity (53.0% similar) in 181 aa overlap (870-1039:926-1094)

     870       880       890       900         910       920
NSP3   CALILAYSNKTVGELGDVRETMTHLLQHANL--ESAKRVLNVVCKHCGQKTTT-LTGVEA
       :  .:    :: :.   . . .  ..  ..:  :. :. ... :  ::...:  :.  :.
NSP3   CKRVLNVVCKTCGQQQTTLKGVEAVMYMGTLSYEQFKKGVQIPCT-CGKQATKYLVQQES
         930       940       950       960       970        980

          930       940       950        960       970       980
NSP3   --VMYMGTLSYDNLKTGVSIPCVCGRDATQYLVQ-QESSFVMMSAPPAEYKLQQGTFLCA
         ::. .  .  .:: : .. :     :..:  . : . .  ...  . : .. . .  .
NSP3   PFVMMSAPPAQYELKHG-TFTC-----ASEYTGNYQCGHYKHITSKETLYCIDGALLTKS
          990      1000            1010      1020      1030

                990      1000      1010      1020      1030
NSP3   NEYTGN-----YQCGHYTHITAKETLYRIDGAHLTKMSEYKGPVTDVFYKETSYTTTIKP
       .:: :      :. . ::  : : . :..::.  :...    :  : .::. .   : .:
NSP3   SEYKGPITDVFYKENSYT-TTIKPVTYKLDGVVCTEID----PKLDNYYKKDNSYFTEQP
     1040      1050       1060      1070          1080      1090


NSP3   V
       .
NSP3   I
```

```
1922 residues in 1 query   sequences
1945 residues in 1 library sequences
 Scomplib [36.3.5e Nov, 2012(preload8)]
 start: Fri May  8 23:53:10 2020 done: Fri May  8 23:53:11 2020
 Total Scan time:  0.400 Total Display time:  0.170

Function used was LALIGN [36.3.5e Nov, 2012(preload8)]
```

lalign output for NSP4 SARS COV vs. NSP4 SARS COV2

[EMBnet-Server] Date: Fri May 8 23:55:53 2020

```
# bin/lalign36 -E 10.0 -f -12 -g -2 5147.1.seq 5147.2.seq -J -K 3
LALIGN finds non-overlapping local alignments
 version 36.3.5e Nov, 2012(preload8)
Please cite:
 X. Huang and W. Miller (1991) Adv. Appl. Math. 12:373-381

Query: 5147.1.seq
  1>>>NSP4 SARS CoV 500 bp - 500 aa
Library: 5147.2.seq
      500 residues in     1 sequences

Statistics: (shuffled [500]) MLE statistics: Lambda= 0.1594;  K=0.01801
 statistics sampled from 1 (1) to 500 sequences
Threshold: E() < 10 score: 38
Algorithm: Smith-Waterman (SSE2, Michael Farrar 2006) (7.2 Nov 2010)
Parameters: BL50 matrix (15:-5), open/ext: -12/-2
 Scan time:  0.050

>>NSP4 SARS CoV-2 500 bp                                  (500 aa)
 Waterman-Eggert score: 2853;  661.7 bits; E(1) <  1.6e-194
80.0% identity (95.0% similar) in 500 aa overlap (1-500:1-500)

               10        20        30        40        50        60
NSP4   KIVSTCFKLMLKATLLCVLAALVCYIVMPVHTLSIHDGYTNEIIGYKAIQDGVTRDIIST
       :::.. .: ..:.::. ...: . :.. :::..: :  ...::::::::. :::::: ::
NSP4   KIVNNWLKQLIKVTLVFLFVAAIFYLITPVHVMSKHTDFSSEIIGYKAIDGGVTRDIAST
               10        20        30        40        50        60

               70        80        90       100       110       120
NSP4   DDCFANKHAGFDAWFSQRGGSYKNDKSCPVVAAIITREIGFIVPGLPGTVLRAINGDFLH
       : ::::::: ::.::::::::: :::.::..::.:::.::.:::::::.::. ::::::
NSP4   DTCFANKHADFDTWFSQRGGSYTNDKACPLIAAVITREVGFVVPGLPGTILRTTNGDFLH
               70        80        90       100       110       120

              130       140       150       160       170       180
NSP4   FLPRVFSAVGNICYTPSKLIEYSDFATSACVLAAECTIFKDAMGKPVPYCYDTNLLEGSI
       ::::::::::::::::::::::.::::::::::::::::::: :::::::::::.::::.
NSP4   FLPRVFSAVGNICYTPSKLIEYTDFATSACVLAAECTIFKDASGKPVPYCYDTNVLEGSV
              130       140       150       160       170       180

              190       200       210       220       230       240
NSP4   SYSELRPDTRYVLMDGSIIQFPNTYLEGSVRVVTTFDAEYCRHGTCERSEVGICLSTSGR
       .:  :::::::::::::::::::::::::::::::::.::::::::::::.:.:.:::::
NSP4   AYESLRPDTRYVLMDGSIIQFPNTYLEGSVRVVTTFDSEYCRHGTCERSEAGVCVSTSGR
              190       200       210       220       230       240

              250       260       270       280       290       300
NSP4   WVLNNEHYRALSGVFCGVDAMNLIANIFTPLVQPVGALDVSASVVAGGIIAILVTCAAYY
       :::::..::.: ::::::::.::..:.::::.::.::::.:::.:::::.::.::: :::
NSP4   WVLNNDYYRSLPGVFCGVDAVNLLTNMFTPLIQPIGALDISASIVAGGIVAIVVTCLAYY
```

```
              250       260       270       280       290       300

              310       320       330       340       350       360
NSP4   FMKFRRVFGEYNHVVAANALLFLMSFTILCLVPAYSFLPGVYSVFYLYLTFYFTNDVSFL
       ::.:::.::::.:::: :.::::::::.:::.:.::::::::::.:::::::.:::::::
NSP4   FMRFRRAFGEYSHVVAFNTLLFLMSFTVLCLTPVYSFLPGVYSVIYLYLTFYLTNDVSFL
              310       320       330       340       350       360

              370       380       390       400       410       420
NSP4   AHLQWFAMFSPIVPFWITAIYVFCISLKHCHWFFNNYLRKRVMFNGVTFSTFEEAALCTF
       ::.::..::.:.::::::  :..::: :: .:::.:::..::.::::.::::::::::::
NSP4   AHIQWMVMFTPLVPFWITIAYIICISTKHFYWFFSNYLKRRVVFNGVSFSTFEEAALCTF
              370       380       390       400       410       420

              430       440       450       460       470       480
NSP4   LLNKEMYLKLRSETLLPLTQYNRYLALYNKYKYFSGALDTTSYREAACCHLAKALNDFSN
       ::::::::::::..:::::::::::::::::::::::.::::::::::::::::::::::
NSP4   LLNKEMYLKLRSDVLLPLTQYNRYLALYNKYKYFSGAMDTTSYREAACCHLAKALNDFSN
              430       440       450       460       470       480

              490       500
NSP4   SGADVLYQPPQTSITSAVLQ
       ::.:::::::::::::::::
NSP4   SGSDVLYQPPQTSITSAVLQ
              490       500

>--
 Waterman-Eggert score: 48;  16.8 bits; E(1) <  0.88
35.0% identity (70.0% similar) in 20 aa overlap (113-132:242-261)

            120       130
NSP4   AINGDFLHFLPRVFSAVGNI
       ..:.:. . :: :: .:  .
NSP4   VLNNDYYRSLPGVFCGVDAV
             250       260

>--
 Waterman-Eggert score: 43;  15.7 bits; E(1) <  0.99
20.0% identity (66.7% similar) in 30 aa overlap (440-469:389-418)

     440       450       460
NSP4   QYNRYLALYNKYKYFSGALDTTSYREAACC
       ..  ... : : .   .... ....::: :
NSP4   HFYWFFSNYLKRRVVFNGVSFSTFEEAALC
      390       400       410



500 residues in 1 query   sequences
500 residues in 1 library sequences
 Scomplib [36.3.5e Nov, 2012(preload8)]
 start: Fri May  8 23:55:54 2020 done: Fri May  8 23:55:54 2020
 Total Scan time:  0.050 Total Display time:  0.030

Function used was LALIGN [36.3.5e Nov, 2012(preload8)]
```

lalign output for NSP5 SARS COV vs. NSP5 SARS COV2

[EMBnet-Server] Date: Fri May 8 23:57:29 2020

```
# bin/lalign36 -E 10.0 -f -12 -g -2 5109.1.seq 5109.2.seq -J -K 3
LALIGN finds non-overlapping local alignments
 version 36.3.5e Nov, 2012(preload8)
Please cite:
 X. Huang and W. Miller (1991) Adv. Appl. Math. 12:373-381

Query: 5109.1.seq
  1>>>NSP5 SARS CoV 306 bp - 306 aa
Library: 5109.2.seq
      306 residues in     1 sequences

Statistics: (shuffled [500]) MLE statistics: Lambda= 0.1754;  K= 0.027
 statistics sampled from 1 (1) to 500 sequences
Threshold: E() < 10 score: 32
Algorithm: Smith-Waterman (SSE2, Michael Farrar 2006) (7.2 Nov 2010)
Parameters: BL50 matrix (15:-5), open/ext: -12/-2
 Scan time:  0.020

>>NSP5 SARS CoV-2 306 bp                                  (306 aa)
 Waterman-Eggert score: 2043;  522.2 bits; E(1) <  6e-153
96.1% identity (99.7% similar) in 306 aa overlap (1-306:1-306)

               10        20        30        40        50        60
NSP5   SGFRKMAFPSGKVEGCMVQVTCGTTTLNGLWLDDTVYCPRHVICTAEDMLNPNYEDLLIR
       ::::::::::::::::::::::::::::::::::.::::::::::.::::::::::::::
NSP5   SGFRKMAFPSGKVEGCMVQVTCGTTTLNGLWLDDVVYCPRHVICTSEDMLNPNYEDLLIR
               10        20        30        40        50        60

               70        80        90       100       110       120
NSP5   KSNHSFLVQAGNVQLRVIGHSMQNCLLRLKVDTSNPKTPKYKFVRIQPGQTFSVLACYNG
       ::::.::::::::::::::::::::.:.:::::.::::::::::::::::::::::::::
NSP5   KSNHNFLVQAGNVQLRVIGHSMQNCVLKLKVDTANPKTPKYKFVRIQPGQTFSVLACYNG
               70        80        90       100       110       120

              130       140       150       160       170       180
NSP5   SPSGVYQCAMRPNHTIKGSFLNGSCGSVGFNIDYDCVSFCYMHHMELPTGVHAGTDLEGK
       ::::::::::::: ::::::::::::::::::::::::::::::::::::::::::::::.
NSP5   SPSGVYQCAMRPNFTIKGSFLNGSCGSVGFNIDYDCVSFCYMHHMELPTGVHAGTDLEGN
              130       140       150       160       170       180

              190       200       210       220       230       240
NSP5   FYGPFVDRQTAQAAGTDTTITLNVLAWLYAAVINGDRWFLNRFTTTLNDFNLVAMKYNYE
       :::::::::::::::::::::.::::::::::::::::::::::::::::::::::::::
NSP5   FYGPFVDRQTAQAAGTDTTITVNVLAWLYAAVINGDRWFLNRFTTTLNDFNLVAMKYNYE
              190       200       210       220       230       240

              250       260       270       280       290       300
NSP5   PLTQDHVDILGPLSAQTGIAVLDMCAALKELLQNGMNGRTILGSTILEDEFTPFDVVRQC
       :::::::::::::::::::::::::.::::::::::::::::::..::::::::::::::
NSP5   PLTQDHVDILGPLSAQTGIAVLDMCASLKELLQNGMNGRTILGSALLEDEFTPFDVVRQC
```

```
             250       260       270       280       290       300


NSP5   SGVTFQ
       ::::::
NSP5   SGVTFQ


>--
 Waterman-Eggert score: 61;  20.6 bits; E(1) <  0.055
28.6% identity (61.9% similar) in 42 aa overlap (123-163:1-41)

           130       140       150        160
NSP5   SGVYQCAMRPNHTIKGSFLNGSCGSVGFN-IDYDCVSFCYMH
       ::  . :. :.  ..: ... .::.. .: .  : : .:  :
NSP5   SGFRKMAF-PSGKVEGCMVQVTCGTTTLNGLWLDDVVYCPRH
               10        20        30        40

>--
 Waterman-Eggert score: 54;  18.9 bits; E(1) <  0.18
26.2% identity (61.9% similar) in 42 aa overlap (1-41:123-163)

               10        20        30        40
NSP5   SGFRKMAF-PSGKVEGCMVQVTCGTTTLNGLWLDDTVYCPRH
       ::  . :. :.  ..: ... .::.. .: .  : . .:  :
NSP5   SGVYQCAMRPNFTIKGSFLNGSCGSVGFN-IDYDCVSFCYMH
           130       140       150        160



306 residues in 1 query   sequences
306 residues in 1 library sequences
 Scomplib [36.3.5e Nov, 2012(preload8)]
 start: Fri May  8 23:57:30 2020 done: Fri May  8 23:57:30 2020
 Total Scan time:  0.020 Total Display time:  0.010

Function used was LALIGN [36.3.5e Nov, 2012(preload8)]
```

lalign output for NSP6 SARS COV vs. NSP6 SARS COV2

[EMBnet-Server] Date: Sat May 9 0:01:32 2020

```
# bin/lalign36 -E 10.0 -f -12 -g -2 6675.1.seq 6675.2.seq -J -K 3
LALIGN finds non-overlapping local alignments
 version 36.3.5e Nov, 2012(preload8)
Please cite:
 X. Huang and W. Miller (1991) Adv. Appl. Math. 12:373-381

Query: 6675.1.seq
  1>>>NSP6 SARS CoV 290 bp - 290 aa
Library: 6675.2.seq
      290 residues in     1 sequences

Statistics: (shuffled [500]) MLE statistics: Lambda= 0.1386;  K=0.0262
 statistics sampled from 1 (1) to 500 sequences
Threshold: E() < 10 score: 39
Algorithm: Smith-Waterman (SSE2, Michael Farrar 2006) (7.2 Nov 2010)
Parameters: BL50 matrix (15:-5), open/ext: -12/-2
 Scan time:  0.020

>>NSP6 SARS CoV-2 290 bp                                  (290 aa)
 Waterman-Eggert score: 1750;  355.2 bits; E(1) <  1e-102
88.2% identity (98.3% similar) in 287 aa overlap (4-290:4-290)

               10        20        30        40        50        60
NSP6   KKIVKGTHHWMLLTFLTSLLILVQSTQWSLFFFVYENAFLPFTLGIMAIAACAMLLVKHK
       :. .::::::.:::.:::::.::::::::::::.::::::::..::.:..: ::..::::
NSP6   KRTIKGTHHWLLLTILTSLLVLVQSTQWSLFFFLYENAFLPFAMGIIAMSAFAMMFVKHK
               10        20        30        40        50        60

               70        80        90       100       110       120
NSP6   HAFLCLFLLPSLATVAYFNMVYMPASWVMRIMTWLELADTSLSGYRLKDCVMYASALVLL
       :::::::::::::::::::::::::::::::::::...:::::::..:::::::::.:::
NSP6   HAFLCLFLLPSLATVAYFNMVYMPASWVMRIMTWLDMVDTSLSGFKLKDCVMYASAVVLL
               70        80        90       100       110       120

              130       140       150       160       170       180
NSP6   ILMTARTVYDDAARRVWTLMNVITLVYKVYYGNALDQAISMWALVISVTSNYSGVVTTIM
       :::::::::::.::::::::::.:::::::::::::::::::::.:::::::::::::.:
NSP6   ILMTARTVYDDGARRVWTLMNVLTLVYKVYYGNALDQAISMWALIISVTSNYSGVVTTVM
              130       140       150       160       170       180

              190       200       210       220       230       240
NSP6   FLARAIVFVCVEYYPLLFITGNTLQCIMLVYCFLGYCCCCYFGLFCLLNRYFRLTLGVYD
       ::::.:::.:::: :..::::::::::::::::::: : :::::::::::::::::::::
NSP6   FLARGIVFMCVEYCPIFFITGNTLQCIMLVYCFLGYFCTCYFGLFCLLNRYFRLTLGVYD
              190       200       210       220       230       240

              250       260       270       280       290
NSP6   YLVSTQEFRYMNSQGLLPPKSSIDAFKLNIKLLGIGGKPCIKVATVQ
       ::::::::::::::::::::.:::::::::::::.::::::::::::
NSP6   YLVSTQEFRYMNSQGLLPPKNSIDAFKLNIKLLGVGGKPCIKVATVQ
```

```
           250       260       270       280       290

>--
 Waterman-Eggert score: 54;  16.1 bits; E(1) <  0.71
15.2% identity (56.5% similar) in 46 aa overlap (199-242:183-228)

      200       210         220       230       240
NSP6   LLFITGNTLQCIML--VYCFLGYCCCCYFGLFCLLNRYFRLTLGVY
       ...  : ...:.    .. . :    : . ..:.:. .    .:..
NSP6   MFLARGIVFMCVEYCPIFFITGNTLQCIMLVYCFLGYFCTCYFGLF
            190       200       210       220

>--
 Waterman-Eggert score: 49;  15.1 bits; E(1) <  0.92
17.5% identity (50.2% similar) in 211 aa overlap (17-217:41-245)

         20        30        40          50        60        70
NSP6   TFLTSLLILVQSTQWSLFFFVYENAFLPFTL--GIMAIAACAMLLVKHKHAFLCLFLLPS
       .::   . ..  . ....:  ...::: . :  .. ..:   :. .  . ..  .  :
NSP6   AFLPFAMGIIAMSAFAMMFVKHKHAFLCLFLLPSLATVAYFNMVYMPASWVMRIMTWLDM
               50        60        70        80        90       100

            80            90       100       110       120
NSP6   LAT-VAYFNM----VYMPASWVMRIMTWLELADTSLSGYRLKDCVMYASALVLLILMTAR
       . : .. :..    .:  :  .. .::   . :   .: :    .: . .::  . .
NSP6   VDTSLSGFKLKDCVMYASAVVLLILMTARTVYD---DGARRVWTLMNVLTLVYKVYYGNA
              110       120       130          140       150

     130       140       150       160       170          180
NSP6   TVYDDAARRVWTLMNVITLVYKVYYGNALDQAISMWALVISVTSNY---SGVVTTIMFLA
           : :  .:.:.  .:  :.    ...  : ..  . .     .   ....  ::..
NSP6   L---DQAISMWALIISVTSNYSGVVTTVMFLARGIVFMCVEYCPIFFITGNTLQCIMLVY
          160       170       180       190       200       210

        190       200       210
NSP6   RAIVFVCVEYYPLLFITGNTLQCIMLVYCFL
         . . :. :. :. . .  ..  . :: .:
NSP6   CFLGYFCTCYFGLFCLLNRYFRLTLGVYDYL
          220       230       240



290 residues in 1 query   sequences
290 residues in 1 library sequences
 Scomplib [36.3.5e Nov, 2012(preload8)]
 start: Sat May  9 00:01:33 2020 done: Sat May  9 00:01:33 2020
 Total Scan time:  0.020 Total Display time:  0.020

Function used was LALIGN [36.3.5e Nov, 2012(preload8)]
```

lalign output for NSP7 SARS COV vs. NSP7 SARS COV2

[EMBnet-Server] Date: Sat May 9 0:03:08 2020

```
# bin/lalign36 -E 10.0 -f -12 -g -2 5099.1.seq 5099.2.seq -J -K 3
LALIGN finds non-overlapping local alignments
 version 36.3.5e Nov, 2012(preload8)
Please cite:
 X. Huang and W. Miller (1991) Adv. Appl. Math. 12:373-381

Query: 5099.1.seq
  1>>>NSP7 SARS CoV 83 bp - 83 aa
Library: 5099.2.seq
       83 residues in     1 sequences

Statistics: (shuffled [500]) MLE statistics: Lambda= 0.1692;  K=0.0695
 statistics sampled from 1 (1) to 500 sequences
Threshold: E() < 10 score: 23
Algorithm: Smith-Waterman (SSE2, Michael Farrar 2006) (7.2 Nov 2010)
Parameters: BL50 matrix (15:-5), open/ext: -12/-2
 Scan time:  0.000

>>NSP7 SARS CoV-2 83 bp                               (83 aa)
 Waterman-Eggert score: 513;  129.1 bits; E(1) <  9.6e-36
98.8% identity (100.0% similar) in 83 aa overlap (1-83:1-83)

               10        20        30        40        50        60
NSP7   SKMSDVKCTSVVLLSVLQQLRVESSSKLWAQCVQLHNDILLAKDTTEAFEKMVSLLSVLL
       ::::::::::::::::::::::::::::::::::::::::::::::::::::::::::::
NSP7   SKMSDVKCTSVVLLSVLQQLRVESSSKLWAQCVQLHNDILLAKDTTEAFEKMVSLLSVLL
               10        20        30        40        50        60

               70        80
NSP7   SMQGAVDINRLCEEMLDNRATLQ
       :::::::::.:::::::::::::
NSP7   SMQGAVDINKLCEEMLDNRATLQ
               70        80

>--
 Waterman-Eggert score: 30;  11.2 bits; E(1) <  0.95
54.5% identity (81.8% similar) in 11 aa overlap (53-63:11-21)

             60
NSP7   VSLLSVLLSMQ
       : ::::: ...
NSP7   VVLLSVLQQLR
               20

>--
 Waterman-Eggert score: 30;  11.2 bits; E(1) <  0.95
54.5% identity (81.8% similar) in 11 aa overlap (11-21:53-63)

               20
NSP7   VVLLSVLQQLR
```

```
       : ::::: ...
NSP7   VSLLSVLLSMQ
             60



83 residues in 1 query   sequences
83 residues in 1 library sequences
 Scomplib [36.3.5e Nov, 2012(preload8)]
 start: Sat May  9 00:03:08 2020 done: Sat May  9 00:03:08 2020
 Total Scan time:  0.000 Total Display time:  0.000

Function used was LALIGN [36.3.5e Nov, 2012(preload8)]
```

lalign output for NSP8 SARS COV vs. NSP8 SARS COV2

[EMBnet-Server] Date: Sat May 9 0:04:50 2020

```
# bin/lalign36 -E 10.0 -f -12 -g -2 5098.1.seq 5098.2.seq -J -K 3
LALIGN finds non-overlapping local alignments
 version 36.3.5e Nov, 2012(preload8)
Please cite:
 X. Huang and W. Miller (1991) Adv. Appl. Math. 12:373-381

Query: 5098.1.seq
  1>>>NSP8 SARS CoV 198 bp - 198 aa
Library: 5098.2.seq
      198 residues in     1 sequences

Statistics: (shuffled [500]) MLE statistics: Lambda= 0.1533;  K=0.02416
 statistics sampled from 1 (1) to 500 sequences
Threshold: E() < 10 score: 30
Algorithm: Smith-Waterman (SSE2, Michael Farrar 2006) (7.2 Nov 2010)
Parameters: BL50 matrix (15:-5), open/ext: -12/-2
 Scan time:  0.010

>>NSP8 SARS CoV-2 198 bp                                    (198 aa)
 Waterman-Eggert score: 1220;  275.1 bits; E(1) <  6e-79
97.5% identity (100.0% similar) in 198 aa overlap (1-198:1-198)

               10        20        30        40        50        60
NSP8   AIASEFSSLPSYAAYATAQEAYEQAVANGDSEVVLKKLKKSLNVAKSEFDRDAAMQRKLE
       :::::::::::::.::::::::::::::::::::::::::::::::::::::::::::::
NSP8   AIASEFSSLPSYAAFATAQEAYEQAVANGDSEVVLKKLKKSLNVAKSEFDRDAAMQRKLE
               10        20        30        40        50        60

               70        80        90       100       110       120
NSP8   KMADQAMTQMYKQARSEDKRAKVTSAMQTMLFTMLRKLDNDALNNIINNARDGCVPLNII
       ::::::::::::::::::::::::::::::::::::::::::::::::::::::::::::
NSP8   KMADQAMTQMYKQARSEDKRAKVTSAMQTMLFTMLRKLDNDALNNIINNARDGCVPLNII
               70        80        90       100       110       120

              130       140       150       160       170       180
NSP8   PLTTAAKLMVVVPDYGTYKNTCDGNTFTYASALWEIQQVVDADSKIVQLSEINMDNSPNL
       :::::::::::.:::.::::::::.:::::::::::::::::::::::::::::.:::::::
NSP8   PLTTAAKLMVVIPDYNTYKNTCDGTTFTYASALWEIQQVVDADSKIVQLSEISMDNSPNL
              130       140       150       160       170       180

              190
NSP8   AWPLIVTALRANSAVKLQ
       ::::::::::::::::::
NSP8   AWPLIVTALRANSAVKLQ
              190

>--
 Waterman-Eggert score: 55;  17.5 bits; E(1) <  0.19
23.9% identity (65.2% similar) in 46 aa overlap (57-102:36-81)
```

```
          60        70        80        90       100
NSP8   RKLEKMADQAMTQMYKQARSEDKRAKVTSAMQTMLFTMLRKLDNDA
       .::.:  . : ... ..:  . :  :...  .:... . :. :. :
NSP8   KKLKKSLNVAKSEFDRDAAMQRKLEKMADQAMTQMYKQARSEDKRA
           40        50        60        70        80

>--
 Waterman-Eggert score: 55;  17.5 bits; E(1) <  0.19
23.9% identity (65.2% similar) in 46 aa overlap (36-81:57-102)

           40        50        60        70        80
NSP8   KKLKKSLNVAKSEFDRDAAMQRKLEKMADQAMTQMYKQARSEDKRA
       .::.:  . : ... ..:  . :  :...  .:... . :. :. :
NSP8   RKLEKMADQAMTQMYKQARSEDKRAKVTSAMQTMLFTMLRKLDNDA
          60        70        80        90       100



198 residues in 1 query   sequences
198 residues in 1 library sequences
 Scomplib [36.3.5e Nov, 2012(preload8)]
 start: Sat May  9 00:04:50 2020 done: Sat May  9 00:04:50 2020
 Total Scan time:  0.010 Total Display time:  0.010

Function used was LALIGN [36.3.5e Nov, 2012(preload8)]
```

lalign output for NSP9 SARS COV vs. NSP9 SARS COV2

[EMBnet-Server] Date: Sat May 9 0:07:40 2020

```
# bin/lalign36 -E 10.0 -f -12 -g -2 6675.1.seq 6675.2.seq -J -K 3
LALIGN finds non-overlapping local alignments
 version 36.3.5e Nov, 2012(preload8)
Please cite:
 X. Huang and W. Miller (1991) Adv. Appl. Math. 12:373-381

Query: 6675.1.seq
  1>>>NSP9 SARS CoV 113 bp - 113 aa
Library: 6675.2.seq
      113 residues in     1 sequences

Statistics: (shuffled [500]) MLE statistics: Lambda= 0.2089;  K=0.06941
 statistics sampled from 1 (1) to 500 sequences
Threshold: E() < 10 score: 21
Algorithm: Smith-Waterman (SSE2, Michael Farrar 2006) (7.2 Nov 2010)
Parameters: BL50 matrix (15:-5), open/ext: -12/-2
 Scan time:  0.000

>>NSP9 SARS CoV-2 113 bp                                  (113 aa)
 Waterman-Eggert score: 733;  224.8 bits; E(1) <  2.8e-64
97.3% identity (99.1% similar) in 113 aa overlap (1-113:1-113)

               10        20        30        40        50        60
NSP9   NNELSPVALRQMSCAAGTTQTACTDDNALAYYNNSKGGRFVLALLSDHQDLKWARFPKSD
       :::::::::::::::::::::::::::::::::..::::::::::::: :::::::::::
NSP9   NNELSPVALRQMSCAAGTTQTACTDDNALAYYNTTKGGRFVLALLSDLQDLKWARFPKSD
               10        20        30        40        50        60

               70        80        90       100       110
NSP9   GTGTIYTELEPPCRFVTDTPKGPKVKYLYFIKGLNNLNRGMVLGSLAATVRLQ
       :::::::::::::::::::::::::::::::::::::::::::::::::::::
NSP9   GTGTIYTELEPPCRFVTDTPKGPKVKYLYFIKGLNNLNRGMVLGSLAATVRLQ
               70        80        90       100       110

>--
 Waterman-Eggert score: 26;  11.7 bits; E(1) <  0.98
26.1% identity (43.5% similar) in 23 aa overlap (16-38:15-37)

          20        30
NSP9   AGTTQTACTDDNALAYYNNSKGG
       :. :  .   :.    : :.  :
NSP9   AAGTTQTACTDDNALAYYNTTKG
           20        30

>--
 Waterman-Eggert score: 25;  11.4 bits; E(1) <  0.99
27.8% identity (50.0% similar) in 18 aa overlap (59-76:24-41)

       60        70
NSP9   SDGTGTIYTELEPPCRFV
```

```
       .: ..  : .     :::
NSP9   TDDNALAYYNTTKGGRFV
            30        40



113 residues in 1 query   sequences
113 residues in 1 library sequences
 Scomplib [36.3.5e Nov, 2012(preload8)]
 start: Sat May  9 00:07:40 2020 done: Sat May  9 00:07:40 2020
 Total Scan time:  0.000 Total Display time:  0.010

Function used was LALIGN [36.3.5e Nov, 2012(preload8)]
```

lalign output for NSP10 SARS COV vs. NSP10 SARS COV2

[EMBnet-Server] Date: Sat May 9 0:09:58 2020

```
# bin/lalign36 -E 10.0 -f -12 -g -2 5099.1.seq 5099.2.seq -J -K 3
LALIGN finds non-overlapping local alignments
 version 36.3.5e Nov, 2012(preload8)
Please cite:
 X. Huang and W. Miller (1991) Adv. Appl. Math. 12:373-381

Query: 5099.1.seq
  1>>>NSP10 SARS CoV 139 bp - 139 aa
Library: 5099.2.seq
      139 residues in     1 sequences

Statistics: (shuffled [500]) MLE statistics: Lambda= 0.1369;  K=0.02402
 statistics sampled from 1 (1) to 500 sequences
Threshold: E() < 10 score: 28
Algorithm: Smith-Waterman (SSE2, Michael Farrar 2006) (7.2 Nov 2010)
Parameters: BL50 matrix (15:-5), open/ext: -12/-2
 Scan time:  0.010

>>NSP10 SARS CoV-2 139 bp                                 (139 aa)
 Waterman-Eggert score: 989;  200.8 bits; E(1) <  7.1e-57
97.1% identity (99.3% similar) in 139 aa overlap (1-139:1-139)

               10        20        30        40        50        60
NSP10  AGNATEVPANSTVLSFCAFAVDPAKAYKDYLASGGQPITNCVKMLCTHTGTGQAITVTPE
       :::::::::::::::::::::: :::::::::::::::::::::::::::::::::::::
NSP10  AGNATEVPANSTVLSFCAFAVDAAKAYKDYLASGGQPITNCVKMLCTHTGTGQAITVTPE
               10        20        30        40        50        60

               70        80        90       100       110       120
NSP10  ANMDQESFGGASCCLYCRCHIDHPNPKGFCDLKGKYVQIPTTCANDPVGFTLRNTVCTVC
       ::::::::::::::::::::::::::::::::::::::::::::::::::::.:::::::
NSP10  ANMDQESFGGASCCLYCRCHIDHPNPKGFCDLKGKYVQIPTTCANDPVGFTLKNTVCTVC
               70        80        90       100       110       120

              130
NSP10  GMWKGYGCSCDQLREPLMQ
       :::::::::::::::::..:
NSP10  GMWKGYGCSCDQLREPMLQ
              130



139 residues in 1 query   sequences
139 residues in 1 library sequences
 Scomplib [36.3.5e Nov, 2012(preload8)]
 start: Sat May  9 00:09:58 2020 done: Sat May  9 00:09:58 2020
 Total Scan time:  0.010 Total Display time:  0.000

Function used was LALIGN [36.3.5e Nov, 2012(preload8)]
```

lalign output for NSP11 SARS COV vs. NSP11 SARS COV2

[EMBnet-Server] Date: Sat May 9 0:11:21 2020

```
# bin/lalign36 -E 10.0 -f -12 -g -2 5148.1.seq 5148.2.seq -J -K 3
LALIGN finds non-overlapping local alignments
 version 36.3.5e Nov, 2012(preload8)
Please cite:
 X. Huang and W. Miller (1991) Adv. Appl. Math. 12:373-381

Query: 5148.1.seq
  1>>>NSP11 SARS CoV 13 bp - 13 aa
Library: 5148.2.seq
       13 residues in     1 sequences

Statistics: (shuffled [500]) MLE statistics: Lambda= 0.2930;  K=0.7519
 statistics sampled from 1 (1) to 500 sequences
Threshold: E() < 10 score: 9
Algorithm: Smith-Waterman (SSE2, Michael Farrar 2006) (7.2 Nov 2010)
Parameters: BL50 matrix (15:-5), open/ext: -12/-2
 Scan time:  0.000

>>NSP11 SARS CoV-2 13 bp                                  (13 aa)
 Waterman-Eggert score: 71;  30.4 bits; E(1) <  1.2e-07
84.6% identity (100.0% similar) in 13 aa overlap (1-13:1-13)

               10
NSP11  SADASTFLNGFAV
       ::::..:::::::
NSP11  SADAQSFLNGFAV
               10



13 residues in 1 query   sequences
13 residues in 1 library sequences
 Scomplib [36.3.5e Nov, 2012(preload8)]
 start: Sat May  9 00:11:21 2020 done: Sat May  9 00:11:21 2020
 Total Scan time:  0.000 Total Display time:  0.000

Function used was LALIGN [36.3.5e Nov, 2012(preload8)]
```

lalign output for NSP12 SARS COV vs. NSP12 SARS COV2

[EMBnet-Server] Date: Sat May 9 0:13:30 2020

```
# bin/lalign36 -E 10.0 -f -12 -g -2 6675.1.seq 6675.2.seq -J -K 3
LALIGN finds non-overlapping local alignments
 version 36.3.5e Nov, 2012(preload8)
Please cite:
 X. Huang and W. Miller (1991) Adv. Appl. Math. 12:373-381

Query: 6675.1.seq
  1>>>NSP12 SARS CoV 932 bp - 932 aa
Library: 6675.2.seq
      932 residues in     1 sequences

Statistics: (shuffled [500]) MLE statistics: Lambda= 0.1584;  K=0.01142
 statistics sampled from 1 (1) to 500 sequences
Threshold: E() < 10 score: 44
Algorithm: Smith-Waterman (SSE2, Michael Farrar 2006) (7.2 Nov 2010)
Parameters: BL50 matrix (15:-5), open/ext: -12/-2
 Scan time:  0.160

>>NSP12 SARS CoV-2 932 bp                                (932 aa)
 Waterman-Eggert score: 6186;  1420.1 bits; E(1) <  0
96.4% identity (99.4% similar) in 932 aa overlap (1-932:1-932)

               10        20        30        40        50        60
NSP12  SADASTFLNRVCGVSAARLTPCGTGTSTDVVYRAFDIYNEKVAGFAKFLKTNCCRFQEKD
       ::::..::::::::::::::::::::::::::::::::::.:::::::::::::::::::
NSP12  SADAQSFLNRVCGVSAARLTPCGTGTSTDVVYRAFDIYNDKVAGFAKFLKTNCCRFQEKD
               10        20        30        40        50        60

               70        80        90       100       110       120
NSP12  EEGNLLDSYFVVKRHTMSNYQHEETIYNLVKDCPAVAVHDFFKFRVDGDMVPHISRQRLT
       :. ::.::::::::::.::::::::::::.::::::: :::::::.::::::::::::::
NSP12  EDDNLIDSYFVVKRHTFSNYQHEETIYNLLKDCPAVAKHDFFKFRIDGDMVPHISRQRLT
               70        80        90       100       110       120

              130       140       150       160       170       180
NSP12  KYTMADLVYALRHFDEGNCDTLKEILVTYNCCDDDYFNKKDWYDFVENPDILRVYANLGE
       ::::::::::::::::::::::::::::::::::::::::::::::::::::::::::::
NSP12  KYTMADLVYALRHFDEGNCDTLKEILVTYNCCDDDYFNKKDWYDFVENPDILRVYANLGE
              130       140       150       160       170       180

              190       200       210       220       230       240
NSP12  RVRQSLLKTVQFCDAMRDAGIVGVLTLDNQDLNGNWYDFGDFVQVAPGCGVPIVDSYYSL
       ::::.::::::::::::.::::::::::::::::::::::::.:..:: :::.:::::::
NSP12  RVRQALLKTVQFCDAMRNAGIVGVLTLDNQDLNGNWYDFGDFIQTTPGSGVPVVDSYYSL
              190       200       210       220       230       240

              250       260       270       280       290       300
NSP12  LMPILTLTRALAAESHMDADLAKPLIKWDLLKYDFTEERLCLFDRYFKYWDQTYHPNCIN
       :::::::::::.::::.:.::.:: ::::::::::::::: :::::::::::::::::.:
NSP12  LMPILTLTRALTAESHVDTDLTKPYIKWDLLKYDFTEERLKLFDRYFKYWDQTYHPNCVN
```

```
              250       260       270       280       290       300

              310       320       330       340       350       360
NSP12  CLDDRCILHCANFNVLFSTVFPPTSFGPLVRKIFVDGVPFVVSTGYHFRELGVVHNQDVN
       ::::::::::::::::::::::::::::::::::::::::::::::::::::::::::::
NSP12  CLDDRCILHCANFNVLFSTVFPPTSFGPLVRKIFVDGVPFVVSTGYHFRELGVVHNQDVN
              310       320       330       340       350       360

              370       380       390       400       410       420
NSP12  LHSSRLSFKELLVYAADPAMHAASGNLLLDKRTTCFSVAALTNNVAFQTVKPGNFNKDFY
       ::::::::::::::::::::::::::::::::::::::::::::::::::::::::::::
NSP12  LHSSRLSFKELLVYAADPAMHAASGNLLLDKRTTCFSVAALTNNVAFQTVKPGNFNKDFY
              370       380       390       400       410       420

              430       440       450       460       470       480
NSP12  DFAVSKGFFKEGSSVELKHFFFAQDGNAAISDYDYYRYNLPTMCDIRQLLFVVEVVDKYF
       ::::::::::::::::::::::::::::::::::::::::::::::::::::::::::::
NSP12  DFAVSKGFFKEGSSVELKHFFFAQDGNAAISDYDYYRYNLPTMCDIRQLLFVVEVVDKYF
              430       440       450       460       470       480

              490       500       510       520       530       540
NSP12  DCYDGGCINANQVIVNNLDKSAGFPFNKWGKARLYYDSMSYEDQDALFAYTKRNVIPTIT
       ::::::::::::::::::::::::::::::::::::::::::::::::::::::::::::
NSP12  DCYDGGCINANQVIVNNLDKSAGFPFNKWGKARLYYDSMSYEDQDALFAYTKRNVIPTIT
              490       500       510       520       530       540

              550       560       570       580       590       600
NSP12  QMNLKYAISAKNRARTVAGVSICSTMTNRQFHQKLLKSIAATRGATVVIGTSKFYGGWHN
       ::::::::::::::::::::::::::::::::::::::::::::::::::::::::::::
NSP12  QMNLKYAISAKNRARTVAGVSICSTMTNRQFHQKLLKSIAATRGATVVIGTSKFYGGWHN
              550       560       570       580       590       600

              610       620       630       640       650       660
NSP12  MLKTVYSDVETPHLMGWDYPKCDRAMPNMLRIMASLVLARKHNTCCNLSHRFYRLANECA
       ::::::::::.:::::::::::::::::::::::::::::::.:::.:::::::::::::
NSP12  MLKTVYSDVENPHLMGWDYPKCDRAMPNMLRIMASLVLARKHTTCCSLSHRFYRLANECA
              610       620       630       640       650       660

              670       680       690       700       710       720
NSP12  QVLSEMVMCGGSLYVKPGGTSSGDATTAYANSVFNICQAVTANVNALLSTDGNKIADKYV
       ::::::::::::::::::::::::::::::::::::::::::::::::::::::::::::
NSP12  QVLSEMVMCGGSLYVKPGGTSSGDATTAYANSVFNICQAVTANVNALLSTDGNKIADKYV
              670       680       690       700       710       720

              730       740       750       760       770       780
NSP12  RNLQHRLYECLYRNRDVDHEFVDEFYAYLRKHFSMMILSDDAVVCYNSNYAAQGLVASIK
       :::::::::::::::::: .::.::::::::::::::::::::::.::.::.::::::::
NSP12  RNLQHRLYECLYRNRDVDTDFVNEFYAYLRKHFSMMILSDDAVVCFNSTYASQGLVASIK
              730       740       750       760       770       780

              790       800       810       820       830       840
NSP12  NFKAVLYYQNNVFMSEAKCWTETDLTKGPHEFCSQHTMLVKQGDDYVYLPYPDPSRILGA
       :::.::::::::::::::::::::::::::::::::::::::::::::::::::::::::
NSP12  NFKSVLYYQNNVFMSEAKCWTETDLTKGPHEFCSQHTMLVKQGDDYVYLPYPDPSRILGA
              790       800       810       820       830       840

              850       860       870       880       890       900
```

```
NSP12  GCFVDDIVKTDGTLMIERFVSLAIDAYPLTKHPNQEYADVFHLYLQYIRKLHDELTGHML
       ::::::::::::::::::::::::::::::::::::::::::::::::::::::::::::
NSP12  GCFVDDIVKTDGTLMIERFVSLAIDAYPLTKHPNQEYADVFHLYLQYIRKLHDELTGHML
              850       860       870       880       890       900

              910       920       930
NSP12  DMYSVMLTNDNTSRYWEPEFYEAMYTPHTVLQ
       ::::::::::::::::::::::::::::::::
NSP12  DMYSVMLTNDNTSRYWEPEFYEAMYTPHTVLQ
              910       920       930

>--
 Waterman-Eggert score: 64;  21.1 bits; E(1) <  0.32
28.0% identity (72.0% similar) in 25 aa overlap (730-754:54-78)

     730       740       750
NSP12  CLYRNRDVDHEFVDEFYAYLRKHFS
       : ....: : ...: ...  :. ::
NSP12  CRFQEKDEDDNLIDSYFVVKRHTFS
            60        70

>--
 Waterman-Eggert score: 54;  18.8 bits; E(1) <  0.85
25.9% identity (59.3% similar) in 27 aa overlap (193-219:139-165)

            200       210
NSP12  CDAMRDAGIVGVLTLDNQDLNGNWYDF
       ::....  ..     :.   . .::::
NSP12  CDTLKEILVTYNCCDDDYFNKKDWYDF
     140       150       160



932 residues in 1 query   sequences
932 residues in 1 library sequences
 Scomplib [36.3.5e Nov, 2012(preload8)]
 start: Sat May  9 00:13:31 2020 done: Sat May  9 00:13:31 2020
 Total Scan time:  0.160 Total Display time:  0.060

Function used was LALIGN [36.3.5e Nov, 2012(preload8)]
```

lalign output for NSP13 SARS COV vs. NSP13 SARS COV2

[EMBnet-Server] Date: Sat May 9 0:15:03 2020

```
# bin/lalign36 -E 10.0 -f -12 -g -2 6675.1.seq 6675.2.seq -J -K 3
LALIGN finds non-overlapping local alignments
 version 36.3.5e Nov, 2012(preload8)
Please cite:
 X. Huang and W. Miller (1991) Adv. Appl. Math. 12:373-381

Query: 6675.1.seq
  1>>>NSP13 SARS CoV 601 bp - 601 aa
Library: 6675.2.seq
      601 residues in     1 sequences

Statistics: (shuffled [500]) MLE statistics: Lambda= 0.1728;  K=0.02405
 statistics sampled from 1 (1) to 500 sequences
Threshold: E() < 10 score: 39
Algorithm: Smith-Waterman (SSE2, Michael Farrar 2006) (7.2 Nov 2010)
Parameters: BL50 matrix (15:-5), open/ext: -12/-2
 Scan time:  0.070

>>NSP13 SARS CoV-2 601 bp                               (601 aa)
 Waterman-Eggert score: 4038;  1011.9 bits; E(1) <  0
99.8% identity (100.0% similar) in 601 aa overlap (1-601:1-601)

               10        20        30        40        50        60
NSP13  AVGACVLCNSQTSLRCGACIRRPFLCCKCCYDHVISTSHKLVLSVNPYVCNAPGCDVTDV
       ::::::::::::::::::::::::::::::::::::::::::::::::::::::::::::
NSP13  AVGACVLCNSQTSLRCGACIRRPFLCCKCCYDHVISTSHKLVLSVNPYVCNAPGCDVTDV
               10        20        30        40        50        60

               70        80        90       100       110       120
NSP13  TQLYLGGMSYYCKSHKPPISFPLCANGQVFGLYKNTCVGSDNVTDFNAIATCDWTNAGDY
       ::::::::::::::::::::::::::::::::::::::::::::::::::::::::::::
NSP13  TQLYLGGMSYYCKSHKPPISFPLCANGQVFGLYKNTCVGSDNVTDFNAIATCDWTNAGDY
               70        80        90       100       110       120

              130       140       150       160       170       180
NSP13  ILANTCTERLKLFAAETLKATEETFKLSYGIATVREVLSDRELHLSWEVGKPRPPLNRNY
       ::::::::::::::::::::::::::::::::::::::::::::::::::::::::::::
NSP13  ILANTCTERLKLFAAETLKATEETFKLSYGIATVREVLSDRELHLSWEVGKPRPPLNRNY
              130       140       150       160       170       180

              190       200       210       220       230       240
NSP13  VFTGYRVTKNSKVQIGEYTFEKGDYGDAVVYRGTTTYKLNVGDYFVLTSHTVMPLSAPTL
       ::::::::::::::::::::::::::::::::::::::::::::::::::::::::::::
NSP13  VFTGYRVTKNSKVQIGEYTFEKGDYGDAVVYRGTTTYKLNVGDYFVLTSHTVMPLSAPTL
              190       200       210       220       230       240

              250       260       270       280       290       300
NSP13  VPQEHYVRITGLYPTLNISDEFSSNVANYQKVGMQKYSTLQGPPGTGKSHFAIGLALYYP
       ::::::::::::::::::::::::::::::::::::::::::::::::::::::::::::
NSP13  VPQEHYVRITGLYPTLNISDEFSSNVANYQKVGMQKYSTLQGPPGTGKSHFAIGLALYYP
```

```
              250       260       270       280       290       300

              310       320       330       340       350       360
NSP13  SARIVYTACSHAAVDALCEKALKYLPIDKCSRIIPARARVECFDKFKVNSTLEQYVFCTV
       ::::::::::::::::::::::::::::::::::::::::::::::::::::::::::::
NSP13  SARIVYTACSHAAVDALCEKALKYLPIDKCSRIIPARARVECFDKFKVNSTLEQYVFCTV
              310       320       330       340       350       360

              370       380       390       400       410       420
NSP13  NALPETTADIVVFDEISMATNYDLSVVNARLRAKHYVYIGDPAQLPAPRTLLTKGTLEPE
       ::::::::::::::::::::::::::::::::::::::::::::::::::::::::::::
NSP13  NALPETTADIVVFDEISMATNYDLSVVNARLRAKHYVYIGDPAQLPAPRTLLTKGTLEPE
              370       380       390       400       410       420

              430       440       450       460       470       480
NSP13  YFNSVCRLMKTIGPDMFLGTCRRCPAEIVDTVSALVYDNKLKAHKDKSAQCFKMFYKGVI
       ::::::::::::::::::::::::::::::::::::::::::::::::::::::::::::
NSP13  YFNSVCRLMKTIGPDMFLGTCRRCPAEIVDTVSALVYDNKLKAHKDKSAQCFKMFYKGVI
              430       440       450       460       470       480

              490       500       510       520       530       540
NSP13  THDVSSAINRPQIGVVREFLTRNPAWRKAVFISPYNSQNAVASKILGLPTQTVDSSQGSE
       ::::::::::::::::::::::::::::::::::::::::::::::::::::::::::::
NSP13  THDVSSAINRPQIGVVREFLTRNPAWRKAVFISPYNSQNAVASKILGLPTQTVDSSQGSE
              490       500       510       520       530       540

              550       560       570       580       590       600
NSP13  YDYVIFTQTTETAHSCNVNRFNVAITRAKIGILCIMSDRDLYDKLQFTSLEIPRRNVATL
       :::::::::::::::::::::::::::::.::::::::::::::::::::::::::::::
NSP13  YDYVIFTQTTETAHSCNVNRFNVAITRAKVGILCIMSDRDLYDKLQFTSLEIPRRNVATL
              550       560       570       580       590       600


NSP13  Q
       :
NSP13  Q


>--
 Waterman-Eggert score: 42;  15.8 bits; E(1) <  1
42.9% identity (78.6% similar) in 14 aa overlap (211-224:185-198)

              220
NSP13  YRGTTTYKLNVGDY
       :: : . :...:.:
NSP13  YRVTKNSKVQIGEY
          190

>--
 Waterman-Eggert score: 42;  15.8 bits; E(1) <  1
42.9% identity (78.6% similar) in 14 aa overlap (185-198:211-224)

          190
NSP13  YRVTKNSKVQIGEY
       :: : . :...:.:
NSP13  YRGTTTYKLNVGDY
              220
```

```
601 residues in 1 query   sequences
601 residues in 1 library sequences
 Scomplib [36.3.5e Nov, 2012(preload8)]
 start: Sat May  9 00:15:04 2020 done: Sat May  9 00:15:04 2020
 Total Scan time:  0.070 Total Display time:  0.030

Function used was LALIGN [36.3.5e Nov, 2012(preload8)]
```

lalign output for NSP14 SARS COV vs. NSP14 SARS COV2

[EMBnet-Server] Date: Sat May 9 0:16:29 2020

```
# bin/lalign36 -E 10.0 -f -12 -g -2 6674.1.seq 6674.2.seq -J -K 3
LALIGN finds non-overlapping local alignments
 version 36.3.5e Nov, 2012(preload8)
Please cite:
 X. Huang and W. Miller (1991) Adv. Appl. Math. 12:373-381

Query: 6674.1.seq
  1>>>NSP14 SARS CoV 527 bp - 527 aa
Library: 6674.2.seq
      527 residues in     1 sequences

Statistics: (shuffled [500]) MLE statistics: Lambda= 0.1674;  K=0.0192
 statistics sampled from 1 (1) to 500 sequences
Threshold: E() < 10 score: 38
Algorithm: Smith-Waterman (SSE2, Michael Farrar 2006) (7.2 Nov 2010)
Parameters: BL50 matrix (15:-5), open/ext: -12/-2
 Scan time:  0.060

>>NSP14 SARS CoV-2 527 bp                              (527 aa)
 Waterman-Eggert score: 3592;  873.2 bits; E(1) <  0
95.1% identity (99.1% similar) in 527 aa overlap (1-527:1-527)

               10        20        30        40        50        60
NSP14  AENVTGLFKDCSKIITGLHPTQAPTHLSVDIKFKTEGLCVDIPGIPKDMTYRRLISMMGF
       :::::::::::::.::::::::::::::::: ::::::::::::::::::::::::::::
NSP14  AENVTGLFKDCSKVITGLHPTQAPTHLSVDTKFKTEGLCVDIPGIPKDMTYRRLISMMGF
               10        20        30        40        50        60

               70        80        90       100       110       120
NSP14  KMNYQVNGYPNMFITREEAIRHVRAWIGFDVEGCHATRDAVGTNLPLQLGFSTGVNLVAV
       :::::::::::::::::::::::::::::::::::::::::.::::::::::::::::::
NSP14  KMNYQVNGYPNMFITREEAIRHVRAWIGFDVEGCHATREAVGTNLPLQLGFSTGVNLVAV
               70        80        90       100       110       120

              130       140       150       160       170       180
NSP14  PTGYVDTENNTEFTRVNAKPPPGDQFKHLIPLMYKGLPWNVVRIKIVQMLSDTLKGLSDR
       ::::::: :::.:.::.::::::::::::::::::::::::::::::::::::::.::::
NSP14  PTGYVDTPNNTDFSRVSAKPPPGDQFKHLIPLMYKGLPWNVVRIKIVQMLSDTLKNLSDR
              130       140       150       160       170       180

              190       200       210       220       230       240
NSP14  VVFVLWAHGFELTSMKYFVKIGPERTCCLCDKRATCFSTSSDTYACWNHSVGFDYVYNPF
       :::::::::::::::::::::::::::::::.:::::::.:::::::.::.:::::::::
NSP14  VVFVLWAHGFELTSMKYFVKIGPERTCCLCDRRATCFSTASDTYACWHHSIGFDYVYNPF
              190       200       210       220       230       240

              250       260       270       280       290       300
NSP14  MIDVQQWGFTGNLQSNHDQHCQVHGNAHVASCDAIMTRCLAVHECFVKRVDWSVEYPIIG
       :::::::::::::::::: .::::::::::::::::::::::::::::::::..::::::
NSP14  MIDVQQWGFTGNLQSNHDLYCQVHGNAHVASCDAIMTRCLAVHECFVKRVDWTIEYPIIG
```

```
          250       260       270       280       290       300

          310       320       330       340       350       360
NSP14  DELRVNSACRKVQHMVVKSALLADKFPVLHDIGNPKAIKCVPQAEVEWKFYDAQPCSDKA
       :::..:.::::::::::::.:::::::::::::::::::::::::.:::::::::::::::
NSP14  DELKINAACRKVQHMVVKAALLADKFPVLHDIGNPKAIKCVPQADVEWKFYDAQPCSDKA
          310       320       330       340       350       360

          370       380       390       400       410       420
NSP14  YKIEELFYSYATHHDKFTDGVCLFWNCNVDRYPANAIVCRFDTRVLSNLNLPGCDGGSLY
       ::::::::::::: ::::::::::::::::::::::.:::::::::::::::::::::::
NSP14  YKIEELFYSYATHSDKFTDGVCLFWNCNVDRYPANSIVCRFDTRVLSNLNLPGCDGGSLY
          370       380       390       400       410       420

          430       440       450       460       470       480
NSP14  VNKHAFHTPAFDKSAFTNLKQLPFFYYSDSPCESHGKQVVSDIDYVPLKSATCITRCNLG
       ::::::::::::::::.:::::::::::::::::::::::::::::::::::::::::::
NSP14  VNKHAFHTPAFDKSAFVNLKQLPFFYYSDSPCESHGKQVVSDIDYVPLKSATCITRCNLG
          430       440       450       460       470       480

          490       500       510       520
NSP14  GAVCRHHANEYRQYLDAYNMMISAGFSLWIYKQFDTYNLWNTFTRLQ
       :::::::::::: :::::::::::::::::.::::::::::::::::
NSP14  GAVCRHHANEYRLYLDAYNMMISAGFSLWVYKQFDTYNLWNTFTRLQ
          490       500       510       520

>--
 Waterman-Eggert score: 53;  18.5 bits; E(1) <  0.53
34.9% identity (62.8% similar) in 43 aa overlap (113-153:16-58)

        120        130       140        150
NSP14  TGVNLVAVPTGY-VDTENNTEFTRVNAKPPPGDQ-FKHLIPLM
       ::.. . .::   :::. .::   :.    : :. ...:: .:
NSP14  TGLHPTQAPTHLSVDTKFKTEGLCVDIPGIPKDMTYRRLISMM
         20        30        40        50

>--
 Waterman-Eggert score: 47;  17.1 bits; E(1) <  0.87
22.0% identity (52.0% similar) in 50 aa overlap (469-517:212-260)

    470       480       490       500        510
NSP14  KSATCITRCNLGGAVCRHHANEYRQYLDAYNMMISA-GFSLWIYKQFDTY
       . :::..  .    : : ::.  .     . . . ..  ::.  . .. : :
NSP14  RRATCFSTASDTYA-CWHHSIGFDYVYNPFMIDVQQWGFTGNLQSNHDLY
            220       230       240       250       260



527 residues in 1 query   sequences
527 residues in 1 library sequences
 Scomplib [36.3.5e Nov, 2012(preload8)]
 start: Sat May  9 00:16:30 2020 done: Sat May  9 00:16:30 2020
 Total Scan time:  0.060 Total Display time:  0.020

Function used was LALIGN [36.3.5e Nov, 2012(preload8)]
```

lalign output for NSP15 SARS COV vs. NSP15 SARS COV2

[EMBnet-Server] Date: Sat May 9 0:17:55 2020

```
# bin/lalign36 -E 10.0 -f -12 -g -2 7450.1.seq 7450.2.seq -J -K 3
LALIGN finds non-overlapping local alignments
 version 36.3.5e Nov, 2012(preload8)
Please cite:
 X. Huang and W. Miller (1991) Adv. Appl. Math. 12:373-381

Query: 7450.1.seq
  1>>>NSP15 SARS CoV 346 bp - 346 aa
Library: 7450.2.seq
      346 residues in     1 sequences

Statistics: (shuffled [500]) MLE statistics: Lambda= 0.1879;  K=0.03691
 statistics sampled from 1 (1) to 500 sequences
Threshold: E() < 10 score: 32
Algorithm: Smith-Waterman (SSE2, Michael Farrar 2006) (7.2 Nov 2010)
Parameters: BL50 matrix (15:-5), open/ext: -12/-2
 Scan time:  0.020

>>NSP15 SARS CoV-2 346 bp                                 (346 aa)
 Waterman-Eggert score: 2086;  570.1 bits; E(1) <  2.9e-167
88.7% identity (97.7% similar) in 346 aa overlap (1-346:1-346)

              10        20        30        40        50        60
NSP15  SLENVAYNVVNKGHFDGHAGEAPVSIINNAVYTKVDGIDVEIFENKTTLPVNVAFELWAK
       ::::::.::::::::::. ::.:::::::.:::::::.:::.::::::::::::::::::
NSP15  SLENVAFNVVNKGHFDGQQGEVPVSIINNTVYTKVDGVDVELFENKTTLPVNVAFELWAK
              10        20        30        40        50        60

              70        80        90       100       110       120
NSP15  RNIKPVPEIKILNNLGVDIAANTVIWDYKREAPAHVSTIGVCTMTDIAKKPTESACSSLT
       ::::::::.:::::::::::::::::::::.::::.::::::.::::::::::. :. ::
NSP15  RNIKPVPEVKILNNLGVDIAANTVIWDYKRDAPAHISTIGVCSMTDIAKKPTETICAPLT
              70        80        90       100       110       120

             130       140       150       160       170       180
NSP15  VLFDGRVEGQVDLFRNARNGVLITEGSVKGLTPSKGPAQASVNGVTLIGESVKTQFNYFK
       :.:::::.::::::::::::::::::::::: :: :: :::.::::::::.:::::::.:
NSP15  VFFDGRVDGQVDLFRNARNGVLITEGSVKGLQPSVGPKQASLNGVTLIGEAVKTQFNYYK
             130       140       150       160       170       180

             190       200       210       220       230       240
NSP15  KVDGIIQQLPETYFTQSRDLEDFKPRSQMETDFLELAMDEFIQRYKLEGYAFEHIVYGDF
       ::::..::::::::::::.:..:::::::: :::::::::::.:::::::::::::::::
NSP15  KVDGVVQQLPETYFTQSRNLQEFKPRSQMEIDFLELAMDEFIERYKLEGYAFEHIVYGDF
             190       200       210       220       230       240

             250       260       270       280       290       300
NSP15  SHGQLGGLHLMIGLAKRSQDSPLKLEDFIPMDSTVKNYFITDAQTGSSKCVCSVIDLLLD
       ::.:::::::.:::::: ..::..::::::::::::::::::::::::::::::::::::
NSP15  SHSQLGGLHLLIGLAKRFKESPFELEDFIPMDSTVKNYFITDAQTGSSKCVCSVIDLLLD
```

```
              250       260       270       280       290       300

              310       320       330       340
NSP15  DFVEIIKSQDLSVISKVVKVTIDYAEISFMLWCKDGHVETFYPKLQ
       :::::::::::::.::::::::::.:::::::::::::::::::::
NSP15  DFVEIIKSQDLSVVSKVVKVTIDYTEISFMLWCKDGHVETFYPKLQ
              310       320       330       340

>--
 Waterman-Eggert score: 54;  19.4 bits; E(1) <  0.16
31.2% identity (56.2% similar) in 32 aa overlap (97-128:273-304)

        100       110       120
NSP15  STIGVCTMTDIAKKPTESACSSLTVLFDGRVE
       ::.    .::     .. .:: . .:.:  ::
NSP15  STVKNYFITDAQTGSSKCVCSVIDLLLDDFVE
            280       290       300

>--
 Waterman-Eggert score: 48;  17.8 bits; E(1) <  0.41
25.6% identity (64.1% similar) in 39 aa overlap (167-205:236-270)

        170       180       190       200
NSP15  LIGESVKTQFNYFKKVDGIIQQLPETYFTQSRDLEDFKP
       . :.  ..:.. .. . :. ... :. :    .:::: :
NSP15  VYGDFSHSQLGGLHLLIGLAKRFKESPF----ELEDFIP
         240       250       260           270



346 residues in 1 query   sequences
346 residues in 1 library sequences
 Scomplib [36.3.5e Nov, 2012(preload8)]
 start: Sat May  9 00:17:56 2020 done: Sat May  9 00:17:56 2020
 Total Scan time:  0.020 Total Display time:  0.020

Function used was LALIGN [36.3.5e Nov, 2012(preload8)]
```

Readseq version 2.1.30 (12-May-2010) Readseq version 2.1.30 (12-May-2010)

lalign output for NSP16 SARS COV vs. NSP16 SARS COV2

[EMBnet-Server] Date: Sat May 9 14:40:56 2020

```
# bin/lalign36 -E 10.0 -f -12 -g -2 16523.1.seq 16523.2.seq -J -K 3
LALIGN finds non-overlapping local alignments
 version 36.3.5e Nov, 2012(preload8)
Please cite:
 X. Huang and W. Miller (1991) Adv. Appl. Math. 12:373-381

Query: 16523.1.seq
  1>>>NSP16 SARS CoV 298 bp - 298 aa
Library: 16523.2.seq
      298 residues in     1 sequences

Statistics: (shuffled [500]) MLE statistics: Lambda= 0.1873;  K=0.0366
 statistics sampled from 1 (1) to 500 sequences
Threshold: E() < 10 score: 31
Algorithm: Smith-Waterman (SSE2, Michael Farrar 2006) (7.2 Nov 2010)
Parameters: BL50 matrix (15:-5), open/ext: -12/-2
 Scan time:  0.020

>>NSP16 SARS CoV-2 298 bp                                 (298 aa)
 Waterman-Eggert score: 1902;  518.7 bits; E(1) <  6.5e-152
93.3% identity (99.0% similar) in 298 aa overlap (1-298:1-298)

               10        20        30        40        50        60
NSP16  ASQAWQPGVAMPNLYKMQRMLLEKCDLQNYGENAVIPKGIMMNVAKYTQLCQYLNTLTLA
       .::::::::::::::::::::::::::::::..:..::::::::::::::::::::::::
NSP16  SSQAWQPGVAMPNLYKMQRMLLEKCDLQNYGDSATLPKGIMMNVAKYTQLCQYLNTLTLA
               10        20        30        40        50        60

               70        80        90       100       110       120
NSP16  VPYNMRVIHFGAGSDKGVAPGTAVLRQWLPTGTLLVDSDLNDFVSDADSTLIGDCATVHT
       ::::::::::::::::::::::::::::::::::::::::::::::::::::::::::::
NSP16  VPYNMRVIHFGAGSDKGVAPGTAVLRQWLPTGTLLVDSDLNDFVSDADSTLIGDCATVHT
               70        80        90       100       110       120

              130       140       150       160       170       180
NSP16  ANKWDLIISDMYDPRTKHVTKENDSKEGFFTYLCGFIKQKLALGGSIAVKITEHSWNADL
       ::::::::::::::.::.::::::::::::::.::::.::::::::.:.:::::::::::
NSP16  ANKWDLIISDMYDPKTKNVTKENDSKEGFFTYICGFIQQKLALGGSVAIKITEHSWNADL
              130       140       150       160       170       180

              190       200       210       220       230       240
NSP16  YKLMGHFSWWTAFVTNVNASSSEAFLIGANYLGKPKEQIDGYTMHANYIFWRNTNPIQLS
       :::::::.:::::::::::::::::::: ::::::.::::::.:::::::::::::::::
NSP16  YKLMGHFAWWTAFVTNVNASSSEAFLIGCNYLGKPREQIDGYVMHANYIFWRNTNPIQLS
              190       200       210       220       230       240

              250       260       270       280       290
NSP16  SYSLFDMSKFPLKLRGTAVMSLKENQINDMIYSLLEKGRLIIRENNRVVVSSDILVNN
```

```
       ::::::::::::::::::::::::.:::::: ::: ::::::::::::.:::.::::
NSP16  SYSLFDMSKFPLKLRGTAVMSLKEGQINDMILSLLSKGRLIIRENNRVVISSDVLVNN
              250       260       270       280       290

>--
 Waterman-Eggert score: 42;  16.1 bits; E(1) <  0.71
29.2% identity (75.0% similar) in 24 aa overlap (252-275:161-184)

             260       270
NSP16  LKLRGTAVMSLKENQINDMIYSLL
       : : :...... :.. :  .:.:.
NSP16  LALGGSVAIKITEHSWNADLYKLM
              170       180

>--
 Waterman-Eggert score: 36;  14.5 bits; E(1) <  0.98
26.5% identity (58.8% similar) in 34 aa overlap (74-107:1-34)

            80        90       100
NSP16  SDKGVAPGTAVLRQWLPTGTLLVDSDLNDFVSDA
       :...  ::.:.   .     ::   ::... ..:
NSP16  SSQAWQPGVAMPNLYKMQRMLLEKCDLQNYGDSA
               10        20        30



298 residues in 1 query   sequences
298 residues in 1 library sequences
 Scomplib [36.3.5e Nov, 2012(preload8)]
 start: Sat May  9 14:40:57 2020 done: Sat May  9 14:40:57 2020
 Total Scan time:  0.020 Total Display time:  0.010

Function used was LALIGN [36.3.5e Nov, 2012(preload8)]
```

lalign output for S PROTEIN SARS COV vs. S PROTEIN SARS COV2

[EMBnet-Server] Date: Sat May 9 0:27:26 2020

```
# bin/lalign36 -E 10.0 -f -12 -g -2 5148.1.seq 5148.2.seq -J -K 3
LALIGN finds non-overlapping local alignments
 version 36.3.5e Nov, 2012(preload8)
Please cite:
 X. Huang and W. Miller (1991) Adv. Appl. Math. 12:373-381

Query: 5148.1.seq
  1>>>S Protein SARS CoV 1255 bp - 1255 aa
Library: 5148.2.seq
     1273 residues in     1 sequences

Statistics: (shuffled [500]) MLE statistics: Lambda= 0.1533;  K=0.008311
 statistics sampled from 1 (1) to 500 sequences
Threshold: E() < 10 score: 47
Algorithm: Smith-Waterman (SSE2, Michael Farrar 2006) (7.2 Nov 2010)
Parameters: BL50 matrix (15:-5), open/ext: -12/-2
 Scan time:  0.250

>>S Protein SARS CoV-2 1273 bp                          (1273 aa)
 Waterman-Eggert score: 6655;  1478.7 bits; E(1) <  0
76.0% identity (91.5% similar) in 1277 aa overlap (1-1255:1-1273)

               10        20        30        40        50        60
S      MFIFLLFLTLTSGSDLDRCTTFDDVQAPNYTQHTSSMRGVYYPDEIFRSDTLYLTQDLFL
       ::.::..: :.:.. ..  :  .    : ::.  :  :::::::.::::..:. ::::::
S      MFVFLVLLPLVSSQCVNLTTRTQ--LPPAYTN--SFTRGVYYPDKVFRSSVLHSTQDLFL
               10        20          30          40        50

               70               80        90       100       110
S      PFYSNVTGFHTIN-------HTFGNPVIPFKDGIYFAATEKSNVVRGWVFGSTMNNKSQS
       ::.:::: ::.:.       . : :::.::.::.:::.:::::..:::.::.:...:.::
S      PFFSNVTWFHAIHVSGTNGTKRFDNPVLPFNDGVYFASTEKSNIIRGWIFGTTLDSKTQS
          60        70        80        90       100       110

          120       130       140           150       160
S      VIIINNSTNVVIRACNFELCDNPFFAV----SKPMGTQTHTMIFDNAFNCTFEYISDAFS
       ..:.::.:::::..:.:..:..::..:    ..    ...  ....: ::::::.:. :
S      LLIVNNATNVVIKVCEFQFCNDPFLGVYYHKNNKSWMESEFRVYSSANNCTFEYVSQPFL
        120       130       140       150       160       170

    170       180       190       200       210       220
S      LDVSEKSGNFKHLREFVFKNKDGFLYVYKGYQPIDVVRDLPSGFNTLKPIFKLPLGINIT
       .:.  :.::::.:::::::: ::.. .:. . ::..:::::.::..:.:.  ::.:::::
S      MDLEGKQGNFKNLREFVFKNIDGYFKIYSKHTPINLVRDLPQGFSALEPLVDLPIGINIT
        180       190       200       210       220       230

    230           240         250       260       270       280
S      NFRAIL----TAFSPAQDI--WGTSAAAYFVGYLKPTTFMLKYDENGTITDAVDCSQNPL
        :...:    . ..:...   : ..::::.::::.: ::.:::.:::::::::::. .::
S      RFQTLLALHRSYLTPGDSSSGWTAGAAAYYVGYLQPRTFLLKYNENGTITDAVDCALDPL
```

```
        240       250       260       270       280       290

           290       300       310       320       330       340
S      AELKCSVKSFEIDKGIYQTSNFRVVPSGDVVRFPNITNLCPFGEVFNATKFPSVYAWERK
       .: ::..::: ..::::::::::: :. ..:::::::::::::::::::.: :::::.::
S      SETKCTLKSFTVEKGIYQTSNFRVQPTESIVRFPNITNLCPFGEVFNATRFASVYAWNRK
        300       310       320       330       340       350

           350       360       370       380       390       400
S      KISNCVADYSVLYNSTFFSTFKCYGVSATKLNDLCFSNVYADSFVVKGDDVRQIAPGQTG
       .::::::::::::::. :::::::::: ::::::::.::::::::..::.::::::::::
S      RISNCVADYSVLYNSASFSTFKCYGVSPTKLNDLCFTNVYADSFVIRGDEVRQIAPGQTG
        360       370       380       390       400       410

           410       420       430       440       450       460
S      VIADYNYKLPDDFMGCVLAWNTRNIDATSTGNYNYKYRYLRHGKLRPFERDISNVPFSPD
        :::::::::::: :::.:::. :.:.   ::::: :: .:...:.:::::::.  ..  
S      KIADYNYKLPDDFTGCVIAWNSNNLDSKVGGNYNYLYRLFRKSNLKPFERDISTEIYQAG
        420       430       440       450       460       470

            470       480       490       500       510       520
S      GKPCTP-PALNCYWPLNDYGFYTTTGIGYQPYRVVVLSFELLNAPATVCGPKLSTDLIKN
       . ::.   ..:::.::..:::  :.:.:::::::::::::::.::::::::: ::.:.::
S      STPCNGVEGFNCYFPLQSYGFQPTNGVGYQPYRVVVLSFELLHAPATVCGPKKSTNLVKN
        480       490       500       510       520       530

            530       540       550       560       570       580
S      QCVNFNFNGLTGTGVLTPSSKRFQPFQQFGRDVSDFTDSVRDPKTSEILDISPCAFGGVS
       .:::::::::::::::: :.:.: ::::::::..: ::.::::.: :::::.::.:::::
S      KCVNFNFNGLTGTGVLTESNKKFLPFQQFGRDIADTTDAVRDPQTLEILDITPCSFGGVS
        540       550       560       570       580       590

            590       600       610       620       630       640
S      VITPGTNASSEVAVLYQDVNCTDVSTAIHADQLTPAWRIYSTGNNVFQTQAGCLIGAEHV
       :::::::.:..:::::::::::.: .:::::::::.::.::::.:::::.::::::::::
S      VITPGTNTSNQVAVLYQDVNCTEVPVAIHADQLTPTWRVYSTGSNVFQTRAGCLIGAEHV
        600       610       620       630       640       650

            650       660           670       680       690
S      DTSYECDIPIGAGICASYHTVS----LLRSTSQKSIVAYTMSLGADSSIAYSNNTIAIPT
       ..::::::::::::::::.: .      ::....::.::::::::..:.:::::.:::::
S      NNSYECDIPIGAGICASYQTQTNSPRRARSVASQSIIAYTMSLGAENSVAYSNNSIAIPT
        660       670       680       690       700       710

      700       710       720       730       740       750
S      NFSISITTEVMPVSMAKTSVDCNMYICGDSTECANLLLQYGSFCTQLNRALSGIAAEQDR
       ::.::.:::..::::.::::::.::::::::::.:::::::::::::::::.:::.:::.
S      NFTISVTTEILPVSMTKTSVDCTMYICGDSTECSNLLLQYGSFCTQLNRALTGIAVEQDK
        720       730       740       750       760       770

      760       770       780       790       800       810
S      NTREVFAQVKQMYKTPTLKYFGGFNFSQILPDPLKPTKRSFIEDLLFNKVTLADAGFMKQ
       ::.::::::::.:::: .: ::::::::::::: ::.::::::::::::::::::::.::
S      NTQEVFAQVKQIYKTPPIKDFGGFNFSQILPDPSKPSKRSFIEDLLFNKVTLADAGFIKQ
        780       790       800       810       820       830

      820       830       840       850       860       870
```

```
S      YGECLGDINARDLICAQKFNGLTVLPPLLTDDMIAAYTAALVSGTATAGWTFGAGAALQI
       ::.::::: ::::::::::::::::::::::.::: ::.::..:: :.::::::::::::
S      YGDCLGDIAARDLICAQKFNGLTVLPPLLTDEMIAQYTSALLAGTITSGWTFGAGAALQI
        840       850       860       870       880       890

      880       890       900       910       920       930
S      PFAMQMAYRFNGIGVTQNVLYENQKQIANQFNKAISQIQESLTTTSTALGKLQDVVNQNA
       :::::::::::::::::::::::: ::::::.::..::.::..:.:::::::::::::::
S      PFAMQMAYRFNGIGVTQNVLYENQKLIANQFNSAIGKIQDSLSSTASALGKLQDVVNQNA
        900       910       920       930       940       950

      940       950       960       970       980       990
S      QALNTLVKQLSSNFGAISSVLNDILSRLDKVEAEVQIDRLITGRLQSLQTYVTQQLIRAA
       ::::::::::::::::::::::::::::::::::::::::::::::::::::::::::::
S      QALNTLVKQLSSNFGAISSVLNDILSRLDKVEAEVQIDRLITGRLQSLQTYVTQQLIRAA
        960       970       980       990      1000      1010

     1000      1010      1020      1030      1040      1050
S      EIRASANLAATKMSECVLGQSKRVDFCGKGYHLMSFPQAAPHGVVFLHVTYVPSQERNFT
       :::::::::::::::::::::::::::::::::::::.::::::::::::::.::.:::
S      EIRASANLAATKMSECVLGQSKRVDFCGKGYHLMSFPQSAPHGVVFLHVTYVPAQEKNFT
       1020      1030      1040      1050      1060      1070

     1060      1070      1080      1090      1100      1110
S      TAPAICHEGKAYFPREGVFVFNGTSWFITQRNFFSPQIITTDNTFVSGNCDVVIGIINNT
       :::::::.:::.:::::::: ::: ::.:::::. ::::::::::::::::::::::.:::
S      TAPAICHDGKAHFPREGVFVSNGTHWFVTQRNFYEPQIITTDNTFVSGNCDVVIGIVNNT
       1080      1090      1100      1110      1120      1130

     1120      1130      1140      1150      1160      1170
S      VYDPLQPELDSFKEELDKYFKNHTSPDVDLGDISGINASVVNIQKEIDRLNEVAKNLNES
       ::::::::::::::::::::::::::::::::::::::::::::::::::::::::::::
S      VYDPLQPELDSFKEELDKYFKNHTSPDVDLGDISGINASVVNIQKEIDRLNEVAKNLNES
       1140      1150      1160      1170      1180      1190

     1180      1190      1200      1210      1220      1230
S      LIDLQELGKYEQYIKWPWYVWLGFIAGLIAIVMVTILLCCMTSCCSCLKGACSCGSCCKF
       ::::::::::::::::::::.:::::::::::::::::.:::::::::::::: :::::::::
S      LIDLQELGKYEQYIKWPWYIWLGFIAGLIAIVMVTIMLCCMTSCCSCLKGCCSCGSCCKF
       1200      1210      1220      1230      1240      1250

     1240      1250
S      DEDDSEPVLKGVKLHYT
       :::::::::::::::::
S      DEDDSEPVLKGVKLHYT
       1260      1270
```

>--
 Waterman-Eggert score: 91; 27.0 bits; E(1) < 0.012
25.4% identity (55.3% similar) in 114 aa overlap (1121-1230:1146-1253)

```
             1130      1140      1150      1160      1170
S      DPLQPELDS-FKEELDKYFKNHTSPDVDLGDISGINASVVNIQKEIDRLNEVAKNLNESL
       : .. :::. ::.. .           .. ... .:.  .  ...    :::   .:.: :
S      DSFKEELDKYFKNHTSPDVDLGDISGIN-ASVVNIQKEIDRLNEVAKNLNESLIDLQE-L
        1150      1160      1170       1180      1190      1200
```

```
    1180      1190      1200      1210         1220      1230
S      IDLQELGKYEQYIKWPWYVWLGFIAGLIAIVMVTILL-CC--MTSCCSCLKGAC
          ..  :.  :: :  ..  :.:: ... .:.  .  ::  . .::::  :.:
S      GKYEQYIKWPWYI-WLGFI-AGLIAIVMVTIMLCCMTSCCSCLKGCCSC--GSC
          1210       1220       1230      1240      1250

>--
 Waterman-Eggert score: 88;  26.4 bits; E(1) <  0.018
19.1% identity (51.2% similar) in 330 aa overlap (924-1236:928-1248)

          930       940       950       960       970
S      STALGKLQDVVNQNAQALNTLVKQLSSNFGAISSVLNDILSRLDKVEAEVQ--IDRL--I
        ..:.::.:: ....:.::. :   ...:  :........ : .  . . ..  ..::  .
S      NSAIGKIQDSLSSTASALGKLQDVVNQNAQALNTLVKQLSSNFGAISSVLNDILSRLDKV
       930       940       950       960       970       980

     980       990      1000      1010      1020       1030
S      TGRLQSLQTYVTQQLIRAAEIRASANLAATKMSECVLGQSKRVDFCGKGY-HLMSFPQAA
        ...: ..  .: .:         ..  . :...    .    . ... :  :  . ..:   .
S      EAEVQ-IDRLITGRLQSLQTYVTQQLIRAAEIRASANLAATKMSECVLGQSKRVDFCGKG
       990       1000      1010      1020      1030      1040

     1040      1050      1060      1070      1080      1090
S      PHGVVFLHVTYVPSQERNFTTAPAICHEGKAYFPREGVFVFNGTSWFITQRNFFSP---Q
        : . : . .          . : .::      .  :      ..  .: . :  .  : :
S      YHLMSFPQSAPHGVVFLHVTYVPA----QEKNFTTAPAICHDGKAHFPREGVFVSNGTHW
       1050      1060      1070             1080      1090      1100

        1100          1110      1120      1130      1140      1150
S      IITTDNTF----VSGNCDVVIGIINNTVYDPLQPELDSFKEELDKYFKNHTSPDVDLGDI
        ..:  : .      .. .    : :  . ..     .   : .. :::. ::.. .
S      FVTQRNFYEPQIITTDNTFVSGNCDVVIGIVNNTVYDPLQPELDS-FKEELDKYFKNHTS
           1110      1120      1130      1140       1150      1160

           1160      1170      1180       1190       1200
S      SGIN-ASVVNIQKEIDRLNEVAKNLNESLIDLQE-LGKYEQYIKWPWYV-WLGFI-AGLI
         .. ... .:.   .   ...      :::   .:.: :    ..   :.   :. :   .:   :.:
S      PDVDLGDISGINASVVNIQKEIDRLNEVAKNLNESLIDLQELGKYEQYIKWPWYIWLGFI
            1170      1180      1190      1200      1210      1220

      1210      1220      1230
S      AIVMVTILLCCMTSCCSCLKGACSC-GSCC
       : ... ...  :  ::  . . :::  .::
S      AGLIAIVMVTIML-CC--MTSCCSCLKGCC
            1230         1240
```

1255 residues in 1 query   sequences
1273 residues in 1 library sequences
 Scomplib [36.3.5e Nov, 2012(preload8)]
 start: Sat May 9 00:27:27 2020 done: Sat May 9 00:27:27 2020
 Total Scan time: 0.250 Total Display time: 0.100

Function used was LALIGN [36.3.5e Nov, 2012(preload8)]

lalign output for ORF3A SARS COV vs. ORF3A SARS COV2

[EMBnet-Server] Date: Sat May 9 0:29:57 2020

```
# bin/lalign36 -E 10.0 -f -12 -g -2 7505.1.seq 7505.2.seq -J -K 3
LALIGN finds non-overlapping local alignments
 version 36.3.5e Nov, 2012(preload8)
Please cite:
 X. Huang and W. Miller (1991) Adv. Appl. Math. 12:373-381

Query: 7505.1.seq
  1>>>ORF3a SARS CoV 274 bp - 274 aa
Library: 7505.2.seq
      275 residues in     1 sequences

Statistics: (shuffled [500]) MLE statistics: Lambda= 0.1964;  K=0.05612
 statistics sampled from 1 (1) to 500 sequences
Threshold: E() < 10 score: 31
Algorithm: Smith-Waterman (SSE2, Michael Farrar 2006) (7.2 Nov 2010)
Parameters: BL50 matrix (15:-5), open/ext: -12/-2
 Scan time:  0.020

>>ORF3a SARS CoV-2 275 bp                                 (275 aa)
 Waterman-Eggert score: 1381;  395.4 bits; E(1) <  7.1e-115
72.4% identity (90.2% similar) in 275 aa overlap (1-274:1-275)

               10        20        30        40        50        60
ORF3a  MDLFMRFFTLRSITAQPVKIDNASPASTVHATATIPLQASLPFGWLVIGVAFLAVFQSAT
       ::::::.::. ..: .  .: .:.:.. :.::::::.:::::::::..:::.:::::::.
ORF3a  MDLFMRIFTIGTVTLKQGEIKDATPSDFVRATATIPIQASLPFGWLIVGVALLAVFQSAS
               10        20        30        40        50        60

               70        80        90       100       110       120
ORF3a  KIIALNKRWQLALYKGFQFICNLLLLFVTIYSHLLLVAAGMEAQFLYLYALIYFLQCINA
       :::.:.::::::: :: .:.:::::::::.::::::::::.:: :::::::.:::: :: 
ORF3a  KIITLKKRWQLALSKGVHFVCNLLLLFVTVYSHLLLVAAGLEAPFLYLYALVYFLQSINF
               70        80        90       100       110       120

              130       140       150       160       170       180
ORF3a  CRIIMRCWLCWKCKSKNPLLYDANYFVCWHTHNYDYCIPYNSVTDTIVVTEGDGISTPKL
        ::::: ::::::.::::::::::::.::::. :::::::::::..::.: ::: ..:  
ORF3a  VRIIMRLWLCWKCRSKNPLLYDANYFLCWHTNCYDYCIPYNSVTSSIVITSGDGTTSPIS
              130       140       150       160       170       180

              190       200       210       220       230       240
ORF3a  KEDYQIGGYSEDRHSGVKDYVVVHGYFTEVYYQLESTQITTDTGIENATFFIFNKLVKDP
       ..:::::::.:  .::::: ::.:.:::  :::: :::..::::.:..::::.::.: .:
ORF3a  EHDYQIGGYTEKWESGVKDCVVLHSYFTSDYYQLYSTQLSTDTGVEHVTFFIYNKIVDEP
              190       200       210       220       230       240

                250       260       270
ORF3a  P-NVQIHTIDGSSGVANPAMDPIYDEPTTTTSVPL
         .::::::::::::.::.:.::::::::::::::
ORF3a  EEHVQIHTIDGSSGVVNPVMEPIYDEPTTTTSVPL
```

```
              250       260       270

>--
 Waterman-Eggert score: 41;  15.8 bits; E(1) <  0.74
28.9% identity (46.7% similar) in 45 aa overlap (42-81:104-148)

              50        60        70             80
ORF3a  PFGWLVIGVAFLAVFQSATKIIALNKRWQLA-----LYKGFQFIC
       :: .:   : ::  .. .  :. :   :.       :: .  :.:
ORF3a  PFLYLYALVYFLQSINFVRIIMRLWLCWKCRSKNPLLYDANYFLC
           110       120       130       140

>--
 Waterman-Eggert score: 38;  14.9 bits; E(1) <  0.91
75.0% identity (75.0% similar) in 4 aa overlap (127-130:130-133)

        130
ORF3a  CWLC
       :: :
ORF3a  CWKC
     130



274 residues in 1 query   sequences
275 residues in 1 library sequences
 Scomplib [36.3.5e Nov, 2012(preload8)]
 start: Sat May  9 00:29:57 2020 done: Sat May  9 00:29:57 2020
 Total Scan time:  0.020 Total Display time:  0.000

Function used was LALIGN [36.3.5e Nov, 2012(preload8)]
```

lalign output for E PROTEIN SARS COV vs. E PROTEIN SARS COV2

[EMBnet-Server] Date: Sat May 9 0:31:33 2020

```
# bin/lalign36 -E 10.0 -f -12 -g -2 7505.1.seq 7505.2.seq -J -K 3
LALIGN finds non-overlapping local alignments
 version 36.3.5e Nov, 2012(preload8)
Please cite:
 X. Huang and W. Miller (1991) Adv. Appl. Math. 12:373-381

Query: 7505.1.seq
  1>>>E Protein SARS CoV 76 bp - 76 aa
Library: 7505.2.seq
       75 residues in     1 sequences

Statistics: (shuffled [500]) MLE statistics: Lambda= 0.1595;  K=0.09062
 statistics sampled from 1 (1) to 500 sequences
Threshold: E() < 10 score: 25
Algorithm: Smith-Waterman (SSE2, Michael Farrar 2006) (7.2 Nov 2010)
Parameters: BL50 matrix (15:-5), open/ext: -12/-2
 Scan time:  0.000

>>E Protein SARS CoV-2 75 bp                              (75 aa)
 Waterman-Eggert score: 431;  102.7 bits; E(1) <  7.1e-28
94.7% identity (97.4% similar) in 76 aa overlap (1-76:1-75)

               10        20        30        40        50        60
E      MYSFVSEETGTLIVNSVLLFLAFVVFLLVTLAILTALRLCAYCCNIVNVSLVKPTVYVYS
       :::::::::::::::::::::::::::::::::::::::::::::::::::::::. ::::
E      MYSFVSEETGTLIVNSVLLFLAFVVFLLVTLAILTALRLCAYCCNIVNVSLVKPSFYVYS
               10        20        30        40        50        60

               70
E      RVKNLNSSEGVPDLLV
       ::::::::. ::::::
E      RVKNLNSSR-VPDLLV
                70

>--
 Waterman-Eggert score: 43;  13.4 bits; E(1) <  0.42
27.6% identity (51.7% similar) in 29 aa overlap (21-49:18-46)

               30        40
E      LAFVVFLLVTLAILTALRLCAYCCNIVNV
       : :..:..  :. :. :     :    :.
E      LLFLAFVVFLLVTLAILTALRLCAYCCNI
         20        30        40

>--
 Waterman-Eggert score: 43;  13.4 bits; E(1) <  0.42
27.6% identity (51.7% similar) in 29 aa overlap (18-46:21-49)

         20        30        40
E      LLFLAFVVFLLVTLAILTALRLCAYCCNI
```

```
       : :..:..  :. :. :     :    :.
E      LAFVVFLLVTLAILTALRLCAYCCNIVNV
               30        40



76 residues in 1 query   sequences
75 residues in 1 library sequences
 Scomplib [36.3.5e Nov, 2012(preload8)]
 start: Sat May  9 00:31:33 2020 done: Sat May  9 00:31:33 2020
 Total Scan time:  0.000 Total Display time:  0.010

Function used was LALIGN [36.3.5e Nov, 2012(preload8)]
```

lalign output for MEMBRANE PROTEIN SARS COV vs. MEMBRANE PROTEIN SARS COV2

[EMBnet-Server] Date: Sat May 9 0:33:11 2020

```
# bin/lalign36 -E 10.0 -f -12 -g -2 6674.1.seq 6674.2.seq -J -K 3
LALIGN finds non-overlapping local alignments
 version 36.3.5e Nov, 2012(preload8)
Please cite:
 X. Huang and W. Miller (1991) Adv. Appl. Math. 12:373-381

Query: 6674.1.seq
  1>>>Membrane Protein SARS CoV 221 bp - 221 aa
Library: 6674.2.seq
      222 residues in     1 sequences

Statistics: (shuffled [500]) MLE statistics: Lambda= 0.1683;  K=0.02984
 statistics sampled from 1 (1) to 500 sequences
Threshold: E() < 10 score: 30
Algorithm: Smith-Waterman (SSE2, Michael Farrar 2006) (7.2 Nov 2010)
Parameters: BL50 matrix (15:-5), open/ext: -12/-2
 Scan time:  0.010

>>Membrane Protein SARS CoV-2 222 bp                      (222 aa)
 Waterman-Eggert score: 1344;  331.3 bits; E(1) <  9.1e-96
90.5% identity (98.2% similar) in 222 aa overlap (1-221:1-222)

                10        20        30        40        50
Membra MAD-NGTITVEELKQLLEQWNLVIGFLFLAWIMLLQFAYSNRNRFLYIIKLVFLWLLWPV
       ::: ::::::::::.::::::::::::::.:: ::::::.:::::::::::.::::::::
Membra MADSNGTITVEELKKLLEQWNLVIGFLFLTWICLLQFAYANRNRFLYIIKLIFLWLLWPV
               10        20        30        40        50        60

      60        70        80        90       100       110
Membra TLACFVLAAVYRINWVTGGIAIAMACIVGLMWLSYFVASFRLFARTRSMWSFNPETNILL
       :::::::::::::::.::::::::::.:::::::::.:::::::::::::::::::::::
Membra TLACFVLAAVYRINWITGGIAIAMACLVGLMWLSYFIASFRLFARTRSMWSFNPETNILL
               70        80        90       100       110       120

     120       130       140       150       160       170
Membra NVPLRGTIVTRPLMESELVIGAVIIRGHLRMAGHSLGRCDIKDLPKEITVATSRTLSYYK
       ::::.:::.::::.::::::::::.:::::.::: :::::::::::::::::::::::::
Membra NVPLHGTILTRPLLESELVIGAVILRGHLRIAGHHLGRCDIKDLPKEITVATSRTLSYYK
             130       140       150       160       170       180

     180       190       200       210       220
Membra LGASQRVGTDSGFAAYNRYRIGNYKLNTDHAGSNDNIALLVQ
       :::::::. :::::::.:::::::::::::..:.::::::::
Membra LGASQRVAGDSGFAAYSRYRIGNYKLNTDHSSSSDNIALLVQ
             190       200       210       220

>--
 Waterman-Eggert score: 43;  15.5 bits; E(1) <  0.65
29.3% identity (46.6% similar) in 58 aa overlap (52-109:26-75)
```

```
               60        70        80        90       100
Membra FLWLLWPVTLACFVLAAVYRINWVTGGIAIAMACIVGLMWLSYFVASFRLFARTRSMW
       ::.: : . :  :. :   :. ..   :      .. :.:    .: : : :  :  :
Membra FLFLTW-ICLLQFAYANRNRFLYIIKLI------FLWLLW-PVTLACFVLAAVYRINW
           30         40        50                60        70

>--
 Waterman-Eggert score: 40;  14.8 bits; E(1) <  0.83
27.6% identity (46.6% similar) in 58 aa overlap (25-74:53-110)

           30         40        50                60        70
Membra FLFLAW-IMLLQFAYSNRNRFLYIIKLV------FLWLLW-PVTLACFVLAAVYRINW
       ::.: : . :  :. .   :. .:   .      .. :.:    .: : : :  :  :
Membra FLWLLWPVTLACFVLAAVYRINWITGGIAIAMACLVGLMWLSYFIASFRLFARTRSMW
              60        70        80        90       100       110



221 residues in 1 query   sequences
222 residues in 1 library sequences
 Scomplib [36.3.5e Nov, 2012(preload8)]
 start: Sat May  9 00:33:12 2020 done: Sat May  9 00:33:12 2020
 Total Scan time:  0.010 Total Display time:  0.010

Function used was LALIGN [36.3.5e Nov, 2012(preload8)]
```

lalign output for ORF6 PROTEIN SARS COV vs. ORF6 PROTEIN SARS COV2

[EMBnet-Server] Date: Sat May 9 0:34:43 2020

```
# bin/lalign36 -E 10.0 -f -12 -g -2 5098.1.seq 5098.2.seq -J -K 3
LALIGN finds non-overlapping local alignments
 version 36.3.5e Nov, 2012(preload8)
Please cite:
 X. Huang and W. Miller (1991) Adv. Appl. Math. 12:373-381

Query: 5098.1.seq
  1>>>ORF6 Protein SARS CoV 63 bp - 63 aa
Library: 5098.2.seq
       61 residues in     1 sequences

Statistics: (shuffled [500]) MLE statistics: Lambda= 0.2023;  K=0.1197
 statistics sampled from 1 (1) to 500 sequences
Threshold: E() < 10 score: 19
Algorithm: Smith-Waterman (SSE2, Michael Farrar 2006) (7.2 Nov 2010)
Parameters: BL50 matrix (15:-5), open/ext: -12/-2
 Scan time:  0.000

>>ORF6 Protein SARS CoV-2 61 bp                           (61 aa)
 Waterman-Eggert score: 289;  87.4 bits; E(1) <  1.9e-23
68.9% identity (93.4% similar) in 61 aa overlap (1-61:1-61)

               10        20        30        40        50        60
ORF6   MFHLVDFQVTIAEILIIIMRTFRIAIWNLDVIISSIVRQLFKPLTKKNYSELDDEEPMEL
       :::::::::::::.::::::::..::::: ::. :...: : ::..:::.::.::::.
ORF6   MFHLVDFQVTIAEILLIIMRTFKVSIWNLDYIINLIIKNLSKSLTENKYSQLDEEQPMEI
               10        20        30        40        50        60


ORF6   D
       :
ORF6   D


>--
 Waterman-Eggert score: 26;  10.6 bits; E(1) <  0.91
41.7% identity (75.0% similar) in 12 aa overlap (7-18:22-33)

         10
ORF6   FQVTIAEILIII
       :.:.: ..  ::
ORF6   FKVSIWNLDYII
              30

>--
 Waterman-Eggert score: 25;  10.4 bits; E(1) <  0.95
28.6% identity (71.4% similar) in 14 aa overlap (33-46:29-42)

             40
```

```
ORF6   ISSIVRQLFKPLTK
       .. :.  ..: :.:
ORF6   LDYIINLIIKNLSK
       30        40



63 residues in 1 query   sequences
61 residues in 1 library sequences
 Scomplib [36.3.5e Nov, 2012(preload8)]
 start: Sat May  9 00:34:44 2020 done: Sat May  9 00:34:44 2020
 Total Scan time:  0.000 Total Display time:  0.000

Function used was LALIGN [36.3.5e Nov, 2012(preload8)]
```

lalign output for ORF7A PROTEIN SARS COV vs. ORF7A PROTEIN SARS COV2

[EMBnet-Server] Date: Sat May 9 0:38:30 2020

```
# bin/lalign36 -E 10.0 -f -12 -g -2 5095.1.seq 5095.2.seq -J -K 3
LALIGN finds non-overlapping local alignments
 version 36.3.5e Nov, 2012(preload8)
Please cite:
 X. Huang and W. Miller (1991) Adv. Appl. Math. 12:373-381

Query: 5095.1.seq
  1>>>ORF7a Protein SARS CoV 122 bp - 122 aa
Library: 5095.2.seq
      121 residues in     1 sequences

Statistics: (shuffled [500]) MLE statistics: Lambda= 0.1933;  K=0.06825
 statistics sampled from 1 (1) to 500 sequences
Threshold: E() < 10 score: 24
Algorithm: Smith-Waterman (SSE2, Michael Farrar 2006) (7.2 Nov 2010)
Parameters: BL50 matrix (15:-5), open/ext: -12/-2
 Scan time:  0.000

>>ORF7a Protein SARS CoV-2 121 bp                         (121 aa)
 Waterman-Eggert score: 702;  199.6 bits; E(1) <  1.2e-56
85.2% identity (95.9% similar) in 122 aa overlap (1-122:1-121)

               10        20        30        40        50        60
ORF7a  MKIILFLTLIVFTSCELYHYQECVRGTTVLLKEPCPSGTYEGNSPFHPLADNKFALTCTS
       :::::::.::...::::::::::::::::::::: ::::::::::::::::::::::: :
ORF7a  MKIILFLALITLATCELYHYQECVRGTTVLLKEPCSSGTYEGNSPFHPLADNKFALTCFS
               10        20        30        40        50        60

               70        80        90       100       110       120
ORF7a  THFAFACADGTRHTYQLRARSVSPKLFIRQEEVQQELYSPLFLIVAALVFLILCFTIKRK
       :.::::: ::..:.:::::::::::::::::::: ::::.:::::::.::. ::::.:::
ORF7a  TQFAFACPDGVKHVYQLRARSVSPKLFIRQEEVQ-ELYSPIFLIVAAIVFITLCFTLKRK
               70        80        90        100       110


ORF7a  TE
       ::
ORF7a  TE
     120

>--
 Waterman-Eggert score: 34;  13.4 bits; E(1) <  0.76
40.0% identity (53.3% similar) in 15 aa overlap (57-71:14-27)

         60        70
ORF7a  TCTSTHFAFACADGT
       ::   :.   :. ::
ORF7a  TCELYHYQ-ECVRGT
            20
```

```
>--
 Waterman-Eggert score: 31;  12.5 bits; E(1) <  0.92
34.8% identity (52.2% similar) in 23 aa overlap (96-118:41-62)

         100       110
ORF7a  ELYSPLFLIVAALVFLILCFTIK
       :  :: :  .:   : . ::. .
ORF7a  EGNSP-FHPLADNKFALTCFSTQ
                50        60



122 residues in 1 query   sequences
121 residues in 1 library sequences
 Scomplib [36.3.5e Nov, 2012(preload8)]
 start: Sat May  9 00:38:30 2020 done: Sat May  9 00:38:30 2020
 Total Scan time:  0.000 Total Display time:  0.010

Function used was LALIGN [36.3.5e Nov, 2012(preload8)]
```

lalign output for ORF7B PROTEIN SARS COV vs. ORF7B PROTEIN SARS COV2

[EMBnet-Server] Date: Sat May 9 13:48:05 2020

```
# bin/lalign36 -E 10.0 -f -12 -g -2 13128.1.seq 13128.2.seq -J -K 3
LALIGN finds non-overlapping local alignments
 version 36.3.5e Nov, 2012(preload8)
Please cite:
 X. Huang and W. Miller (1991) Adv. Appl. Math. 12:373-381

Query: 13128.1.seq
  1>>>ORF7b Protein SARS CoV 44 bp - 44 aa
Library: 13128.2.seq
       43 residues in     1 sequences

Statistics: (shuffled [500]) MLE statistics: Lambda= 0.1541;  K=0.2688
 statistics sampled from 1 (1) to 500 sequences
Threshold: E() < 10 score: 25
Algorithm: Smith-Waterman (SSE2, Michael Farrar 2006) (7.2 Nov 2010)
Parameters: BL50 matrix (15:-5), open/ext: -12/-2
 Scan time:  0.000

>>ORF7b Protein SARS CoV-2 43 bp                           (43 aa)
 Waterman-Eggert score: 234;  53.9 bits; E(1) <  1.1e-13
85.4% identity (92.7% similar) in 41 aa overlap (1-41:1-41)

               10        20        30        40
ORF7b  MNELTLIDFYLCFLAFLLFLVLIMLIIFWFSLEIQDLEEPC
       : ::.:::::::::::::::::::::::::::::.:: .: :
ORF7b  MIELSLIDFYLCFLAFLLFLVLIMLIIFWFSLELQDHNETC
               10        20        30        40

>--
 Waterman-Eggert score: 50;  13.0 bits; E(1) <  0.2
43.8% identity (87.5% similar) in 16 aa overlap (13-28:10-25)

             20
ORF7b  FLAFLLFLVLIMLIIF
       .: :: ::....::..
ORF7b  YLCFLAFLLFLVLIML
      10        20

>--
 Waterman-Eggert score: 50;  13.0 bits; E(1) <  0.2
43.8% identity (87.5% similar) in 16 aa overlap (10-25:13-28)

      10        20
ORF7b  YLCFLAFLLFLVLIML
       .: :: ::....::..
ORF7b  FLAFLLFLVLIMLIIF
             20
```

```
44 residues in 1 query   sequences
43 residues in 1 library sequences
 Scomplib [36.3.5e Nov, 2012(preload8)]
 start: Sat May  9 13:48:06 2020 done: Sat May  9 13:48:06 2020
 Total Scan time:  0.000 Total Display time:  0.000

Function used was LALIGN [36.3.5e Nov, 2012(preload8)]
```

Readseq version 2.1.30 (12-May-2010) Readseq version 2.1.30 (12-May-2010)

lalign output for ORF8A PROTEIN SARS COV vs. ORF8B PROTEIN SARS COV

[EMBnet-Server] Date: Sat May 9 13:50:16 2020

```
# bin/lalign36 -E 10.0 -f -12 -g -2 14140.1.seq 14140.2.seq -J -K 3
LALIGN finds non-overlapping local alignments
 version 36.3.5e Nov, 2012(preload8)
Please cite:
 X. Huang and W. Miller (1991) Adv. Appl. Math. 12:373-381

Query: 14140.1.seq
  1>>>ORF8a Protein SARS CoV 39 bp - 39 aa
Library: 14140.2.seq
       84 residues in     1 sequences

Statistics: (shuffled [500]) MLE statistics: Lambda= 0.2308;  K=0.1984
 statistics sampled from 1 (1) to 500 sequences
Threshold: E() < 10 score: 18
Algorithm: Smith-Waterman (SSE2, Michael Farrar 2006) (7.2 Nov 2010)
Parameters: BL50 matrix (15:-5), open/ext: -12/-2
 Scan time:  0.000

>>ORF8b Protein SARS CoV 84 bp                            (84 aa)
 Waterman-Eggert score: 43;  16.7 bits; E(1) <  0.031
41.2% identity (58.8% similar) in 17 aa overlap (18-34:35-51)

        20        30
ORF8a  TVVQRCASNKPHVLEDP
       :.:: :. :     .::
ORF8b  TMVQTCTPNVTINCQDP
           40        50

>--
 Waterman-Eggert score: 29;  12.0 bits; E(1) <  0.55
23.1% identity (42.3% similar) in 26 aa overlap (14-39:39-64)

            20        30
ORF8a  SCICTVVQRCASNKPHVLEDPCKVQH
       .:  .:.  : .    .:   :   :
ORF8b  TCTPNVTINCQDPAGGALIARCWYLH
       40        50        60

>--
 Waterman-Eggert score: 27;  11.3 bits; E(1) <  0.72
15.0% identity (60.0% similar) in 20 aa overlap (16-35:21-40)

          20        30
ORF8a  ICTVVQRCASNKPHVLEDPC
       .:.. .    .. :.. . :
ORF8b  LCALGKVLPFHRWHTMVQTC
```

```
               30        40



39 residues in 1 query   sequences
84 residues in 1 library sequences
 Scomplib [36.3.5e Nov, 2012(preload8)]
 start: Sat May  9 13:50:17 2020 done: Sat May  9 13:50:17 2020
 Total Scan time:  0.000 Total Display time:  0.000

Function used was LALIGN [36.3.5e Nov, 2012(preload8)]
```

Readseq version 2.1.30 (12-May-2010) Readseq version 2.1.30 (12-May-2010)

lalign output for ORF8A PROTEIN SARS COV vs. ORF8 PROTEIN SARS COV2

[EMBnet-Server] Date: Sat May 9 13:51:34 2020

```
# bin/lalign36 -E 10.0 -f -12 -g -2 7581.1.seq 7581.2.seq -J -K 3
LALIGN finds non-overlapping local alignments
 version 36.3.5e Nov, 2012(preload8)
Please cite:
 X. Huang and W. Miller (1991) Adv. Appl. Math. 12:373-381

Query: 7581.1.seq
  1>>>ORF8a Protein SARS CoV 39 bp - 39 aa
Library: 7581.2.seq
      121 residues in     1 sequences

Statistics: (shuffled [500]) MLE statistics: Lambda= 0.1895;  K=0.08093
 statistics sampled from 1 (1) to 500 sequences
Threshold: E() < 10 score: 19
Algorithm: Smith-Waterman (SSE2, Michael Farrar 2006) (7.2 Nov 2010)
Parameters: BL50 matrix (15:-5), open/ext: -12/-2
 Scan time:  0.000

>>ORF8 Protein SARS CoV-2 121 bp                          (121 aa)
 Waterman-Eggert score: 89;  28.0 bits; E(1) <  1.8e-05
31.7% identity (70.7% similar) in 41 aa overlap (1-38:1-40)

               10           20        30
ORF8a  MKLLIVLTCISLCSCI---CTVVQRCASNKPHVLEDPCKVQ
       ::.:. :  :.  . .   :.. : :....:.:..::: ..
ORF8   MKFLVFLGIITTVAAFHQECSL-QSCTQHQPYVVDDPCPIH
               10        20         30        40

>--
 Waterman-Eggert score: 26;  10.7 bits; E(1) <  0.94
42.9% identity (85.7% similar) in 7 aa overlap (18-24:97-103)

        20
ORF8a  TVVQRCA
       ..: ::.
ORF8   SLVVRCS
        100

>--
 Waterman-Eggert score: 25;  10.5 bits; E(1) <  0.96
16.7% identity (66.7% similar) in 18 aa overlap (7-24:4-21)

         10        20
ORF8a  LTCISLCSCICTVVQRCA
       :. ... . . .  :.:.
ORF8   LVFLGIITTVAAFHQECS
```

```
            10        20



39 residues in 1 query   sequences
121 residues in 1 library sequences
 Scomplib [36.3.5e Nov, 2012(preload8)]
 start: Sat May  9 13:51:35 2020 done: Sat May  9 13:51:35 2020
 Total Scan time:  0.000 Total Display time:  0.000

Function used was LALIGN [36.3.5e Nov, 2012(preload8)]
```

Readseq version 2.1.30 (12-May-2010) Readseq version 2.1.30 (12-May-2010)

lalign output for ORF8B PROTEIN SARS COV vs. ORF8 PROTEIN SARS COV2

[EMBnet-Server] Date: Sat May 9 13:54:25 2020

```
# bin/lalign36 -E 10.0 -f -12 -g -2 14902.1.seq 14902.2.seq -J -K 3
LALIGN finds non-overlapping local alignments
 version 36.3.5e Nov, 2012(preload8)
Please cite:
 X. Huang and W. Miller (1991) Adv. Appl. Math. 12:373-381

Query: 14902.1.seq
  1>>>ORF8b Protein SARS CoV 84 bp - 84 aa
Library: 14902.2.seq
      121 residues in     1 sequences

Statistics: (shuffled [500]) MLE statistics: Lambda= 0.2293;  K=0.1147
 statistics sampled from 1 (1) to 500 sequences
Threshold: E() < 10 score: 21
Algorithm: Smith-Waterman (SSE2, Michael Farrar 2006) (7.2 Nov 2010)
Parameters: BL50 matrix (15:-5), open/ext: -12/-2
 Scan time:  0.000

>>ORF8 Protein SARS CoV-2 121 bp                          (121 aa)
 Waterman-Eggert score: 100;  36.2 bits; E(1) <  1.3e-07
40.5% identity (66.7% similar) in 42 aa overlap (39-80:82-119)

       40        50        60        70        80
ORF8b  TCTPNVTINCQDPAGGALIARCWYLHEGHQTAAFRDVLVVLN
       .: :  :::::.:  :.:..:: . ..       ..:: :::.
ORF8   SCLP-FTINCQEPKLGSLVVRCSFYED---FLEYHDVRVVLD
               90       100           110

>--
 Waterman-Eggert score: 58;  22.3 bits; E(1) <  0.002
24.5% identity (54.7% similar) in 53 aa overlap (19-71:5-55)

       20        30        40        50        60        70
ORF8b  AWLCALGKVLPFHRWHTMVQTCTPNVTINCQDPAGGALIARCWYLHEGHQTAA
       ..:  .  :  ::.  .. :.:: .      .::     . .. ::.. : . .:
ORF8   VFLGIITTVAAFHQECSL-QSCTQHQPYVVDDPCPIHFYSK-WYIRVGARKSA
           10        20          30        40          50

>--
 Waterman-Eggert score: 35;  14.7 bits; E(1) <  0.32
26.9% identity (53.8% similar) in 26 aa overlap (30-53:42-66)

      30          40        50
ORF8b  FHRWHTMV--QTCTPNVTINCQDPAG
       . .:.  :  .  .: . . : : ::
ORF8   YSKWYIRVGARKSAPLIEL-CVDEAG
```

```
              50        60




84 residues in 1 query   sequences
121 residues in 1 library sequences
 Scomplib [36.3.5e Nov, 2012(preload8)]
 start: Sat May  9 13:54:26 2020 done: Sat May  9 13:54:26 2020
 Total Scan time:  0.000 Total Display time:  0.010

Function used was LALIGN [36.3.5e Nov, 2012(preload8)]
```

Readseq version 2.1.30 (12-May-2010) Readseq version 2.1.30 (12-May-2010)

lalign output for N PROTEIN SARS COV vs. N PROTEIN SARS COV2

[EMBnet-Server] Date: Sat May 9 14:11:12 2020

```
# bin/lalign36 -E 10.0 -f -12 -g -2 6576.1.seq 6576.2.seq -J -K 3
LALIGN finds non-overlapping local alignments
 version 36.3.5e Nov, 2012(preload8)
Please cite:
 X. Huang and W. Miller (1991) Adv. Appl. Math. 12:373-381

Query: 6576.1.seq
  1>>>N Protein SARS CoV 422 bp - 422 aa
Library: 6576.2.seq
      419 residues in     1 sequences

Statistics: (shuffled [500]) MLE statistics: Lambda= 0.1088;  K=0.009123
 statistics sampled from 1 (1) to 500 sequences
Threshold: E() < 10 score: 47
Algorithm: Smith-Waterman (SSE2, Michael Farrar 2006) (7.2 Nov 2010)
Parameters: BL50 matrix (15:-5), open/ext: -12/-2
 Scan time:  0.040

>>N Protein SARS CoV-2 419 bp                             (419 aa)
 Waterman-Eggert score: 2573;  410.8 bits; E(1) <  3.8e-119
90.5% identity (97.2% similar) in 422 aa overlap (1-422:1-419)

               10        20        30        40        50        60
N      MSDNGPQSNQRSAPRITFGGPTDSTDNNQNGGRNGARPKQRRPQGLPNNTASWFTALTQH
       ::::::: :::.:::::::::.::: .:::: :.::: ::::::::::::::::::::::
N      MSDNGPQ-NQRNAPRITFGGPSDSTGSNQNGERSGARSKQRRPQGLPNNTASWFTALTQH
                10        20        30        40        50

               70        80        90       100       110       120
N      GKEELRFPRGQGVPINTNSGPDDQIGYYRRATRRVRGGDGKMKELSPRWYFYYLGTGPEA
       :::.:.:::::::::::::.::::::::::::::.::::::::.::::::::::::::::
N      GKEDLKFPRGQGVPINTNSSPDDQIGYYRRATRRIRGGDGKMKDLSPRWYFYYLGTGPEA
      60        70        80        90       100       110

              130       140       150       160       170       180
N      SLPYGANKEGIVWVATEGALNTPKDHIGTRNPNNNAATVLQLPQGTTLPKGFYAEGSRGG
       .:::::::.::.:::::::::::::::::::: :::: ::::::::::::::::::::::
N      GLPYGANKDGIIWVATEGALNTPKDHIGTRNPANNAAIVLQLPQGTTLPKGFYAEGSRGG
     120       130       140       150       160       170

              190       200       210       220       230       240
N      SQASSRSSSRSRGNSRNSTPGSSRGNSPARMASGGGETALALLLLDRLNQLESKVSGKGQ
       ::::::::::::..:::::::::::.::::::..::.:.:::::::::::::::.:::::
N      SQASSRSSSRSRNSSRNSTPGSSRGTSPARMAGNGGDAALALLLLDRLNQLESKMSGKGQ
     180       190       200       210       220       230

              250       260       270       280       290       300
N      QQQGQTVTKKSAAEASKKPRQKRTATKQYNVTQAFGRRGPEQTQGNFGDQDLIRQGTDYK
```

```
       :::::::::::::::::::::::::::: :::::::::::::::::::::.::::::
N      QQQGQTVTKKSAAEASKKPRQKRTATKAYNVTQAFGRRGPEQTQGNFGDQELIRQGTDYK
     240       250       260       270       280       290

             310       320       330       340       350       360
N      HWPQIAQFAPSASAFFGMSRIGMEVTPSGTWLTYHGAIKLDDKDPQFKDNVILLNKHIDA
       :::::::::::::::::::::::::::::::::: ::::::::::.:::.::::::::::
N      HWPQIAQFAPSASAFFGMSRIGMEVTPSGTWLTYTGAIKLDDKDPNFKDQVILLNKHIDA
     300       310       320       330       340       350

             370       380       390       400       410       420
N      YKTFPPTEPKKDKKKKTDEAQPLPQRQKKQPTVTLLPAADMDDFSRQLQNSMSGASADST
       ::::::::::::::::.::.: :::::::: :::::::::.::::.:::.:::  :::::
N      YKTFPPTEPKKDKKKKADETQALPQRQKKQQTVTLLPAADLDDFSKQLQQSMS--SADST
     360       370       380       390       400       410


N      QA
       ::
N      QA


>--
 Waterman-Eggert score: 97;  22.0 bits; E(1) <  0.041
32.6% identity (61.8% similar) in 89 aa overlap (180-266:175-263)

     180       190       200       210        220       230
N      GSQASSRSSSRSRGNSRNSTPGSSRGNSPARM-ASGGGETALALLLLDRLNQLESKVSG-
       ::...:..:::: . ::::. .:. :.: .    :  .:. . : : :  :..:..  :
N      GSRGGSQASSRSSSRSRNSSRNSTPGSSRGTSPARMAGNGGDAALALLLLDRLNQLESKM
          180       190       200       210       220       230

       240       250       260
N      KGQQQQGQTVTKKSAAEASKKPRQKRTAT
       .:. :: :  :  . . :  . . ..  :
N      SGKGQQQQGQTVTKKSAAEASKKPRQKRT
          240       250       260

>--
 Waterman-Eggert score: 90;  20.9 bits; E(1) <  0.086
31.9% identity (58.5% similar) in 94 aa overlap (176-264:179-265)

         180       190           200       210       220        230
N      GSRGGSQASSRSSSRSR----GNSRNSTPGSSRGNSPARMASGGGETALALL-LLDRLNQ
       ::...:..:::: . ::    :.::...:.   ::.       : .  :: ::  :..:..
N      GSQASSRSSSRSRNSSRNSTPGSSRGTSPARMAGNG------GDAALALLLLDRLNQLES
      180       190       200       210              220       230

             240       250       260
N      LESKVSGKGQQQQGQTVTKKSAAEASKKPRQKRT
         :  .:. :: :  :  . . :  . . ..  :
N      KMSG-KGQQQQGQTVTKKSAAEASKKPRQKRTAT
             240       250       260


422 residues in 1 query   sequences
```

```
419 residues in 1 library sequences
 Scomplib [36.3.5e Nov, 2012(preload8)]
 start: Sat May  9 14:11:13 2020 done: Sat May  9 14:11:13 2020
 Total Scan time:  0.040 Total Display time:  0.050

Function used was LALIGN [36.3.5e Nov, 2012(preload8)]
```

Readseq version 2.1.30 (12-May-2010) Readseq version 2.1.30 (12-May-2010)

lalign output for ORF9B SARS COV vs. ORF10 SARS COV2

[EMBnet-Server] Date: Sat May 9 14:20:14 2020

```
# bin/lalign36 -E 10.0 -f -12 -g -2 7581.1.seq 7581.2.seq -J -K 3
LALIGN finds non-overlapping local alignments
 version 36.3.5e Nov, 2012(preload8)
Please cite:
 X. Huang and W. Miller (1991) Adv. Appl. Math. 12:373-381

Query: 7581.1.seq
  1>>>ORF9b SARS CoV 98 bp - 98 aa
Library: 7581.2.seq
       38 residues in     1 sequences

Statistics: (shuffled [500]) MLE statistics: Lambda= 0.2834;  K=0.1474
 statistics sampled from 1 (1) to 500 sequences
Threshold: E() < 10 score: 14
Algorithm: Smith-Waterman (SSE2, Michael Farrar 2006) (7.2 Nov 2010)
Parameters: BL50 matrix (15:-5), open/ext: -12/-2
 Scan time:  0.000

>>ORF10 SARS CoV-2 38 bp                                  (38 aa)
 Waterman-Eggert score: 25;  13.0 bits; E(1) <  0.37
28.6% identity (52.4% similar) in 21 aa overlap (41-61:5-25)

              50        60
ORF9b  KVYPIILRLGSQLSLSMARRN
       .:. . . . : :   :  ::
ORF10  NVFAFPFTIYSLLLCRMNSRN
           10        20

>--
 Waterman-Eggert score: 22;  11.8 bits; E(1) <  0.66
33.3% identity (83.3% similar) in 12 aa overlap (42-52:13-24)

              50
ORF9b  VYPIIL-RLGSQ
       .: ..: :..:.
ORF10  IYSLLLCRMNSR
             20

>--
 Waterman-Eggert score: 21;  11.3 bits; E(1) <  0.76
50.0% identity (62.5% similar) in 8 aa overlap (54-61:15-22)

          60
ORF9b  SLSMARRN
       :: . : :
ORF10  SLLLCRMN
           20
```

```
98 residues in 1 query   sequences
38 residues in 1 library sequences
 Scomplib [36.3.5e Nov, 2012(preload8)]
 start: Sat May  9 14:20:15 2020 done: Sat May  9 14:20:15 2020
 Total Scan time:  0.000 Total Display time:  0.000

Function used was LALIGN [36.3.5e Nov, 2012(preload8)]
```

Readseq version 2.1.30 (12-May-2010) Readseq version 2.1.30 (12-May-2010)

lalign output for ORF3B SARS COV vs. ORF3B SARS COV2

[EMBnet-Server] Date: Fri May 22 16:18:17 2020

```
# bin/lalign36 -E 10.0 -f -12 -g -2 21903.1.seq 21903.2.seq -J -K 3
LALIGN finds non-overlapping local alignments
 version 36.3.5e Nov, 2012(preload8)
Please cite:
 X. Huang and W. Miller (1991) Adv. Appl. Math. 12:373-381

Query: 21903.1.seq
  1>>>ORF3b SARS CoV 154 bp - 154 aa
Library: 21903.2.seq
       57 residues in     1 sequences

Statistics: (shuffled [500]) MLE statistics: Lambda= 0.1928;  K=0.06395
 statistics sampled from 1 (1) to 500 sequences
Threshold: E() < 10 score: 21
Algorithm: Smith-Waterman (SSE2, Michael Farrar 2006) (7.2 Nov 2010)
Parameters: BL50 matrix (15:-5), open/ext: -12/-2
 Scan time:  0.000

>>ORF3b SARS CoV-2 57 bp                                  (57 aa)
 Waterman-Eggert score: 32;  12.9 bits; E(1) <  0.69
26.5% identity (50.0% similar) in 34 aa overlap (102-134:3-36)

             110       120       130
ORF3b  MCKYTQSTALQELLIQQWIQFMMSRRRLLAC-LC
       .:    :  ..: . ..  :  :.   : .: ::
ORF3b  YCWRCTSCCFSERFQNHNPQKEMATSTLQGCSLC
             10        20        30

>--
 Waterman-Eggert score: 31;  12.6 bits; E(1) <  0.76
50.0% identity (87.5% similar) in 8 aa overlap (35-42:16-23)

           40
ORF3b  FQHQNSKK
       ::..: .:
ORF3b  FQNHNPQK
          20

>--
 Waterman-Eggert score: 26;  11.2 bits; E(1) <  0.98
21.7% identity (56.5% similar) in 23 aa overlap (90-112:9-31)

      90       100       110
ORF3b  SSSLTSLLKTHRMCKYTQSTALQ
       :  ..  ...:   :   ...::
ORF3b  SCCFSERFQNHNPQKEMATSTLQ
       10        20        30
```

```
154 residues in 1 query   sequences
57 residues in 1 library sequences
 Scomplib [36.3.5e Nov, 2012(preload8)]
 start: Fri May 22 16:18:17 2020 done: Fri May 22 16:18:17 2020
 Total Scan time:  0.000 Total Display time:  0.000

Function used was LALIGN [36.3.5e Nov, 2012(preload8)]
```

Readseq version 2.1.30 (12-May-2010) Readseq version 2.1.30 (12-May-2010)

lalign output for ORF14 SARS COV vs. ORF14 SARS COV2

[EMBnet-Server] Date: Thu May 21 14:42:09 2020

```
# bin/lalign36 -E 10.0 -f -12 -g -2 29912.1.seq 29912.2.seq -J -K 3
LALIGN finds non-overlapping local alignments
 version 36.3.5e Nov, 2012(preload8)
Please cite:
 X. Huang and W. Miller (1991) Adv. Appl. Math. 12:373-381

Query: 29912.1.seq
  1>>>ORF14 SARS CoV 70 bp - 70 aa
Library: 29912.2.seq
       73 residues in     1 sequences

Statistics: (shuffled [500]) MLE statistics: Lambda= 0.2080;  K=0.1528
 statistics sampled from 1 (1) to 500 sequences
Threshold: E() < 10 score: 21
Algorithm: Smith-Waterman (SSE2, Michael Farrar 2006) (7.2 Nov 2010)
Parameters: BL50 matrix (15:-5), open/ext: -12/-2
 Scan time:  0.000

>>ORF14 SARS CoV-2 73 bp                                 (73 aa)
 Waterman-Eggert score: 353;  108.6 bits; E(1) <  1e-29
77.1% identity (85.7% similar) in 70 aa overlap (1-70:1-70)

               10        20        30        40        50        60
ORF14  MLPPCYNFLKEQHCQKASTQREAEAAVKPLLAPHHVVAVIQEIQLLAAVGEILLLEWLAE
       ::  ::::::::::::::::. :::::::::.::::::..::::: :::::.::::::: 
ORF14  MLQSCYNFLKEQHCQKASTQKGAEAAVKPLLVPHHVVATVQEIQLQAAVGELLLLEWLAM
               10        20        30        40        50        60

               70
ORF14  VVKLPSRYCC
       .: :    ::
ORF14  AVMLLLLCCC
               70

>--
 Waterman-Eggert score: 34;  12.9 bits; E(1) <  0.48
22.6% identity (50.9% similar) in 53 aa overlap (12-64:3-55)

              20        30        40        50        60
ORF14  QHCQKASTQREAEAAVKPLLAPHHVVAVIQEIQLLAAVGEILLLEWLAEVVKL
       : : .   ... . :     :   :  ..   ...:.: :: :   ..:.. :
ORF14  QSCYNFLKEQHCQKASTQKGAEAAVKPLLVPHHVVATVQEIQLQAAVGELLLL
             10        20        30        40        50

>--
 Waterman-Eggert score: 27;  10.8 bits; E(1) <  0.94
```

```
29.2% identity (54.2% similar) in 24 aa overlap (8-31:30-53)

        10        20        30
ORF14  FLKEQHCQKASTQREAEAAVKPLL
       .:  .:   .  . . .:::  ::
ORF14  LLVPHHVVATVQEIQLQAAVGELL
      30        40        50



70 residues in 1 query   sequences
73 residues in 1 library sequences
 Scomplib [36.3.5e Nov, 2012(preload8)]
 start: Thu May 21 14:42:10 2020 done: Thu May 21 14:42:10 2020
 Total Scan time:  0.000 Total Display time:  0.000

Function used was LALIGN [36.3.5e Nov, 2012(preload8)]
```
